# Establishing community-wide DNA barcode references for conserving mangrove forests in China

**DOI:** 10.1186/s12870-021-03349-z

**Published:** 2021-12-04

**Authors:** Xiaomeng Mao, Wei Xie, Xinnian Li, Suhua Shi, Zixiao Guo

**Affiliations:** 1 State Key Laboratory of Biocontrol, Guangdong Key Lab of Plant Resources, Southern Marine Science and Engineering Guangdong Laboratory (Zhuhai), School of Life Sciences, Sun Yat-Sen University, Guangzhou, 510275 China; 2 Forevergen Biosciences Center, Guangzhou, China

**Keywords:** Mangroves, DNA barcoding, Genetic diversity, ITS2, *rbcL*, *trnH-psbA*

## Abstract

**Background:**

Mangrove ecosystems have been the focus of global attention for their crucial role in sheltering coastal communities and retarding global climate change by sequestering ‘blue carbon’. China is relatively rich in mangrove diversity, with one-third of the ca. 70 true mangrove species and a number of mangrove associate species occurring naturally along the country’s coasts. Mangrove ecosystems, however, are widely threatened by intensifying human disturbances and rising sea levels. DNA barcoding technology may help protect mangrove ecosystems by providing rapid species identification.

**Results:**

To investigate this potential, 898 plant specimens were collected from 33 major mangrove sites in China. Based on the morphologic diagnosis, the specimens were assigned to 72 species, including all 28 true mangrove species and all 12 mangrove associate species recorded in China. Three chloroplast DNA markers *rbcL*, *trnH-psbA*, *matK*, and one nuclear marker ITS2 were chosen to investigate the utility of using barcoding to identify these species. According to the criteria of barcoding gaps in genetic distance, sequence similarity, and phylogenetic monophyly, we propose that a single marker, ITS2, is sufficient to barcode the species of mangroves and their associates in China. Furthermore, *rbcL* or *trnH-psbA* can also be used to gather supplement confirming data. In using these barcodes, we revealed a very low level of genetic variation among geographic locations in the mangrove species, which is an alert to their vulnerability to climate and anthropogenic disturbances.

**Conclusion:**

We suggest using ITS2 to barcode mangrove species and terrestrial coastal plants in South China. The DNA barcode sequences we obtained would be valuable in monitoring biodiversity and the restoration of ecosystems, which are essential for mangrove conservation.

**Supplementary Information:**

The online version contains supplementary material available at 10.1186/s12870-021-03349-z.

## Introduction

Mangroves are well-known as crucial ecosystems restricted to the marine intertidal zone of tropical and subtropical areas [1]. Although plant species diversity is low in mangrove forests, they play an essential role in protecting coastal communities from hurricanes, retarding tidal transgression, supporting the coastal food web, purifying seawater, and sequestering carbon [2]. Despite their importance, however, mangrove forests are endangered globally by frequent anthropogenic activities, such as urban expansion, dyke construction, and overexploitation [3]. Actions to protect and restore mangrove forests have been popular globally. These actions call for a need for rapid species identification, biodiversity evaluation, and monitoring ecosystem dynamics.

A mangrove species is classified into true mangroves or mangrove associates, according to whether it occurs exclusively in intertidal environments or not. True mangrove species occur exclusively below high tide lines while mangrove associate species occur in both intertidal zones and inland terrestrial environments. In intertidal zones, mangrove associate species usually grow in high tide regions where are less inundated by seawater. In contrast, true mangrove species commonly occupy middle to low tide regions though they are also found in high tide regions. Although disputes have not been fully resolved, most mangrove species are assigned to true mangroves or mangrove associates without controversy [1, 2, 4]. According to Duke et al., there are 69 true mangrove species, together with 12 hybrids [2, 3]. Southeast Asia and North Australia form a hotspot of mangrove diversity. China has 23 indigenous true mangroves (excluding the hybrid *Sonneratia paracaseolaris*) and 12 native mangrove associates which distribute throughout the tropical and subtropical coasts of South China [5]. Twenty-four true mangroves (including some introduced species) are found within the 4000 ha mangrove forests of Hainan Island alone [5, 6]. In comparison, only 19 species have been found in the 140,000 ha mangrove forests of the Sundarbans, Bengal [7, 8], and 30 species have been found in the 17,000 ha mangrove area of the Daintree River, Australia [9]. Notably, mangrove diversity decreases as the latitude increases in China. At the northernmost margin of mangrove forests, in Leqing County, Zhejiang Province (28°25′N), only one true mangrove, *Kandelia obovata* is found [5].

DNA barcode technology remarkably improves the efficiency of species identification, compared with traditional morphology-based diagnostics [10–12]. The ITS2 marker was suggested using in barcoding plants and animals universally [13]. Several other barcodes were also proposed for different groups of plants [11, 14, 15]. For example, “*rbcL* + *matK*” was proposed for use as a core barcode for land plants [15], ITS and *trnH-psbA* were proposed for flowering plants [16], and ITS2 was proposed to replace ITS [17, 18]. Recently, multiple markers or even the whole chloroplast genome have been widely used as barcodes [12].

Mangrove species are polyphyletic in the Tree of Life. The markers to be used in identifying mangrove species should be easy enough to amplify and variable enough to resolve closely related species. Although ITS2 was suggested to be used in plants universally, it has not been tested in barcoding mangrove species. In this study, we collected samples of all mangrove species from 33 major mangrove sites in China. Coastal terrestrial plants living close to mangroves were also collected. Based on this comprehensive collection, we evaluated the performances of several candidate markers in barcoding mangrove species. We newly generated large amounts of reference sequences of these markers, providing valuable resources for conserving mangrove communities such as monitoring mangrove forests, science education, and inspecting illegal logging.

## Results

### Specimens and DNA sequences

**Fig. 1:**
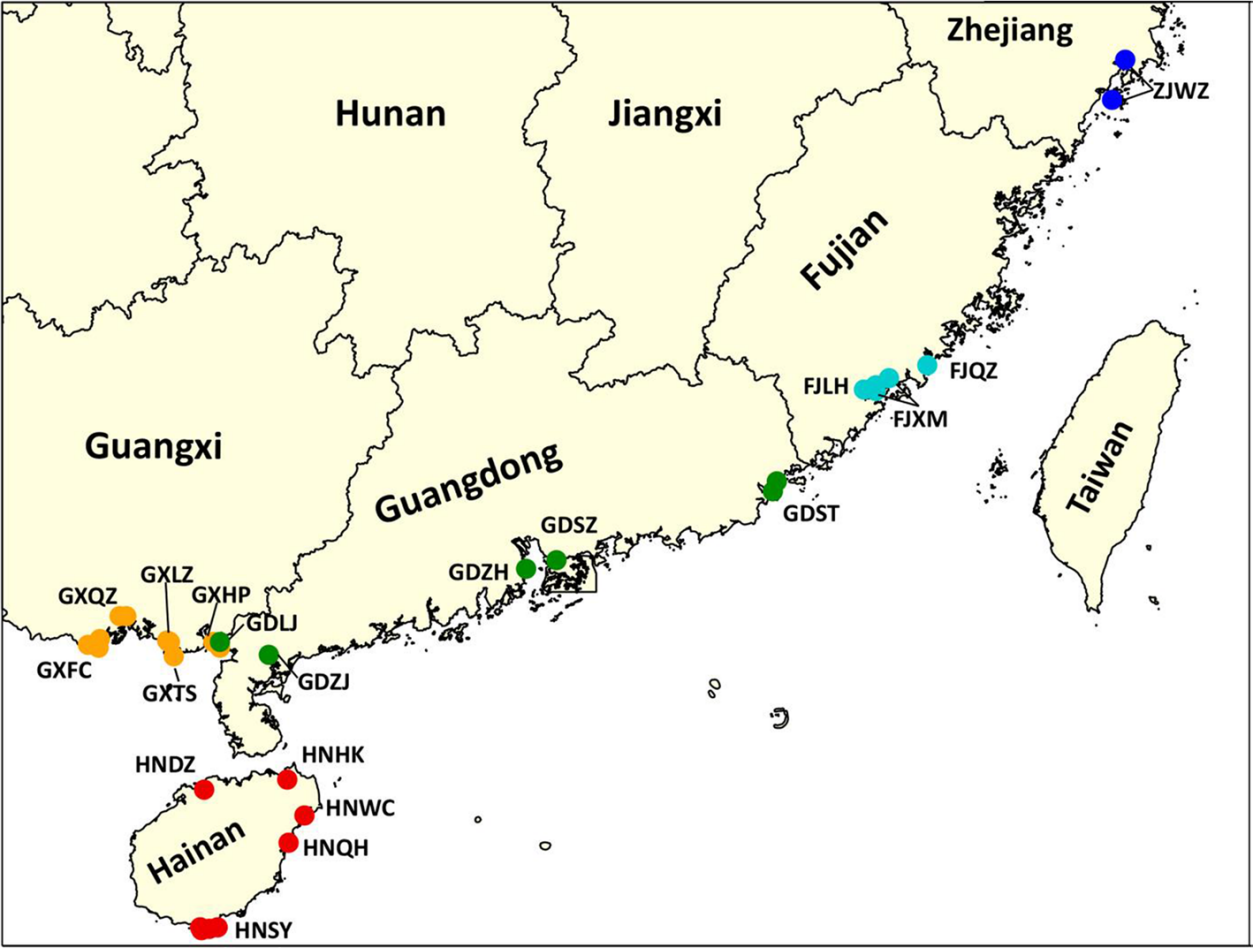
Map showing the mangrove sites we collected plant specimens in China. Different colors indicate sites in different provinces. The abbreviations indicate regions. In some regions, we collected specimens at more than one mangrove site, which are marked on the map but not numbered. GXFC: Fangchenggang, Guangxi; GXQZ: Qinzhou, Guangxi; GXTS: Tieshangang, Guangxi; GXLZ: Lianzhou Bay, Guangxi; GXHP: Hepu, Guangxi; GDLJ: Lianjiang, Guangdong; GDZJ: Zhanjiang, Guangdong; GDZH: Zhuhai, Guangdong; GDSZ: Shenzhen, Guangdong; GDST: Shantou, Guangdong; HNDZ: Danzhou, Hainan; HNSY: Sanya, Hainan; HNQH: Qionghai, Hainan; HNWC: Wenchang, Hainan; HNHK: Haikou, Hainan; FJLH: Longhai, Fujian; FJXM: Xiamen, Fujian; FJQZ: Quanzhou, Fujian; ZJWZ: Wenzhou, Zhejiang

We collected 898 plant specimens from 33 mangrove sites along the coastlines of South China, from Hainan island to northernmost Zhejiang (Fig. 1, Table 1). These samples represent all true mangroves and all mangrove associates recorded as being in China and some terrestrial coastal plants in South China. For each species, three to six individuals were collected from one site unless fewer than three individuals were found (Table 2). All the specimens were deposited in the herbarium of Sun Yat-sen University in Guangzhou. The specimens were assigned to 72 species based on morphological diagnostics, including 28 true mangroves, 12 mangrove associates, and 32 terrestrial coastal plants (Table 2).

**Table 1:** Information of specimens of mangrove species collected in 33 sites of South China coasts

Location	Site ID	Longitude & Latitude	Species	Sample size
Dongzhai Harbor, Haikou, Hainan	HNHK	110°36′E, 19°58′N	44	111
Qinglan Harbor, Wenchang, Hainan	HNWC	110°49′E, 19°33′N	25	68
Dongchang, Danzhou, Hainan	HNDZ	109°33′E, 19°51′N	19	52
Tielu Harbor, Sanya, Hainan	HNSY1	109°43′E, 18°15′N	20	53
Qingmei Harbor, Sanya, Hainan	HNSY2	109°37′E, 18°14′N	12	31
Yulin River, Sanya, Hainan	HNSY3	109°31′E, 18°13′N	13	29
Sanya River, Sanya, Hainan	HNSY4	109°30′E, 18°15′N	5	14
Tanmen, Qionghai, Hainan	HNQH	110°37′E, 19°14′N	2	8
Qi’ao island, Zhuhai, Guangdong	GDZH	113°37′E, 22°25′N	24	64
Futian Mangrove Natural Reserve, Guangdong	GDSZ	114°00′E, 22°31′N	21	54
Gaoqiao Mangrove Reserve, Zhanjiang, Guangdong	GDLJ	109°45′E, 21°34′N	13	39
Jilongshan, Zhanjiang, Guangdong	GDZJ	110°22′E, 21°25′N	16	46
Aotou, Shantou, Guangdong	GDST1	116°44′E, 23°19′N	7	23
Waisha River, Shantou, Guangdong	GDST2	116°47′E, 23°26′N	5	14
Gurong Tribe, Zhenzhu Bay, Fangchenggang, Guangxi	GXFC1	108°05′E, 21°32′N	14	45
Jiangshan, Zhenzhu Bay, Fangchenggang, Guangxi	GXFC2	108°13′E, 21°30′N	5	15
Shijiao Base, Zhenzhu Bay, Fangchenggang, Guangxi	GXFC3	108°14′E, 21°36′N	8	19
Kangxi Ridge, Maoweihai, Qinzhou, Guangxi	GXQZ1	108°29′E, 21°52′N	10	25
Haixialou, Maoweihai, Qinzhou, Guangxi	GXQZ2	108°34′E, 21°52′N	6	15
Yingluogang, Hepu, Beihai, Guangxi	GXHP1	109°45′E, 21°30′N	23	68
Dandou, Hepu, Beihai, Guangxi	GXHP2	109°40′E, 21°34′N	4	7
Xiaoguansha, Tieshangang, Beihai, Guangxi	GXTS	109°10′E, 21°24′N	2	6
Matou village, Lianzhou Harbor, Beihai, Guangxi	GXLZ1	109°07′E, 21°34′N	10	27
Zhenyudun, Lianzhou Harbor, Beihai, Guangxi	GXLZ2	109°06′E, 21°34′N	6	14
Duwuping, Lianzhou Harbor, Beihai, Guangxi	GXLZ3	109°05′E, 21°35′N	2	6
Haicangwan Park, Xiamen, Fujian	FJXM1	118°02′E, 24°29′N	5	19
Xiatanwei, Xiamen, Fujian	FJXM2	118°12′E, 24°38′N	3	12
Yuemeichi Park, Xiamen, Fujian	FJXM3	118°02′E, 24°33′N	6	14
Longhai Wetland Reserve, Longhai, Fujian	FJLH	117°53′E, 24°30′N	2	8
Quanzhou Bay Wetland Reserve, Quanzhou, Fujian	FJQZ	118°41′E, 24°47′N	2	7
Ximen Island, Wenzhou, Zhejiang	ZJWZ1	121°11′E, 28°20′N	1	4
Niyu Island, Wenzhou, Zhejiang	ZJWZ2	121°01′E, 27°52′N	2	8

**Table 2:** List of species and sites of specimens collected from coastal communities (mangrove forests) of South China

Group	Species	Hainan	Guangxi	Guangdong	Fujian	Zhejiang
True	*Acanthus ebracteatus*	+	+	+	+	
True	*Acanthus ilicifolius*		+	+		
True	*Acrostichum aureum*	+	+	+		
True	*Acrostichum speciosum*	+		+		
True	*Aegialitis annulata*	IN				
True	*Aegiceras corniculatum*	+	+	+	+	
True	*Avicennia germinans*	IN				
True	*Avicennia marina sub. marina*	+	+	+	+	
True	*Avicennia marina sub. australasica*	+				
True	*Avicennia marina sub. eucalyptifolia*	+				
True	*Bruguiera × rhychopetala*	HY				
True	*Bruguiera gymnorhiza*	+	+	+		
True	*Bruguiera sexangula*	+		+		
True	*Ceriops tagal*	+				
True	*Conocarpus erectus*			IN		
True	*Kandelia obovata*	+	+	+	+	IN
True	*Laguncularia racemosa*	+	+		+	
True	*Lumnitzera littorea*	+				
True	*Lumnitzera racemosa*	+	+	+		
True	*Nypa fruticans*	+				
True	*Pemphis acidula*	+				
True	*Rhizophora × larmarkii*	HY				
True	*Rhizophora apiculata*	+				
True	*Rhizophora mangle*	IN				
True	*Rhizophora mucronata*	IN				
True	*Rhizophora stylosa*	+	+	+		
True	*Scyphiphora hydrophyllacea*	+				
True	*Sonneratia × gulngai*	HY				
True	*Sonneratia × hainanensis*	HY				
True	*Sonneratia × zhongcairongii*	HY				
True	*Sonneratia alba*	+				
True	*Sonneratia apetala*	+	+	+	+	
True	*Sonneratia caseolaris*	+		+		
True	*Sonneratia ovata*	+				
True	*Xylocarpus granatum*	+				
Associate	*Barringtonia racemosa*	+		+		
Associate	*Cerbera manghas*	+	+	+		
Associate	*Clerodendrum inerme*	+	+	+		
Associate	*Dolichandrone spathacea*	+		+		
Associate	*Excoecaria agallocha*	+	+	+	+	
Associate	*Hernandia sonora*	+		+		
Associate	*Heritiera littoralis*	+	+	+		
Associate	*Hibiscus tiliaceus*	+	+	+		
Associate	*Pluchea indica*	+	+	+		
Associate	*Pongamia pinnata*	+	+	+		
Associate	*Premna obtusifolia*	+	+			
Associate	*Thespesia populnea*	+	+			
Coastal	*Abutilon indicum*	+		+		
Coastal	*Caesalpinia bonduc*	+				
Coastal	*Canavalia maritima*		+	+		
Coastal	*Cardiospermum halicacabum*	+				
Coastal	*Casuarina equisetifolia*		+	+		
Coastal	*Crotalaria pallida*	+				
Coastal	*Cyperus malaccensis*		+			
Coastal	*Dalbergia tonkinensis*	+				
Coastal	*Derris trifoliata*	+	+	+		
Coastal	*Hibiscus hamabo*			IN		
Coastal	*Hoya carnosa*	+				
Coastal	*Ipomoea pes-caprae*	+	+	+		
Coastal	*Leucaena leucocephala*			+		
Coastal	*Limonium sinense*		+			
Coastal	*Morinda citrifolia*	+				
Coastal	*Myoporum bontioides*		+	+		
Coastal	*Pandanus tectorius*	+	+	+		
Coastal	*Phyla nodiflora*		+			
Coastal	*Portulaca pilosa*		+			
Coastal	*Scaevola hainanensis*	+	+			
Coastal	*Scaevola sericea*	+	+			
Coastal	*Scirpus mariqueter*		+			
Coastal	*Sedum lineare*			+		
Coastal	*Sesbania cannabina*	+				
Coastal	*Sesuvium portulacastrum*		+		+	
Coastal	*Sida acuta*	+				
Coastal	*Stachytarpheta jamaicensis*	+				
Coastal	*Suaeda australis*		+			
Coastal	*Suaeda glauca*					+
Coastal	*Tephrosia purpurea*	+		+		
Coastal	*Trema tomentosa*	+				
Coastal	*Waltheria indica*	+				
Total		63	35	35	8	2

+ Natural distribution or local colonization, *IN* Introduced from other places, *HY* Hybrid types, *True* True mangroves, *Associate* Mangrove associates, *Coastal* Terrestrial coastal plants

**Fig. 2:**
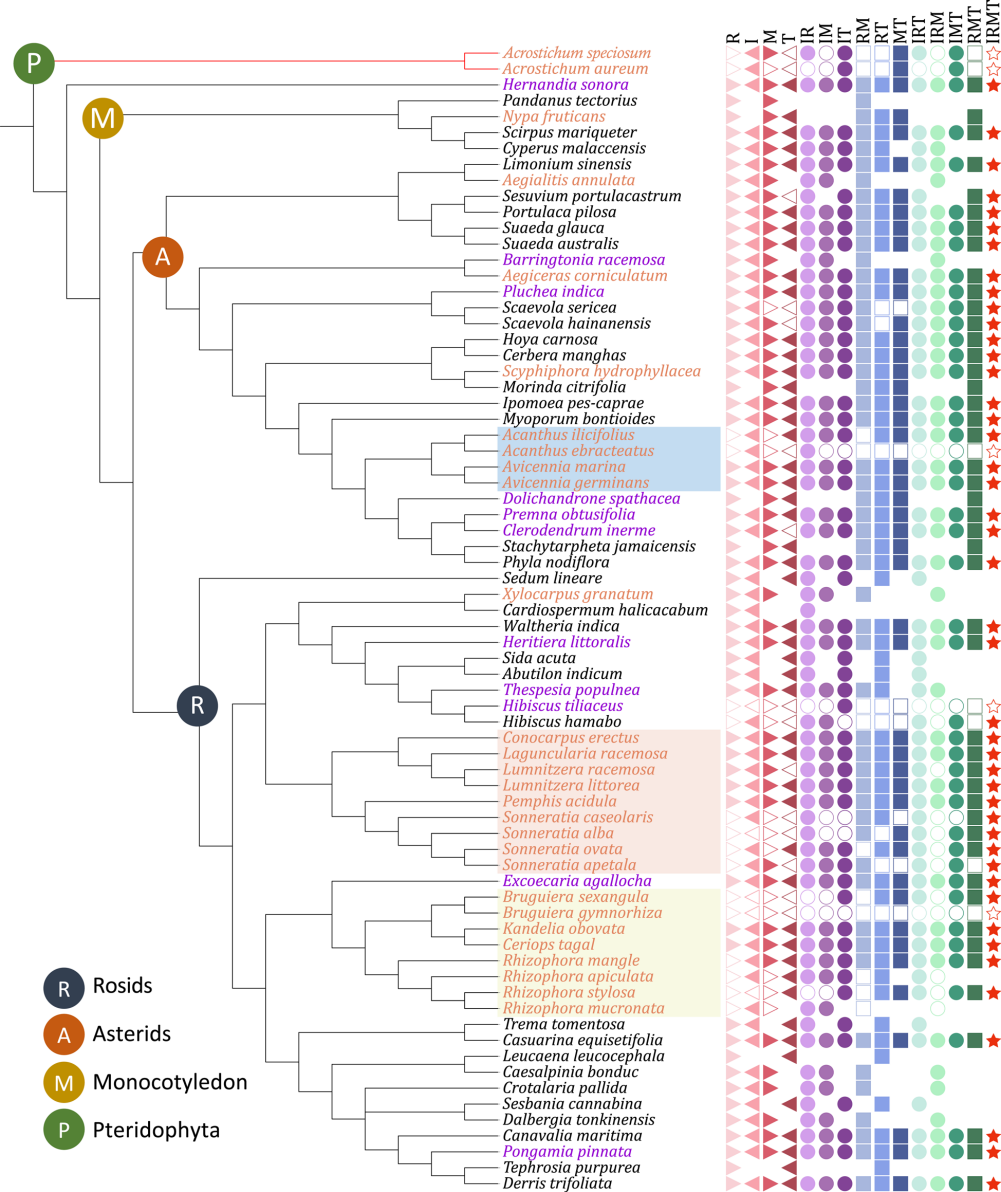
Phylogeny tree of coastal plants in South China. The cladogram relationships were inferred from public data, referencing the APGIV system. Icons in the right refer to whether the species could be successfully clustered in monophyly for the single-marker or multi-marker barcode. Solid icons indicate that all conspecific individuals formed a single clade with a bootstrap value > 50%. Hollow icons show failures in identification. Blanks indicate missing data. Different colors and shapes distinguish the 15 barcodes. Red species names indicate true mangroves, purple names indicate mangrove associate species and black names indicate terrestrial coastal plants. Background highlights are the main branches of true mangroves: Acanthaceae, Lythraceae, Combretaceae, Rhizophoraceae. Colored round icons on the nodes represent rosids (R), asterids (A), monocot (M), and pteridophytes (P), respectively

According to the Angiosperm Phylogeny Group system, the 72 species we collected belong to 58 genera, 32 families, and 19 orders (Fig. 2). Three plasmid barcoding markers *rbcL*, *matK*, *trnH-psbA*, and one nuclear barcoding marker ITS2 were amplified. The four markers varied in difficulty to amplify and sequence (Table 3). In total, 885, 789, 629, and 654 sequences were generated for *rbcL*, ITS2, *matK*, and *trnH-psbA*, respectively.

**Table 3:** Sequence characteristics of the four sequenced regions

	*rbcL*	ITS2	*matK*	*trnH-psbA*
Total number of sequences^a^	885	789	629	654
Success rate of PCR and sequencing	98.6%	87.9%	70.0%	72.8%
Number of species^b^	72	65	67	63
Percent with valid conspecifics	89.0%	90.8%	80.6%	88.9%
GC content	44.1	59.7	33.9	27.6
Number of variable sites	229	448	713	1072
Number of alignment gaps	NA	551	345	1402
Alignment length	570	679	902	1411
Maximum intraspecific distance	0.005	0.046	0.018	0.077
Maximum interspecific distance	0.267	0.705	0.835	1.481

^a^All sequences that have been successfully sequenced

^b^Sequences except for hybrids and variations

The amplicons of *rbcL* region were successfully sequenced in all species but in 98.6% of the individuals. Of the 570 bp long alignment, 40.2% was polymorphic. The amplicons of ITS2 were successfully sequenced in 87.9% of the individuals, with 66% of the 679 bp long alignment being polymorphic (Table 3). However, the amplicons of *matK* and *trnH-psbA* were successfully sequenced in only 70.0 and 72.8% of the individuals (Table 3). The low success rates are due to nonspecific binding of primers in amplification or difficulty of sequencing caused by simple sequence repeats (the repeat unit is one bp, for example, GGGGGGGGGGGGG). Additionally, the *trnH-psbA* genomic region showed a high level of length variation among species, such that 1402 sites of the 1411 bp alignment contain a gap in at least one individual. Hence, *rbcL* was the most successful marker in amplification and sequencing, and ITS2 was secondarily successful; *trnH-psbA* was less recommended to be applied as a barcode in these species.

### ITS2 could be used as a barcode for mangroves in China

According to the CBOL plant working group, an eligible barcode is expected to maintain high inter-species distance and low intra-species distance, which is called a barcode gap [19]. We employed three methods to evaluate the species discrimination power among barcodes. First, scatter plots were drawn to show minimum inter-species distance and maximum intra-species distance. The dot above the 1:1 slope was accepted as a successful barcode gap [20, 21]. The proportion of species with a successful barcode gap was used to evaluate the effectiveness of a barcode. In the Wilcoxon signed-rank test, ITS2 exhibited greater inter-species variation than *rbcL* and *matK* (Table 4). The four markers were then combined exhaustively to produce 11 combinations of multi-marker barcodes. For each combination, the sequences from the same individual were concatenated end-to-end. Hence, 15 candidate barcodes (four single-marker and 11 multi-marker barcodes) were used in the robustness test of species identification.

**Table 4:** Comparison of inter-specific genetic distance between markers using Wilcoxon signed test

Marker pairs		Relative ranks		N	*P*-value	Common language effect size	Results
before	after	W-	W+				
*rbcL*	ITS2	0	2,164,240	2080	2.20E-16	0.00	ITS2> > *rbcL*
*rbcL*	*matK*	6	2,027,085	2013	2.20E-16	0.00	*matK*> > *rbcL*
ITS2	*matK*	1,172,098	282,267	1705	2.20E-16	0.81	ITS2> > *matK*

**Fig. 3:**
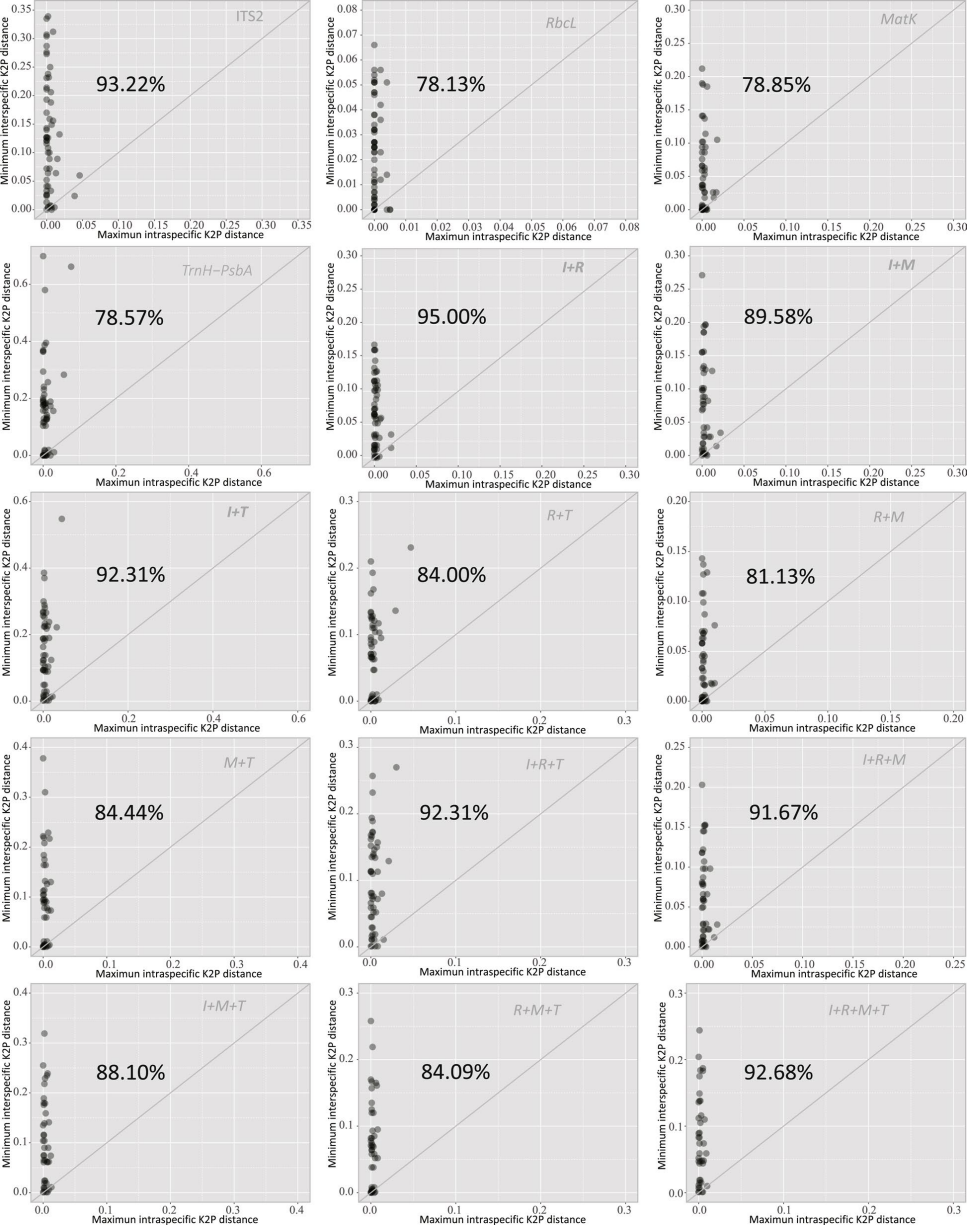
Scatter plots of the maximum intraspecific Kimura 2-parameter (K2P) distance versus minimum interspecific K2P distance for four single-marker barcodes and 11 multi-marker barcodes. The number in each subfigure indicates the percentage of points above the y = x oblique line. Abbreviations: I, ITS2; R, *rbcL*; M, *matK*; T, *trnH-psbA*

The scatter plot of interspecific and intraspecific distances indicates that most species show clear barcode gaps in all 15 barcodes (Fig. 3). Particularly, 93.2% of the species show a barcoding gap using the ITS2 marker alone (Table 5). The multi-marker barcode of ITS2 + *rbcL* improves the percentage to 95%. The other multi-marker barcodes involved with ITS2 also show high performance, e.g. ITS2 + *rbcL* + *MatK* + *trnH-psbA* (92.7%), ITS2 + *rbcL* + *trnH-psbA* (92.3%), and ITS2 + *trnH-psbA* (92.3%) (When referring to multi-marker barcodes in the following text, we use I, R, M, and T to represent ITS2, *rbcL*, *MatK*, and *trnH-psbA*, respectively). Hence, the ITS2 alone is sufficiently powerful as a barcode for these species.

**Table 5:** Success rates of species identification based on methods of genetic distance, similarity and phylogeny

Marker	Genetic distance	Similarity			Phylogeny		
	Barcode Gap (%)	Threshold	BM (%)	BCM (%)	BI (%)	NJ (%)	ML (%)
*rbcL*	78.1	0	68.5	67.6	77.8	79.2	77.8
*matK*	78.9	0.55	76.8	75.4	79.7	78.1	82.8
ITS2	93.2	0.59	93.0	92.4	92.3	92.3	93.9
*trnH-psbA*	78.6	0.92	83.7	82.8	82.5	77.8	82.5
I + R	95.0	0.4	93.3	92.3	92.3	93.9	92.3
I + T	92.3	0.83	99.1	97.9	87.7	93.0	93.0
I + M	89.6	0.75	92.0	91.4	91.5	91.4	87.9
R + M	81.1	0.47	85.1	83.7	82.8	85.9	82.8
R + T	84.0	0.81	91.4	90.5	87.3	84.1	88.9
M + T	84.4	0.61	96.0	95.2	84.9	86.8	86.8
I + R + M	91.7	0.6	92.1	91.5	93.2	77.6	87.9
I + R + T	92.3	0.65	98.9	97.7	94.7	96.5	93.0
I + M + T	88.1	0.57	98.4	97.3	89.8	91.7	91.7
R + M + T	84.1	0.69	96.0	95.6	90.6	90.6	86.8
R + I + M + T	92.7	0.59	98.2	97.5	93.9	95.9	89.8

*I* ITS2, *R rbcL*, *M matK*, *T trnH-psbA*

Secondly, the similarity between sequences is an important criterion in species assignment. To test the resolving power of the candidate DNA barcodes, the ‘Best Match’ (BM) and ‘Best Close Match’ (BCM) functions embedded in the program TaxonDNA v1.8 were implemented [22]. Only sequences with at least one valid conspecific sequence were included in this analysis. The similarity-based method also shows that ITS2 has a higher species resolving rate than the other three single-marker barcodes (Table 5). The BM ratio of ITS2 was 93.0% and the BCM ratio was 92.4%. Some of the multiple-marker barcodes showed higher rates, e.g. 99.1 and 97.9% of I + T, 98.9 and 97.7% of I + R + T, 98.9 and 97.7% of I + R + M + T.

Thirdly, the most straightforward way to describe a species’ discriminatory power is by constructing a phylogeny tree and evaluating the monophyly rate. We constructed trees for each candidate barcode using neighbor-joining (NJ), maximum likelihood (ML), and Bayesian inference (BI) methods. In the trees, species identification was considered successful only if all conspecific individuals form a monophyletic clade with a supporting rate greater than 50% (Table S1 in the supplementary materials). The proportion of monophyletic species was used to evaluate the performance of barcodes. The proportions were slightly different between the three methods (Table 5). To be conservative, we used the lowest one as the final value for each barcode. ITS2 is almost the most powerful marker for species discrimination (92.3%), although the combinations I + R and I + R + T show comparable power (92.3 and 93.0%). Hence, the phylogenetic tree method also confirmed ITS2 to be a good barcode in this dataset (Table 5).

In summary, the benchmark barcode in the *rbcL* of most land plants [23], showed a very low level of interspecific genetic variation among the species of mangroves. Moreover, *matK* and *trnH-psbA*, which were used to barcode 14 species of mangroves in India and 23 species in Guangdong, China, were only successfully amplified and sequenced in about 70% sampled individuals. Hence, by integrating all three methods we concluded that ITS2 alone could be used as the barcode for mangrove species in China. If ITS2 provides insufficient resolution in some circumstances, *rbcL* or/and *trnH-psbA* may be used to obtain supplemental data.

### Applicability of the barcode sequences

In total, nearly 3000 barcode sequences were obtained from *rbcL*, *trnH-psbA*, *matK*, and ITS2 regions. These sequences provide comprehensive references for use in species identification, biodiversity evaluation, and hybridization detection. We tested the applicability of these barcode sequences by analyzing hybrids previously recognized by morphology and the intraspecific subspecies of *Avicennia marina*.

Based on morphological diagnostics, four different hybrid combinations were recognized, namely *Sonneratia* × *zhongcairongii* [24], *S.* × *gulngai*, *S.* × *hainanensis*, and *Rhizophora* × *larmarkii*. By comparing the sequences of ITS2, we observed that the divergent sites of parental species were heterozygous in those individuals, providing genetic evidence of their hybrid status. The sequences also validated the parental origins of the hybrids. All three *Sonneratia* hybrids have *S. alba* as the matrilineal parent. The patrilineal parent of *S.* × *zhongcairongii* is *S. apetala,* with three divergent sites being heterozygous in the hybrid; the patrilineal parent of *S.* × *gulngai* is *S. caseolaris*, indicated by 11 heterozygous sites; and the patrilineal parent of *S.* × *hainanensis* is *S. ovata,* indicated by 12 heterozygous sites. Similarly, the hybrid *R.* × *larmarkii,* originating via hybridization between *R. apiculata* and *R. stylosa,* was evidenced by six heterozygous sites in the ITS2 region.

**Fig. 4:**
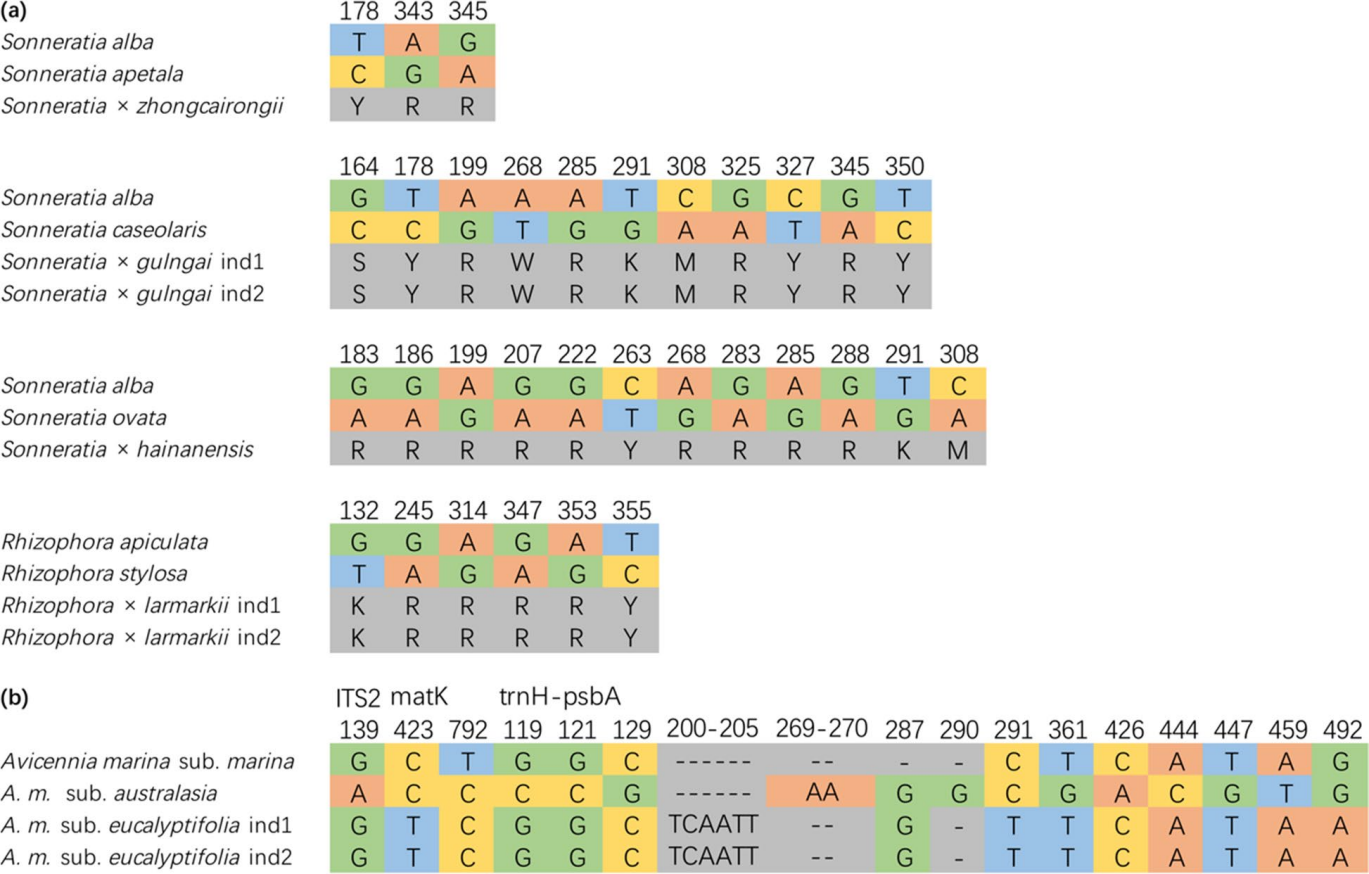
Polymorphic sites of hybrids and subspecies. **a** The alignment between four hybrids and their congeneric species in ITS2. **b** The alignment between the three subspecies of *Avicennia marina* in ITS2, *matK*, *trnH-psbA.* The “ind1” and “ind2” following species names indicate different individuals

The barcodes were also able to distinguish the three subspecies of *A. marina*. In ITS2, only one site distinguished *A. m. australasica* from the other two subspecies. Supplemented with *matK* and *trnH-psbA*, we identified 15 single nucleotide variants and two indels in these sequences. Based on these variants, three distinct haplotypes were inferred and each of the three subspecies contains one of the three haplotypes (Fig. 4). Hence, the DNA barcode sequences we obtained would be useful reference resources for future species identification.

### Genetic diversity of mangrove species estimated from the four barcoding markers

**Fig. 5:**
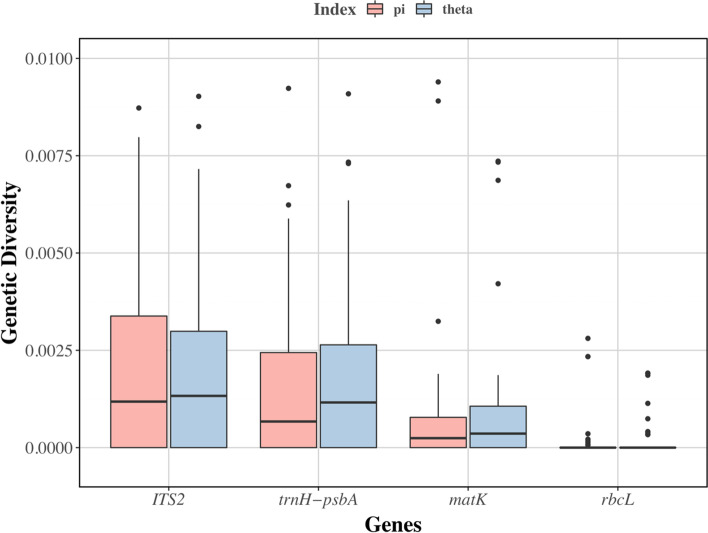
Box plot of population genetic diversity (θ and π) estimated from the four markers

To understand the level of genetic diversity in different species in the mangrove communities in South China, we first estimated Watterson’s estimator (θ) and nucleotide diversity (π) values for each population of each species [25]. Nearly all species show no intraspecific variations in the *rbcL* region, and several species show a considerable number of genetic variations in the other three regions. Particularly, the highest genetic diversity is in ITS2, with a slightly lower level in *trnH-psbA* (Fig. 5). Despite the highest level of variation, the average values of ITS2 are only ~ 0.001/bp; the highest outliers are also lower than 0.01/bp in both θ and π (Fig. 5). We further estimated the inter-population variations in pairwise populations. Consistently, *rbcL* shows low levels of variation between populations. In the ITS2, *matK*, and *trnH-psbA* regions, most species show a higher level of inter-population variation than intra-population variation (Figs. S1, S2, S3 and S4). There are also some species showing a low level of genetic variation within the range of the species. *Kandelia obovata*, *Avicennia marina*, *Aegiceras corniculatum,* and *Pluchea indica*, which have wider distribution ranges, show relatively higher levels of intraspecific genetic distance (Figs. S1, S2, S3 and S4).

## Discussion

South China, Southeast Asia, South Asia, and North Australia form the core of mangrove forests and the hotspot of mangroves species [1, 2, 26]. In China, 23 true mangorves occur natively on the southern coasts and six foreign species (including the four introduced species with only a few individuals planted in a particular site, namely, *Rhizophora mucronata*, *R. mangle*, *Avicennia germinans*, and *Conocarpus erectus*, Table 2) [5, 27]. There have been attempts to barcode species of mangroves in local regions. Saddhe et al. [28, 29] suggested using the combination of *matK* + ITS2 + *atpF-atpH* to barcode the 14 species of true mangroves on the west coast of India. Wu et al. [30] suggested using *rbcL* and *trnH-psbA* to barcode the 12 true mangroves and 11 mangrove associate species in Guangdong, China. Those studies focused on narrow local regions. Moreover, only one population was sampled to represent a species, without regard to intraspecific genetic variations. Our collection of 72 species included all species of true mangroves and all species of mangrove associates and a number of coastal but terrestrial plants in China. We sampled individuals from almost all mangrove sites in China so that natural genetic variations within species should have been comprehensively sampled.

Based on the criteria of the three methods, we recommend using ITS2 to barcode these species, with *rbcL* and *trnH-psbA* as supplements. The ITS2 marker was also been found to be efficient in barcoding other plant groups, such as the success rate of 93.3% in distinguishing 469 invasive plants [31], and the rate of 92.7% to distinguish 4800 medicinal plant species [17]. There was even a proposal that over 95% of the angiosperm, gymnosperms, ferns, lycophytes, mosses, and fungi could be distinguished using ITS2 [32]. ITS2 was proposed to be used as a universal barcode for identifying plant species and as a complementary locus for CO1 to identify animal species [13]. Our study proved that ITS2 outperformed the other three markers in distinguishing the species of mangroves. We also expanded the plant barcoding dataset with mangroves. The 2967 sequences of ITS2 and the other three markers, together with the 898 voucher specimens deposited in the herbarium of Sun Yat-Sen University, would provide the most complete references for conserving mangrove species worldwide.

Our DNA barcode reference sequences will be useful for rapid species identification without recourse to taxonomic experts. With reliable reference sequences, it will be accessible for verifying hybrids and infraspecific taxa using one or two markers. The convenience provided by rapid species identification will be applicable in many fields, including but are not limited to: (1) management practices such as monitoring the dynamics of mangrove forests, (2) citizen science education for environment protection, (3) wild plant protection through inspection for illegal logging; (4) scientific investigations conducted by researchers who are unfamiliar with plant systematics.

We showed the applicability of barcode resources in monitoring the genetic diversity of mangrove species at the community scale. We revealed a low level of intraspecific genetic diversity generally in the mangroves of China. Nevertheless, it is a relief that a slightly higher level of genetic diversity was observed in inter-population comparisons, especially in species with wide distribution ranges. The lack of genetic variation in populations of mangroves around the South China Sea has been found in *Sonneratia ovata*, *S. alba*, *Rhizophora apiculata*, *Ceriops tagal*, *Lumnitzera racemosa*, *Kandelia obovata,* and *Nypa fruticans* [33–41]. This phenomenon was considered to be attributable to repeated historical sea-level fluctuations [42]. On the other hand, low genetic diversity is expected in introduced non-native species (*Aegialitis annulate* and *Rhizophora mucronata*) due to the bottleneck effect during the process of introduction. A similar phenomenon is also expected in populations transplanted from low latitudinal regions to higher latitudinal regions in China (*Sonneratia alba*, *Kandelia obovata*, *Avicennia marina,* and *Aegiceras corniculatum*). Overall, we showed the potential of DNA barcoding to monitor the genetic composition of plant species at the whole community level, which may be informative for decision-making on conservation policies.

## Methods

### Sampling and sequencing

In each of the 33 sites, we collected all mangrove species and some common terrestrial species occurring near mangroves. For each species, three to six individuals which are at least ten meters apart were sampled, unless less than three individuals were found. We tried our best to collect branches with flowers and fruits for specimens. Leaves for DNA extraction were collected from the same individuals.

Total genomic DNA was isolated from leaf tissue dried in silica gel using a modified CTAB method [43]. DNA quality and quantity were examined by Nanodrop. The DNA was diluted to a final concentration of 50–100 ng/μL for polymerase chain reaction (PCR) amplification. Both published universal primers and newly designed primers were used in PCR amplification (Table S2).

The 20 μL PCR mixture contained 1 μL template DNA, 10 μL GenStar PCR StarMix, 1 μL each of the two primers, easy-Taq polymerase and ddH2O. PCR was performed on an engine of Applied Biosystem. The procedure started with an initial melting step at 94 °C for 4 min, followed by 30 cycles of 94 °C for 40s, annealing temperature for 45 s, and 72 °C for 1.30 min, and ended with a final elongation step at 36 °C for 10 min. For some specimens difficult to amplify, we used KOD polymerase instead. In these cases, the PCR mixture contained 1.2 μL template, 3 μL buffer, 3 μL dNTP, 0.9 μL of each forward and reverse primer, 0.6uL KOD polymerase, and 18.6 μL ddH2O. The procedure started with an initial melting step at 94 °C for 4 min, followed by 35 cycles of 98 °C for 10s and annealing temperature for 30s, and ended with a final elongation step at 68 °C for 1 min.

Amplicons were purified by 2% agarose gel electrophoresis. Then bidirectional sequencing reactions were performed on an ABI 3730 DNA analyzer (Guangzhou Tianyi Huiyuan Gene Technology Co., Ltd). The raw sequences were assembled and checked in person using Seqman in the LASERGENE software package (DNASTAR, Inc.).

### Sequence comparisons and phylogenetic analyses

The newly generated sequences were verified by searching in the GenBank using BLASTN and comparing with matched sequences of type materials if such sequence exists (Table S3). The sequences were aligned using MUSCLE in MEGA v7 [44] for each genomic region. The quality of the sequences was checked manually according to the principles proposed by Nilsson et al. [45]. GenBank accession numbers of these sequences are provided in the Table S4 of supplementary materials. The *trnH-psbA* region diverged a lot among different orders, which disabled a reliable alignment.

We calculated the sequence-pairwise genetic distances of each of the candidate barcodes following the Kimura 2-parameter model using the ‘Distance Calculations’ function in MEGA v7 [44]. The average intraspecific distances and average inter-species distances were then calculated. The Wilcoxon signed tests were performed to compare the divergences of inter-species distance among four single-marker barcodes followed by the Kress and Erickson test [23]. Intraspecific genetic distances were further determined for two classes, inter-population and within-population, according to whether the two sequences producing a pairwise genetic distance were from the same population. We also calculated the statistics of θ and π to measure genetic diversity for the species when more than two individuals had been collected.

The sequence comparison identifications were conducted by TaxonDNA v1.8 [22]. The ‘best match’ (BM) and ‘best close match’ (BCM) options were used in determining whether a query was matched correctly. In BM, the identification is (1) successful if the query and the closest matching sequence come from the same species; (2) ambiguous if closest matches are multiple sequences from different species; and (3) incorrect if the query matches with sequences from mismatched species names [22]. The BCM option used a 95% pairwise distance threshold [22]. A query without a barcode match below the threshold is considered unidentified. For the queries with barcode match below the threshold, the identification is assigned to correct, incorrect or ambiguous, according to the outlines described for BM above.

The NJ trees were generated in MEGA v7 based on the Kimura 2-parameter (K2P) genetic distance model [44]. The ML trees were constructed using the RAxML-8.2.9 program under the GTRCAT model [46]. Supporting rates were obtained from 1000 bootstrap replicates in NJ and ML methods.

The BI trees were constructed by MrBayes v. 3.27a [47], under GTR model with gamma-distributed rates across sites and a proportion of invariable sites (GTR + I + Γ). In Bayesian inferences, we initiated Markov Chain Monte Carlo (MCMC) simulations from random starting trees and ran for 1 × 10^6^ generations. Simulations were considered reaching convergence when the standard deviation was below 0.015. For markers that achieved no convergence when constructing BI tree for all samples, we constructed BI tree for each order by dividing the samples according to the orders. If a species itself represents one order, it was considered monophyletic.

Notably, the tree shown in Fig. 2 is not a phylogenetic tree constructed from real sequences, but a cladogram depicting the relationships of these species based on the APGIV system.

## Supplementary Information

**Additional file 1: Table S1.** Results of phylogenetic analysis based on NJ, ML, and BI. **Table S2.** Information of primers. **Table S3.** The availability of sequences of type material in GenBank. Table S4 GenBank accession number of DNA sequences newly gerenated in this study. **Figure S1.** Scatter plots of intraspecific genetic distance within and between populations of the ITS2 marker. Red dots indicate values of between populations and green dots indicate those of within populations. **Figure S2.** Scatter plots of intraspecific genetic distance within and between populations of the four genes. Red dots indicate values of between populations and green dots indicate those of within populations. **Figure S3.** Scatter plots of intraspecific genetic distance within and between populations of the four genes. Red dots indicate values of between populations and green dots indicate those of within populations. **Figure S4.** Scatter plots of intraspecific genetic distance within and between populations of the trnH-psbA marker. Red dots indicate values of between populations and green dots indicate those of within populations.

## Data Availability

The sequences of all barcodes were deposited in the Genome Sequence Archive in National Genomics Data Center, China National Center for Bioinformation (Accession number CRA004918). We also deposited the barcode sequences in Genbank and the accession numbers are provided in Table S4 of supplementary materials. The multiple sequence alignments and tree files in Newick format are deposited in Dryad (https://datadryad.org/stash/share/w61yRuoFMhtYWj3bNHQKOe3rmMesOfFF6LdHXFrURA4). All plant specimens were deposited in the herbarium of Sun Yat-sen University in Guangzhou, China.
