# Supplementary material for: Establishing community-wide DNA barcode references for conserving mangrove forests in China
Source: BMC Plant Biol. 2021 Dec 4;21:571. doi: 10.1186/s12870-021-03349-z (PMC8642986; doi:10.1186/s12870-021-03349-z)
Supplement: Supplementary file 1 — Additional file 1: Table S1. Results of phylogenetic analysis based on NJ, ML, and BI. Table S2. Information of primers. Table S3. The availability of sequences of type material in GenBank. Table S4 GenBank accession number of DNA sequences newly gerenated in this study. Figure S1. Scatter plots of intraspecific genetic distance within and between populations of the ITS2 marker. Red dots indicate values of between populations and green dots indicate those of within populations. Figure S2. Scatter plots of intraspecific genetic distance within and between populations of the four genes. Red dots indicate values of between populations and green dots indicate those of within populations. Figure S3. Scatter plots of intraspecific genetic distance within and between populations of the four genes. Red dots indicate values of between populations and green dots indicate those of within populations. Figure S4. Scatter plots of intraspecific genetic distance within and between populations of the trnH-psbA marker. Red dots indicate values of between populations and green dots indicate those of within populations. [file 12870_2021_3349_MOESM1_ESM.pdf]

Supplementary information for

Establishing community-wide DNA barcode references for conserving mangrove forests in China

Xiaomeng Mao[1], Wei Xie[1], Xinnian Li[1,2], Suhua Shi[1,*], Zixiao Guo[1,*]

[1]State Key Laboratory of Biocontrol, Guangdong Key Lab of Plant Resources, Southern Marine Science and Engineering Guangdong Laboratory (Zhuhai), School of Life Sciences, Sun Yat-sen University, Guangzhou, 510275, China

[2]Forevergen Biosciences Center, Guangzhou, China

*corresponding author: Zixiao Guo (guozx8@mail.sysu.edu.cn); Suhua Shi (lssssh@mail.sysu.edu.cn).

This file contains Figures S1-4 and Tables S1-4.

Table S1 Results of phylogenetic analysis based on NJ, ML, and BI

Species	I			R			M			T			I+R			IM			IT			RM			RT			MT			IRM			IRT			IMT			RMT			IRMT		
	NJ	ML	BI	NJ	ML	BI	NJ	ML	BI	NJ	ML	BI	NJ	ML	BI	NJ	ML	BI	NJ	ML	BI	NJ	ML	BI	NJ	ML	BI	NJ	ML	BI	NJ	ML	BI	NJ	ML	BI	NJ	ML	BI	NJ	ML	BI	NJ	ML	BI
Avicennia germinans	1	1	1	1	1	1	1	1	1	1	1	1	1	1	1	1	1	1	1	1	1	1	1	1	1	1	1	1	1	1	1	1	1	1	1	1	1	1	1	1	1	1	1	1	1
Avicennia marina	1	1	1	1	1	1	1	1	1	1	1	1	1	1	1	1	1	1	1	1	1	1	1	1	1	1	1	1	1	1	1	1	1	1	1	1	1	1	1	1	1	1	1	1	1
Acanthus ebracteatus	1	1	0	0	0	0	0	0	0	1	0	0	1	1	1	1	0	0	1	0	0	0	0	0	0	1	0	0	0	0	1	0	0	1	0	0	0	0	0	0	0	0	1	0	0
Acanthus ilicifolius	1	1	1	0	0	0	0	0	0	1	1	0	1	1	1	1	1	1	1	1	1	0	0	0	1	1	0	1	1	0	1	1	1	1	1	1	1	1	1	1	1	0	1	1	1
Dolichandrone spathacea				1	1	1	1	1	1	1	1	1										1	1	1	1	1	1	1	1	1										1	1	1			
Clerodendrum inerme	1	1	1	1	1	1	1	1	1	0	1	1	1	1	1	1	1	1	1	1	1	1	1	1	1	1	1	1	1	1	1	1	1	1	1	1	1	1	1	1	1	1	1	1	1
Premna obtusifolia	1	1	1	1	1	1	1	1	1	1	1	1	1	1	1	1	1	1	1	1	1	1	1	1	1	1	1	1	1	1	1	1	1	1	1	1	1	1	1	1	1	1	1	1	1
Stachytarpheta jamaicensis				1	1	1	1	1	1	1	1	1										1	1	1	1	1	1	1	1	1										1	1	1			
Myoporum_bontioides	1	1	1	1	1	1	1	1	1	1	1	1	1	1	1	1	1	1	1	1	1	1	1	1	1	1	1	1	1	1	1	1	1	1	1	1	1	1	1	1	1	1	1	1	1
Phyla nodiflora	1	1	1	1	1	1	1	1	1	1	1	1	1	1	1	1	1	1	1	1	1	1	1	1	1	1	1	1	1	1	1	1	1	1	1	1	1	1	1	1	1	1	1	1	1
Tephrosia purpurea				1	1	1				1	1	1													1	1	1																		
Pongamia pinnata	1	1	1	1	1	1	1	1	1	1	1	1	1	1	1	1	1	1	1	1	1	1	1	1	1	1	1	1	1	1	1	1	1	1	1	1	1	1	1	1	1	1	1	1	1
Sesbania cannabina	1	1	1	1	1	1				1	1	1	1	1	1				1	1	1				1	1	1							1	1	1									
Derris trifoliata	1	1	1	1	1	1	1	1	1	1	1	1	1	1	1	1	1	1	1	1	1	1	1	1	1	1	1	1	1	1	1	1	1	1	1	1	1	1	1	1	1	1	1	1	1
Crotalaria pallida	-1	-1	-1	-1	-1	-1	-1	-1	-1				-1	-1	-1	-1	-1	-1				-1	-1	-1							-1	-1	-1												
Caesalpinia bonduc	1	1	1	1	1	1	-1	-1	-1				1	1	1	-1	-1	-1				-1	-1	-1							-1	-1	-1												
Dalbergia tonkinensis	-1	-1	-1	-1	-1	-1	-1	-1	-1				-1	-1	-1	-1	-1	-1				-1	-1	-1							-1	-1	-1												
Canavalia maritima	1	1	1	1	1	1	1	1	1	1	1	1	1	1	1	1	1	1	1	1	1	1	1	1	1	1	1	1	1	1	1	1	1	1	1	1	1	1	1	1	1	1	1	1	1
Leucaena leucocephala				-1	-1	-1				-1	-1	-1													-1	1	-1																		
Aegiceras corniculatum	1	1	1	1	1	1	1	1	1	1	1	11	1	1	1	1	1	1	1	1	11	1	1	1	1	1	11	1	1	11	1	1	1	1	1	11	1	1	11	1	1	11	1	1	11
Barringtonia racemosa	1	1	1	1	1	1	1	1	1				1	1	1	1	1	1				1	1	1							1	1	1												
Cyperus malaccensis	1	1	1	1	1	1	-1	-1	-11	1	1	1	1	1	1	-1	-1	-11	1	1	1	-1	-1	-11	1	1	1				-1	-1	-11	1	1	1									
Scirpus mariqueter	1	1	1	1	1	1	-1	-1	-11	1	1	1	1	1	1	-1	-1	-11	1	1	1	-1	-1	-11	1	1	1	-1	-1	-11	-1	-1	-11	1	1	1	-1	-1	-11	-1	-1	-11	-1	-1	-11
Sedum lineare	1	1	11	1	1	11				1	1	11	1	1	11				1	1	11				1	1	11							1	1										
Excoecaria agallocha	1	1	1	1	1	1	1	1	1	1	1	1	1	1	1	1	1	1	1	1	1	1	1	1	1	1	1	1	1	1	1	1	1	1	1	1	1	1	1	1	1	1	1	1	1
Rhizophora apiculata	1	1	1	0	0	0	0	0	0	1	1	1	1	1	1	1	1	1	1	1	1	0	0	0	1	1	1				0	1	1	1	1	1									
Rhizophora mangle	-1	-1	-1	0	0	0	-1	-1	0	-1	-1	-1	-1	-1	-1	-1	-1	-1	-1	-1	-1	-1	-1	-1	-1	-1	-1	-1	-1	-1	-1	-1	-1	-1	-1	-1	-1	-1	-1	-1	-1	-1	-1	-1	-1
Rhizophora mucronata	1	1	1	0	0	0	0	0	0				1	1	1	1	1	1				0	0	0							0	1	1												
Rhizophora stylosa	0	0	0	0	0	0	0	0	0	1	1	1	0	0	1	0	1	1	1	1	1	1	0	0	1	1	1	1	1	1	0	0	1	1	1	1	1	1	1	1	1	1	1	1	1
Ceriops tagal	1	1	1	1	1	1	1	1	1	1	1	1	1	1	1	1	1	1	1	1	1	1	1	1	1	1	1	1	1	1	1	1	1	1	1	1	1	1	1	1	1	1	1	1	1
Bruguiera gymnorhiza	0	0	0	0	0	0	0	0	0	0	0	1	0	0	0	0	0	0	0	0	1	0	0	0	0	0	1	0	0	1	0	0	0	0	0	1	0	0	0	0	0	1	0	0	1
Bruguiera sexangula	0	0	0	0	0	0	0	0	0	0	1	1	0	0	0	0	0	0	1	1	0	0	0	0	1	1	0	1	1	0	0	0	0	1	1	0	1	1	0	1	1	0	1	1	0
Kandelia obovata	1	1	1	1	1	1	1	1	1	1	1	1	1	1	1	1	1	1	1	1	1	1	1	1	1	1	1	1	1	1	1	1	1	1	1	1	1	1	1	1	1	1	1	1	1
Abutilon indicum	1	1	1	1	1	1				1	1	1	1	1	1				1	1	1				1	1	1							1	1	1									
Sida acuta	1	1	1	1	1	1				-1	-1	-1	1	1	1				-1	-1	-1				-1	-1	-1							-1	-1	-1									
Heritiera littoralis	1	1	1	1	1	1	1	1	1	1	1	1	1	1	1	1	1	1	1	1	1	1	1	1	1	1	1	1	1	1	1	1	1	1	1	1	1	1	1	1	1	1	1	1	1
Waltheria indica	1	1	1	1	1	1	1	1	1	1	1	1	1	1	1	1	1	1	1	1	1	1	1	1	1	1	1	1	1	1	1	1	1	1	1	1	1	1	1	1	1	1	1	1	1
Thespesia populnea	1	1	1	1	1	1	1	1	1	1	1	1	1	1	1	1	1	1	1	1	1	1	1	1	1	1	1				1	1	1	1	1	1									
Hibiscus tiliaceus	0	0	0	0	0	0	0	0	0	0	0	0	0	0	0	0	0	0	1	1	0	0	0	0	0	0	0	0	0	0	0	0	0	0	0	0	0	0	0	0	0	0	0	0	0

Hibiscus hamabo	1	1	1	0	0	0	0	1	0	0	0	0	1	1	1	1	1	0	0	0	1	0	0	0	0	0	0	0	0	0	0	1	1	1	1	1	1	1	1	0	0	0	1	1	1
Scaevola hainanensis	1	1	1	1	1	1	1	1	1	0	0	0	1	1	1	1	1	1	1	1	0	1	1	1	0	0	1	1	1	1	1	1	1	1	1	1	1	1	1	1	1	1	1	1	1
Scaevola sericea	1	1	1	1	1	1	0	1	1	0	0	0	1	1	1	1	1	1	1	1	0	1	1	1	0	1	1	0	0	0	1	1	1	1	1	1	1	1	1	1	1	1	1	1	1
Pluchea indica	1	1	1	1	1	1	1	1	1	1	1	1	1	1	1	1	1	1	1	1	0	1	1	1	1	1	1	1	1	1	1	1	1	1	1	1	1	1	1	1	1	1	1	1	1
Hoya carnosa	1	1	1	1	1	1	1	1	1	1	1	1	1	1	1	1	1	1	1	1	1	1	1	1	1	1	1	1	1	1	1	1	1	1	1	1	1	1	1	1	1	1	1	1	1
Cerbera manghas	1	1	1	1	1	1	1	1	1	1	1	1	1	1	1	1	1	1	1	1	1	1	1	1	1	1	1	1	1	1	1	1	1	1	1	1	1	1	1	1	1	1	1	1	1
Morinda citrifolia				-1	-1	-1	-1	-1	-1	-1	-1	-1										-1	-1	-1	-1	-1	-1	-1	-1	-1										-1	-1	-1			
Scyphiphora hydrophyllacea	-1	-1	-1	-1	-1	-1	-1	-1	-1	-1	-1	-1	-1	-1	-1	-1	-1	-1	-1	-1	-1	-1	-1	-1	-1	-1	-1	-1	-1	-1	-1	-1	-1	-1	-1	-1	-1	-1	-1	-1	-1	-1	-1	-1	-1
Pandanus tectorius				1	1	11	1	1	11													1	1	11																					
Trema tomentosa	-1	-1	-11	-1	-1	-11				-1	-1	-11	-1	-1	-11				-1	-1	-11				-1	-1	-11							-1	-1										
Ipomoea pes-caprae	1	1	11	1	1	11	1	1	11	1	1	11	1	1	11	1	1	11	1	1	11	1	1	11	1	1	11	1	1	11	1	1	11	1	1	11	1	1	11	1	1	11	1	1	11
Casuarina equisetifolia	1	1	11	1	1	11	1	1	11	1	1	11	1	1	11	1	1	11	1	1	11	-1	1	11	1	1	11	1	1	11	1	1	11	1	1	11	1	1	11	1	1	11	1	1	11
Aegialitis annulata	1	1	1	1	1	1	1	1	1				1	1	1	1	1	1				1	1	1							1	1	1												
Sesuvium portulacastrum	1	1	1	1	1	1	1	1	1	0	1	1	1	1	1	1	1	1	1	1	1	1	1	1	1	1	1	1	1	1	1	1	1	1	1	1	1	1	1	1	1	1	1	1	1
Limonium sinensis	1	1	1	1	1	1	1	1	1	1	1	1	1	1	1	1	1	1	1	1	1	1	1	1	1	1	1	1	1	1	1	1	1	1	1	1	1	1	1	1	1	1	1	1	1
Portulaca pilosa	-1	-1	-1	-1	-1	-1	-1	-1	-1	-1	-1	-1	-1	-1	-1	-1	-1	-1	-1	-1	-1	-1	-1	-1	-1	-1	-1	-1	-1	-1	-1	-1	-1	-1	-1	-1	-1	-1	-1	-1	-1	-1	1	-1	-1
Suaeda australis	1	1	1	1	1	1	1	1	1	1	1	1	1	1	1	1	1	1	1	1	1	1	1	1	1	1	1	1	1	1	1	1	1	1	1	1	1	1	1	1	1	1	1	1	1
Suaeda glauca	1	1	1	1	1	1	1	1	1	1	1	1	1	1	1	1	1	1	1	1	1	1	1	1	1	1	1	1	1	1	1	1	1	1	1	1	1	1	1	1	1	1	1	1	1
Acrostichum aureum	1	1	1	0	0	0	0	1	1	0	0	0	1	0	1	1	0	1	1	1	1	1	0	1	0	0	0	1	1	1	0	0	1	1	0	1	1	1	1	1	0	1	1	0	1
Acrostichum speciosum	1	1	1	0	0	0	-1	-1	-1	0	0	0	1	1	1	-1	0	-1	1	1	1	-1	0	-1	0	0	0	-1	-1	-1	-1	0	-1	1	1	1	-1	-1	-1	-1	0	-1	-1	0	-1
Laguncularia racemosa	1	1	1	1	1	1	1	1	1	1	1	1	1	1	1	1	1	1	1	1	1	1	1	1	1	1	1	1	1	1	1	1	1	1	1	1	1	1	1	1	1	1	1	1	1
Lumnitzera littorea	1	1	1	1	1	0	1	1	1	1	1	1	1	1	1	1	1	1	1	1	1	1	1	0	1	1	1	1	1	1	1	1	1	1	1	1	1	1	1	1	1	1	1	1	1
Lumnitzera racemosa	1	1	1	1	1	1	1	1	1	1	0	1	1	1	1	1	1	1	1	1	1	1	1	1	1	1	1	1	1	1	0	1	1	1	1	1	1	1	1	1	1	1	1	1	1
Pemphis acidula	1	1	1	1	1	1	-1	-1	-1	1	1	1	1	1	1	-1	-1	-1	1	1	1	-1	-1	-1	1	1	1	-1	-1	-1	-1	-1	-1	1	1	1	-1	-1	-1	-1	-1	-1	-1	-1	-1
Sonneratia alba	0	1	1	1	0	1	0	0	0	0	1	0	1	1	1	0	1	1	0	1	1	1	1	1	0	-1	-1	1	1	0	0	1	1	-1	-1	-1	1	1	1	-1	-1	-1	-1	-1	-1
Sonneratia apetala	1	1	1	0	0	0	1	1	1	0	0	0	1	1	1	1	1	1	1	1	1	1	1	1	0	0	0	0	0	1	0	1	1	1	1	1	1	1	1	0	0	1	1	1	1
Sonneratia caseolaris	1	1	1	0	0	0	0	0	0	0	0	0	1	1	1	1	0	1	0	0	0	1	1	1	1	1	1	0	0	0	0	1	1	1	1	1	0	0	0	1	1	1	1	1	1
Sonneratia ovata	1	1	1	0	0	0	0	0	0	1	1	1	1	1	1	1	1	1	1	1	1	0	1	0	1	1	1	1	1	1	0	1	1	1	1	1	1	1	1	1	1	1	1	1	1
Conocarpus erectus	1	1	1	1	1	1	-1	-1	-1	1	1	1	1	1	1	-1	-1	-1	1	1	1	-1	-1	-1	1	1	1	-1	-1	-1	-1	-1	-1	1	1	1	-1	-1	-1	-1	1	-1	-1	-1	-1
Cardiospermum halicacabum	1	1	1	1	1	1							1	1	1																														
Xylocarpus granatum	1	1	1	1	1	1	1	1	11				1	1	1	1	1	11				1	1	11							1	1	11												
Hernandia sonora	1	1	11	1	1	11	1	1	11	1	1	11	1	1	11	1	1	11	1	1	11	1	1	11	1	1	11	1	1	11	1	1	11	1	1	11	1	1	11	1	1	11	1	1	11
Nypa fruticans				1	1	11	1	1	11	1	1	11										1	1	11	1	1	11	1	1	11										1	1	11			

1: all conspecific individuals formed a single clade with a bootstrap value >50%. 0: failures in identification. -1: only one sequence represents this species but separates well from other species.
Blanks: missing data. 11/-11: not include in BI tree as only one species or one sequence in its order.

Table S2 Information of primers

Gene	Name of Primers	Sequences of Primer (5'-3')	Tm(°C)	References
ITS2	S2F	ATGCGATACTTGGTGTGAAT	55	Chen et al., 2010
	S3R	GACGCTTCTCCAGACTACAAT		
	3	TAGCTACTTCTTCGCAGC	55	Kress et al., 2007
	4	GGTCCAGTCCGCCCTGATGG		
rbcL	1F	ATGTCACCACAAACAGAAAC	55	Chen et al., 2010
	724R	TCGCATGTACCTGCAGTAGC		
	LJF	ACCGATTGACTTATTCTACTCCCGA	58	Designed in this study
	LJR	CATTTCTTCAGACTTGCC		
matK	390F	CGATCTATTCATTCAATATTTC	55	Chen et al., 2010
	1326R	TCTAGCACACGAAAGTCGAAGT		
	2.1f	CCTATCCATCTGGAAATCTTAG	55	Chen et al., 2010
	5r	GTTCTAGCACAAGAAAGTCG		
	4L	CTTCGCTACTGGGTGAAAGATG	58	Designed in this study
	1932R	CAGACCGGCTTACTAATGGG		
	MAL_F	CTCACAAGGGNAAGA	50	Designed in this study
	MAL_R	TGTTTACGAGCCRAA		
	maF_4	TGATTCAAACCCTCCGCTAC	47.5	Designed in this study
	maR_4	ATTCCGAACCTAACCTTT		
trnH-psbA	psbA F	GTTATGCATGAACGTAATGCTC	52	Chen et al., 2010
	trnH R	CGCGCATGGTGGATTCACAATCC		
	f	ACTGCCTTGATCCACTTGGC	55	Chen et al., 2010
	f	CGAAGCTCCATCTACAAATGG		

Table S3 the availability of sequences of type material in GenBank

Species	rbcL	ITS2	matK	trnH-psbA
Avicennia marina	√	√	√	√
Avicennia germinans	√	√	√	√
Bruguiera gymnorhiza	√	√	√	√
Bruguiera sexangula	√	√	√	√
Ceriops tagal	√	√	√	√
Rhizophora mangle	√	√	√	√
Rhizophora mucronata	√	√	√	0
Rhizophora stylosa	√	√	√	√
Rhizophora apiculata	√	√	√	√
Sonneratia alba	√	√	√	√
Sonneratia apetala	√	√	√	√
Sonneratia caseolaris	√	√	√	√
Sonneratia hainanensis	√	√	√	√
Sonneratia ovata	√	√	√	√
Kandelia obovato	√	√	0	√
Acanthus ebracteatus	√	√	√	√
Acanthus ilicifolius	√	√	√	√
Acrostichum aureum	√	0	√	√
Acrostichum speciosum	√	0	0	√
Aegialitis annulata	√	0	√	0
Aegiceras corniculatum	√	√	√	√
Conocarpus erectus	√	√	√	√
Laguncularia racemosa	√	√	√	√
Lumnitzera littorea	√	√	√	0
Lumnitzera racemosa	√	√	√	√
Nypa fruticans	√	√	√	√
Pemphis acidula	√	√	√	√
Scyphiphora hydrophyllacea	√	√	√	√
Xylocarpus granatum	√	√	√	√
Barringtonia racemosa	√	√	√	√
Cerbera manghas	√	√	√	√
Clerodendrum inerme	√	√	√	√
Dolichandrone spathacea	√	√	√	√
Excoecaria agallocha	√	√	√	√
Hernandia sonora	√	0	√	√
Heritiera littoralis	√	√	√	√
Hibiscus tiliaceus	√	0	√	√
Pluchea indica	√	√	√	√
Pongamia pinnata	0	√	√	0
Premna obtusifolia	√	0	0	0

Thespesia populnea	√	√	√	√
Abutilon indicum	√	√	√	√
Caesalpinia bonduc	√	√	√	√
Canavalia maritima	0	0	0	0
Cardiospermum halicacabum	√	√	√	0
Casuarina equisetifolia	√	√	√	√
Crotalaria pallida	√	√	√	√
Cyperus malaccensis	0	0	0	0
Dalbergia tonkinensis	√	√	√	√
Derris trifoliata	√	√	√	√
Hibiscus hamabo	√	√	√	√
Hoya carnosa	√	√	√	√
Ipomoea pes-caprae	√	√	√	√
Leucaena leucocephala	√	√	√	√
Limonium sinense	√	√	√	√
Morinda citrifolia	√	√	√	√
Myoporum bontioides	√	√	0	√
Pandanus tectorius	√	√	√	√
Phyla nodiflora	√	√	√	√
Portulaca pilosa	√	√	√	√
Scaevola hainanensis	0	√	0	0
Scaevola sericea	0	0	0	0
Scirpus mariqueter	0	0	0	0
Sedum lineare	√	√	0	√
Sesbania cannabina	√	√	√	√
Sesuvium portulacastrum	√	√	√	√
Sida acuta	√	√	√	√
Stachytarpheta jamaicensis	√	√	√	√
Suaeda australis	√	√	√	√
Suaeda glauca	√	√	√	√
Tephrosia purpurea	√	√	√	√
Trema tomentosa	√	√	√	√
Waltheria indica	√	√	√	√

Note: "√" indicates at least one sequence is available while "0" no sequence is available

Table S4 GenBank accession number of DNA sequences newly gerenated in this study

Order	Species	Specimen ID	rbcL	ITS2	trnH-psbA	matK
Malvales	Abutilon indicum	Abutilon_indicum_GDST_536	OK634407	OK637344	NA	NA
Malvales	Abutilon indicum	Abutilon_indicum_HNDZ_202	OK634408	OK637345	OK636422	NA
Malvales	Abutilon indicum	Abutilon_indicum_HNDZ_203	OK634409	OK637346	OK636423	NA
Malvales	Abutilon indicum	Abutilon_indicum_HNHK_201	OK634410	OK637347	OK636424	NA
Malvales	Abutilon indicum	Abutilon_indicum_HNHK_204	OK634411	OK637348	OK636425	NA
Malvales	Abutilon indicum	Abutilon_indicum_HNHK_205	OK634412	OK637349	OK636426	NA
Lamiales	Acanthus ebracteatus	Acanthus_ebracteatus_GXZZ_746	OK634413	NA	OK636432	OL333883
Lamiales	Acanthus ebracteatus	Acanthus_ebracteatus_GXZZ_747	OK634414	NA	OK636433	OL333884
Lamiales	Acanthus ebracteatus	Acanthus_ebracteatus_GXZZ_748	OK634415	NA	OK636434	OL333885
Lamiales	Acanthus ebracteatus	Acanthus_ebracteatus_HNHK_136	OK634416	NA	OK636437	OL333886
Lamiales	Acanthus ebracteatus	Acanthus_ebracteatus_HNHK_137	OK634417	OK637352	OK636438	OL333887
Lamiales	Acanthus ebracteatus	Acanthus_ebracteatus_FJQZ_289	OK634418	OK637356	NA	OL333888
Lamiales	Acanthus ebracteatus	Acanthus_ebracteatus_FJQZ_290	OK634419	OK637357	NA	OL333889
Lamiales	Acanthus ebracteatus	Acanthus_ebracteatus_FJQZ_291	OK634420	OK637358	OK636427	OL333890
Lamiales	Acanthus ebracteatus	Acanthus_ebracteatus_GDST_556	OK634421	OK637359	OK636428	OL333891
Lamiales	Acanthus ebracteatus	Acanthus_ebracteatus_GDST_557	OK634422	OK637360	OK636429	OL333892
Lamiales	Acanthus ebracteatus	Acanthus_ebracteatus_GDST_558	OK634423	NA	OK636430	OL333893
Lamiales	Acanthus ebracteatus	Acanthus_ebracteatus_GDST_559	OK634424	OK637361	OK636431	OL333894
Lamiales	Acanthus ebracteatus	Acanthus_ebracteatus_HNHK_098	OK634444	OK637350	OK636435	OL333912
Lamiales	Acanthus ebracteatus	Acanthus_ebracteatus_HNHK_099	OK634445	OK637351	OK636436	OL333913
Lamiales	Acanthus ebracteatus	Acanthus_ebracteatus_HNWC_259	OK634446	OK637353	OK636439	OL333914
Lamiales	Acanthus ebracteatus	Acanthus_ebracteatus_HNWC_260	OK634447	OK637354	OK636440	OL333915
Lamiales	Acanthus ebracteatus	Acanthus_ebracteatus_HNWC_261	OK634448	OK637355	OK636441	OL333916
Lamiales	Acanthus ilicifolius	Acanthus_ilicifolius_GDSZ_962	OK634425	OK637362	OK636442	OL333895
Lamiales	Acanthus ilicifolius	Acanthus_ilicifolius_GDSZ_963	OK634426	OK637363	OK636443	NA
Lamiales	Acanthus ilicifolius	Acanthus_ilicifolius_GDSZ_964	OK634427	OK637364	OK636444	OL333896
Lamiales	Acanthus ilicifolius	Acanthus_ilicifolius_GDZH_677	OK634428	OK637365	OK636445	OL333897
Lamiales	Acanthus ilicifolius	Acanthus_ilicifolius_GDZH_678	OK634429	OK637366	OK636446	OL333898
Lamiales	Acanthus ilicifolius	Acanthus_ilicifolius_GDZH_679	OK634430	OK637367	OK636447	OL333899

Lamiales	Acanthus ilicifolius	Acanthus_ilicifolius_GDZH_680	OK634431	OK637368	OK636448	OL333900
Lamiales	Acanthus ilicifolius	Acanthus_ilicifolius_GDZJ_462	OK634432	NA	OK636449	OL333901
Lamiales	Acanthus ilicifolius	Acanthus_ilicifolius_GXLZ_896	OK634433	OK637369	OK636450	OL333902
Lamiales	Acanthus ilicifolius	Acanthus_ilicifolius_GXLZ_906	OK634434	OK637370	OK636451	OL333903
Lamiales	Acanthus ilicifolius	Acanthus_ilicifolius_GXMW_802	OK634435	OK637371	OK636452	OL333904
Lamiales	Acanthus ilicifolius	Acanthus_ilicifolius_GXMW_803	OK634436	OK637372	OK636453	NA
Lamiales	Acanthus ilicifolius	Acanthus_ilicifolius_GXMW_804	OK634437	OK637373	OK636454	OL333905
Lamiales	Acanthus ilicifolius	Acanthus_ilicifolius_GXTS_861	OK634438	OK637374	OK636455	OL333906
Lamiales	Acanthus ilicifolius	Acanthus_ilicifolius_GXTS_862	OK634439	OK637375	OK636456	OL333907
Lamiales	Acanthus ilicifolius	Acanthus_ilicifolius_GXTS_863	OK634440	OK637376	OK636457	OL333908
Lamiales	Acanthus ilicifolius	Acanthus_ilicifolius_GXZZ_764	OK634441	OK637377	OK636458	OL333909
Lamiales	Acanthus ilicifolius	Acanthus_ilicifolius_GXZZ_765	OK634442	OK637378	OK636459	OL333910
Lamiales	Acanthus ilicifolius	Acanthus_ilicifolius_GXZZ_766	OK634443	OK637379	OK636460	OL333911
Polypodiales	Acrostichum aureum	Acrostichum_aureum_GDST_537	OK634449	NA	OK636461	NA
Polypodiales	Acrostichum aureum	Acrostichum_aureum_GDST_538	OK634450	NA	OK636462	NA
Polypodiales	Acrostichum aureum	Acrostichum_aureum_GDST_539	OK634451	NA	OK636463	NA
Polypodiales	Acrostichum aureum	Acrostichum_aureum_HNDZ_197	OK634452	OK637381	OK636489	OL333918
Polypodiales	Acrostichum aureum	Acrostichum_aureum_GDST_540	OK634453	NA	OK636464	NA
Polypodiales	Acrostichum aureum	Acrostichum_aureum_GDZJ_441	OK634454	NA	OK636467	NA
Polypodiales	Acrostichum aureum	Acrostichum_aureum_GDZJ_442	OK634455	NA	OK636468	NA
Polypodiales	Acrostichum aureum	Acrostichum_aureum_GDZJ_443	OK634456	NA	OK636469	NA
Polypodiales	Acrostichum aureum	Acrostichum_aureum_GDZJ_444	OK634457	NA	OK636470	NA
Polypodiales	Acrostichum aureum	Acrostichum_aureum_GDZJ_445	OK634458	NA	OK636471	NA
Polypodiales	Acrostichum aureum	Acrostichum_aureum_GDZJ_446	OK634459	NA	OK636472	NA
Polypodiales	Acrostichum aureum	Acrostichum_aureum_GDZJ_447	OK634460	NA	OK636473	NA
Polypodiales	Acrostichum aureum	Acrostichum_aureum_GDZJ_484	OK634461	NA	OK636474	NA
Polypodiales	Acrostichum aureum	Acrostichum_aureum_GDZJ_485	OK634462	NA	OK636475	NA
Polypodiales	Acrostichum aureum	Acrostichum_aureum_GDZJ_486	OK634463	NA	OK636476	NA
Polypodiales	Acrostichum aureum	Acrostichum_aureum_GDZJ_487	OK634464	NA	NA	NA
Polypodiales	Acrostichum aureum	Acrostichum_aureum_GXLZ_880	OK634465	NA	OK636477	NA
Polypodiales	Acrostichum aureum	Acrostichum_aureum_GXLZ_881	OK634466	NA	OK636478	NA
Polypodiales	Acrostichum aureum	Acrostichum_aureum_GXMW_771	OK634467	NA	OK636480	NA

Polypodiales	Acrostichum aureum	Acrostichum_aureum_GXMW_772	OK634468	NA	OK636481	NA
Polypodiales	Acrostichum aureum	Acrostichum_aureum_GXMW_773	OK634469	NA	NA	NA
Polypodiales	Acrostichum aureum	Acrostichum_aureum_GXTS_821	OK634470	NA	OK636482	NA
Polypodiales	Acrostichum aureum	Acrostichum_aureum_GXTS_822	OK634471	NA	OK636483	NA
Polypodiales	Acrostichum aureum	Acrostichum_aureum_GXTS_823	OK634472	NA	OK636484	NA
Polypodiales	Acrostichum aureum	Acrostichum_aureum_GXZZ_758	OK634473	NA	OK636485	NA
Polypodiales	Acrostichum aureum	Acrostichum_aureum_GXZZ_759	OK634474	NA	OK636486	NA
Polypodiales	Acrostichum aureum	Acrostichum_aureum_GXZZ_760	OK634475	NA	OK636487	NA
Polypodiales	Acrostichum aureum	Acrostichum_aureum_HNDZ_195	OK634476	OK637380	OK636488	OL333917
Polypodiales	Acrostichum aureum	Acrostichum_aureum_HNHK_101	OK634477	OK637382	OK636490	OL333919
Polypodiales	Acrostichum aureum	Acrostichum_aureum_HNHK_102	OK634478	OK637383	OK636491	OL333920
Polypodiales	Acrostichum aureum	Acrostichum_aureum_HNHK_103	OK634479	NA	OK636492	OL333921
Polypodiales	Acrostichum aureum	Acrostichum_aureum_HNSY_368	OK634480	NA	NA	OL333922
Polypodiales	Acrostichum aureum	Acrostichum_aureum_GDSZ_965	NA	NA	OK636465	NA
Polypodiales	Acrostichum aureum	Acrostichum_aureum_GDSZ_966	NA	NA	OK636466	NA
Polypodiales	Acrostichum aureum	Acrostichum_aureum_GXLZ_932	NA	NA	OK636479	NA
Polypodiales	Acrostichum speciosum	Acrostichum_speciosum_GDZH_654	OK634481	NA	OK636493	NA
Polypodiales	Acrostichum speciosum	Acrostichum_speciosum_GDZH_655	OK634482	NA	OK636494	NA
Polypodiales	Acrostichum speciosum	Acrostichum_speciosum_GDZH_656	OK634483	NA	OK636495	NA
Polypodiales	Acrostichum speciosum	Acrostichum_speciosum_HNHK_088	OK634484	OK637384	OK636496	OL333923
Polypodiales	Acrostichum speciosum	Acrostichum_speciosum_HNHK_089	OK634485	OK637385	OK636497	NA
Caryophyllales	Aegialitis annulata	Aegialitis_annulata_HNHK_049	OK634486	OK637386	NA	OL333924
Caryophyllales	Aegialitis annulata	Aegialitis_annulata_HNHK_050	OK634487	OK637387	NA	OL333925
Ericales	Aegiceras corniculatum	Aegiceras_corniculatum_FJLH_282	OK634488	OK637388	NA	OL333926
Ericales	Aegiceras corniculatum	Aegiceras_corniculatum_FJLH_283	OK634489	OK637389	NA	OL333927
Ericales	Aegiceras corniculatum	Aegiceras_corniculatum_FJLH_284	OK634490	OK637390	NA	OL333928
Ericales	Aegiceras corniculatum	Aegiceras_corniculatum_FJLH_285	OK634491	OK637391	NA	OL333929
Ericales	Aegiceras corniculatum	Aegiceras_corniculatum_FJQZ_295	OK634492	OK637392	OK636498	OL333930
Ericales	Aegiceras corniculatum	Aegiceras_corniculatum_FJQZ_296	OK634493	OK637393	OK636499	OL333931
Ericales	Aegiceras corniculatum	Aegiceras_corniculatum_FJQZ_297	OK634494	OK637394	OK636500	OL333932
Ericales	Aegiceras corniculatum	Aegiceras_corniculatum_FJXM_308	OK634495	OK637395	OK636501	OL333933
Ericales	Aegiceras corniculatum	Aegiceras_corniculatum_FJXM_309	OK634496	OK637396	OK636502	OL333934

Ericales	Aegiceras corniculatum	Aegiceras_corniculatum_FJXM_310	OK634497	OK637397	OK636503	OL333935
Ericales	Aegiceras corniculatum	Aegiceras_corniculatum_FJXM_321	OK634498	OK637398	OK636504	OL333936
Ericales	Aegiceras corniculatum	Aegiceras_corniculatum_FJXM_322	OK634499	OK637399	OK636505	OL333937
Ericales	Aegiceras corniculatum	Aegiceras_corniculatum_FJXM_323	OK634500	OK637400	OK636506	OL333938
Ericales	Aegiceras corniculatum	Aegiceras_corniculatum_FJXM_324	OK634501	OK637401	OK636507	OL333939
Ericales	Aegiceras corniculatum	Aegiceras_corniculatum_GDSZ_981	OK634502	OK637402	NA	NA
Ericales	Aegiceras corniculatum	Aegiceras_corniculatum_GDSZ_982	OK634503	OK637403	NA	NA
Ericales	Aegiceras corniculatum	Aegiceras_corniculatum_GDSZ_983	OK634504	OK637404	NA	NA
Ericales	Aegiceras corniculatum	Aegiceras_corniculatum_GDZH_638	OK634505	OK637405	OK636508	OL333940
Ericales	Aegiceras corniculatum	Aegiceras_corniculatum_GDZH_639	OK634506	OK637406	OK636509	OL333941
Ericales	Aegiceras corniculatum	Aegiceras_corniculatum_GDZH_640	OK634507	OK637407	OK636510	OL333942
Ericales	Aegiceras corniculatum	Aegiceras_corniculatum_GDZJ_459	OK634508	OK637408	OK636511	OL333943
Ericales	Aegiceras corniculatum	Aegiceras_corniculatum_GDZJ_460	OK634509	OK637409	OK636512	OL333944
Ericales	Aegiceras corniculatum	Aegiceras_corniculatum_GDZJ_461	OK634510	OK637410	OK636513	OL333945
Ericales	Aegiceras corniculatum	Aegiceras_corniculatum_GDZJ_492	OK634511	OK637411	OK636514	OL333946
Ericales	Aegiceras corniculatum	Aegiceras_corniculatum_GDZJ_493	OK634512	OK637412	OK636515	OL333947
Ericales	Aegiceras corniculatum	Aegiceras_corniculatum_GDZJ_494	OK634513	OK637413	OK636516	OL333948
Ericales	Aegiceras corniculatum	Aegiceras_corniculatum_GDZJ_495	OK634514	OK637414	OK636517	OL333949
Ericales	Aegiceras corniculatum	Aegiceras_corniculatum_GXLZ_897	OK634515	OK637415	OK636518	OL333950
Ericales	Aegiceras corniculatum	Aegiceras_corniculatum_GXLZ_898	OK634516	OK637416	OK636519	OL333951
Ericales	Aegiceras corniculatum	Aegiceras_corniculatum_GXLZ_899	OK634517	OK637417	OK636520	OL333952
Ericales	Aegiceras corniculatum	Aegiceras_corniculatum_GXMW_774	OK634518	OK637418	OK636521	OL333953
Ericales	Aegiceras corniculatum	Aegiceras_corniculatum_GXMW_775	OK634519	OK637419	NA	OL333954
Ericales	Aegiceras corniculatum	Aegiceras_corniculatum_GXMW_776	OK634520	OK637420	OK636522	OL333955
Ericales	Aegiceras corniculatum	Aegiceras_corniculatum_GXTS_858	OK634521	OK637421	OK636523	OL333956
Ericales	Aegiceras corniculatum	Aegiceras_corniculatum_GXTS_859	OK634522	OK637422	OK636524	OL333957
Ericales	Aegiceras corniculatum	Aegiceras_corniculatum_GXTS_860	OK634523	OK637423	OK636525	OL333958
Ericales	Aegiceras corniculatum	Aegiceras_corniculatum_GXZZ_717	OK634524	OK637424	OK636526	OL333959
Ericales	Aegiceras corniculatum	Aegiceras_corniculatum_GXZZ_718	OK634525	OK637425	OK636527	OL333960
Ericales	Aegiceras corniculatum	Aegiceras_corniculatum_GXZZ_719	OK634526	OK637426	OK636528	OL333961
Ericales	Aegiceras corniculatum	Aegiceras_corniculatum_HNDZ_212	OK634527	OK637427	OK636529	OL333962
Ericales	Aegiceras corniculatum	Aegiceras_corniculatum_HNHK_132	OK634528	OK637428	OK636530	OL333963

Ericales	Aegiceras corniculatum	Aegiceras_corniculatum_HNHK_133	OK634529	OK637429	OK636531	OL333964
Ericales	Aegiceras corniculatum	Aegiceras_corniculatum_HNHK_134	OK634530	OK637430	OK636532	OL333965
Ericales	Aegiceras corniculatum	Aegiceras_corniculatum_HNSY_349	OK634531	OK637431	OK636533	OL333966
Ericales	Aegiceras corniculatum	Aegiceras_corniculatum_HNSY_379	OK634532	OK637432	OK636534	OL333967
Ericales	Aegiceras corniculatum	Aegiceras_corniculatum_HNSY_380	OK634533	OK637433	OK636535	OL333968
Ericales	Aegiceras corniculatum	Aegiceras_corniculatum_HNSY_381	OK634534	NA	OK636536	OL333969
Ericales	Aegiceras corniculatum	Aegiceras_corniculatum_HNWC_269	OK634535	OK637434	OK636537	OL333970
Ericales	Aegiceras corniculatum	Aegiceras_corniculatum_HNWC_270	OK634536	OK637435	OK636538	OL333971
Lamiales	Avicennia germinans	Avicennia_germinans_HNHK_109	OK634537	OK637436	OK636539	OL333972
Lamiales	Avicennia germinans	Avicennia_germinans_HNHK_110	OK634538	OK637437	OK636540	NA
Lamiales	Avicennia germinans	Avicennia_germinans_HNHK_111	OK634539	OK637438	OK636541	OL333973
Lamiales	Avicennia germinans	Avicennia_germinans_HNHK_112	OK634540	OK637439	OK636542	OL333974
Lamiales	Avicennia germinans	Avicennia_germinans_HNHK_113	OK634541	OK637440	OK636543	OL333975
Lamiales	Avicennia marina	Avicennia_marina_FJXM_271	OK634542	OK637441	OK636544	OL333977
Lamiales	Avicennia marina	Avicennia_marina_FJXM_272	OK634543	OK637442	OK636545	OL333979
Lamiales	Avicennia marina	Avicennia_marina_FJXM_273	OK634544	OK637443	OK636546	OL333980
Lamiales	Avicennia marina	Avicennia_marina_FJXM_274	OK634545	OK637444	OK636547	OL333981
Lamiales	Avicennia marina	Avicennia_marina_FJXM_298	OK634546	OK637445	OK636548	OL333982
Lamiales	Avicennia marina	Avicennia_marina_FJXM_299	OK634547	OK637446	OK636549	OL333983
Lamiales	Avicennia marina	Avicennia_marina_FJXM_300	OK634548	OK637447	OK636550	OL333984
Lamiales	Avicennia marina	Avicennia_marina_FJXM_325	OK634549	OK637448	OK636551	OL333985
Lamiales	Avicennia marina	Avicennia_marina_FJXM_326	OK634550	OK637449	OK636552	OL333986
Lamiales	Avicennia marina	Avicennia_marina_FJXM_327	OK634551	OK637450	OK636553	OL333987
Lamiales	Avicennia marina	Avicennia_marina_FJXM_328	OK634552	OK637451	OK636554	OL333988
Lamiales	Avicennia marina	Avicennia_marina_GDSZ_938	OK634553	OK637452	OK636555	OL333989
Lamiales	Avicennia marina	Avicennia_marina_GDSZ_939	OK634554	OK637453	OK636556	OL333990
Lamiales	Avicennia marina	Avicennia_marina_GDSZ_940	OK634555	OK637454	OK636557	OL333991
Lamiales	Avicennia marina	Avicennia_marina_GDZJ_477	OK634556	OK637455	NA	OL333992
Lamiales	Avicennia marina	Avicennia_marina_GDZJ_478	OK634557	OK637456	OK636558	OL333993
Lamiales	Avicennia marina	Avicennia_marina_GDZJ_479	OK634558	OK637457	OK636559	OL333994
Lamiales	Avicennia marina	Avicennia_marina_GXMW_935	OK634559	OK637458	OK636560	OL333995
Lamiales	Avicennia marina	Avicennia_marina_GXMW_936	OK634560	OK637459	OK636561	OL333996

Lamiales	Avicennia marina	Avicennia_marina_GXMW_937	OK634561	OK637460	OK636562	OL333997
Lamiales	Avicennia marina	Avicennia_marina_GXTS_827	OK634562	OK637461	OK636563	OL333978
Lamiales	Avicennia marina	Avicennia_marina_GXTS_828	OK634563	OK637462	OK636564	OL333998
Lamiales	Avicennia marina	Avicennia_marina_GXTS_829	OK634564	OK637463	OK636565	OL333999
Lamiales	Avicennia marina	Avicennia_marina_GXTS_929	OK634565	OK637464	OK636566	OL334000
Lamiales	Avicennia marina	Avicennia_marina_GXTS_930	OK634566	OK637465	OK636567	OL334001
Lamiales	Avicennia marina	Avicennia_marina_GXTS_931	OK634567	OK637466	OK636568	OL334002
Lamiales	Avicennia marina	Avicennia_marina_GXZZ_713	OK634568	OK637467	OK636569	OL334003
Lamiales	Avicennia marina	Avicennia_marina_GXZZ_714	OK634569	OK637468	OK636570	OL334004
Lamiales	Avicennia marina	Avicennia_marina_GXZZ_715	OK634570	OK637469	OK636571	OL334005
Lamiales	Avicennia marina	Avicennia_marina_GXZZ_716	OK634571	OK637470	OK636572	OL334006
Lamiales	Avicennia marina	Avicennia_marina_HNDZ_151	OK634572	OK637471	OK636573	OL334007
Lamiales	Avicennia marina	Avicennia_marina_HNDZ_152	OK634573	OK637472	OK636574	OL334008
Lamiales	Avicennia marina	Avicennia_marina_HNDZ_153	OK634574	OK637473	OK636575	OL334009
Lamiales	Avicennia marina	Avicennia_marina_HNHK_042	OK634575	OK637474	OK636576	OL334010
Lamiales	Avicennia marina	Avicennia_marina_HNHK_043	OK634576	OK637475	OK636577	OL334011
Lamiales	Avicennia marina	Avicennia_marina_HNHK_054	OK634577	OK637476	OK636578	OL334012
Lamiales	Avicennia marina	Avicennia_marina_HNSY_329	OK634578	OK637477	OK636579	OL333976
Lamiales	Avicennia marina	Avicennia_marina_HNSY_330	OK634579	OK637478	OK636580	NA
Lamiales	Avicennia marina	Avicennia_marina_HNSY_359	OK634580	OK637479	OK636581	OL334013
Lamiales	Avicennia marina	Avicennia_marina_HNSY_360	OK634581	OK637480	OK636582	OL334014
Lamiales	Avicennia marina	Avicennia_marina_HNSY_361	OK634582	OK637481	OK636583	OL334015
Lamiales	Avicennia marina	Avicennia_marina_HNSY_387	OK634583	OK637482	OK636584	OL334016
Lamiales	Avicennia marina	Avicennia_marina_HNSY_388	OK634584	OK637483	OK636585	OL334017
Lamiales	Avicennia marina var. australasica	Avicennia_marina_australasica_HNHK_053	OK635271	OK638119	NA	NA
Lamiales	Avicennia marina var. eucalyptifolia	Avicennia_marina_eucalyptifolia_HNHK_051	OK635272	OK638117	NA	OL334504
Lamiales	Avicennia marina var. eucalyptifolia	Avicennia_marina_eucalyptifolia_HNHK_052	OK635273	OK638118	NA	OL334505
Ericales	Barringtonia racemosa	Barringtonia_racemosa_GDZH_644	OK634585	OK637484	NA	OL334018
Ericales	Barringtonia racemosa	Barringtonia_racemosa_GDZH_645	OK634586	OK637485	NA	OL334019
Ericales	Barringtonia racemosa	Barringtonia_racemosa_GDZH_646	OK634587	OK637486	NA	OL334020
Ericales	Barringtonia racemosa	Barringtonia_racemosa_HNHK_147	OK634588	OK637487	NA	OL334021
Ericales	Barringtonia racemosa	Barringtonia_racemosa_HNHK_148	OK634589	OK637488	NA	OL334022

Malpighiales	Bruguiera gymnorhiza	Bruguiera_gymnorhiza_GDSZ_972	OK634590	OK637489	NA	NA
Malpighiales	Bruguiera gymnorhiza	Bruguiera_gymnorhiza_GDSZ_973	OK634591	OK637490	NA	OL334023
Malpighiales	Bruguiera gymnorhiza	Bruguiera_gymnorhiza_GDSZ_974	OK634592	OK637491	NA	NA
Malpighiales	Bruguiera gymnorhiza	Bruguiera_gymnorhiza_GDZH_650	OK634593	OK637492	NA	OL334024
Malpighiales	Bruguiera gymnorhiza	Bruguiera_gymnorhiza_GDZH_651	OK634594	OK637493	NA	OL334025
Malpighiales	Bruguiera gymnorhiza	Bruguiera_gymnorhiza_GDZH_652	OK634595	OK637494	NA	OL334026
Malpighiales	Bruguiera gymnorhiza	Bruguiera_gymnorhiza_GDZH_657	OK634596	OK637495	NA	NA
Malpighiales	Bruguiera gymnorhiza	Bruguiera_gymnorhiza_GDZH_667	OK634597	OK637496	NA	OL334396
Malpighiales	Bruguiera gymnorhiza	Bruguiera_gymnorhiza_GDZH_668	OK634598	OK637497	NA	OL334397
Malpighiales	Bruguiera gymnorhiza	Bruguiera_gymnorhiza_GDZJ_498	OK634599	OK637498	NA	OL334027
Malpighiales	Bruguiera gymnorhiza	Bruguiera_gymnorhiza_GDZJ_499	OK634600	OK637499	NA	OL334028
Malpighiales	Bruguiera gymnorhiza	Bruguiera_gymnorhiza_GDZJ_500	OK634601	OK637500	NA	OL334029
Malpighiales	Bruguiera gymnorhiza	Bruguiera_gymnorhiza_GDZJ_501	OK634602	OK637501	NA	OL334030
Malpighiales	Bruguiera gymnorhiza	Bruguiera_gymnorhiza_GXTS_864	OK634603	OK637502	NA	OL334031
Malpighiales	Bruguiera gymnorhiza	Bruguiera_gymnorhiza_GXTS_865	OK634604	OK637503	NA	OL334032
Malpighiales	Bruguiera gymnorhiza	Bruguiera_gymnorhiza_GXTS_866	OK634605	OK637504	NA	OL334033
Malpighiales	Bruguiera gymnorhiza	Bruguiera_gymnorhiza_GXZZ_696	OK634606	OK637505	NA	OL334034
Malpighiales	Bruguiera gymnorhiza	Bruguiera_gymnorhiza_GXZZ_697	OK634607	OK637506	NA	OL334035
Malpighiales	Bruguiera gymnorhiza	Bruguiera_gymnorhiza_GXZZ_698	OK634608	OK637507	NA	OL334036
Malpighiales	Bruguiera gymnorhiza	Bruguiera_gymnorhiza_GXZZ_752	OK634609	OK637508	NA	OL334037
Malpighiales	Bruguiera gymnorhiza	Bruguiera_gymnorhiza_GXZZ_753	OK634610	OK637509	NA	OL334038
Malpighiales	Bruguiera gymnorhiza	Bruguiera_gymnorhiza_HNDZ_206	OK634611	OK637510	OK636586	OL334039
Malpighiales	Bruguiera gymnorhiza	Bruguiera_gymnorhiza_HNDZ_207	OK634612	OK637511	OK636587	OL334040
Malpighiales	Bruguiera gymnorhiza	Bruguiera_gymnorhiza_HNDZ_208	OK634613	OK637512	OK636588	OL334041
Malpighiales	Bruguiera gymnorhiza	Bruguiera_gymnorhiza_HNHK_118	OK634614	OK637513	NA	OL334042
Malpighiales	Bruguiera gymnorhiza	Bruguiera_gymnorhiza_HNHK_121	OK634615	OK637515	NA	OL334043
Malpighiales	Bruguiera gymnorhiza	Bruguiera_gymnorhiza_HNSY_374	OK634616	OK637516	OK636589	OL334044
Malpighiales	Bruguiera gymnorhiza	Bruguiera_gymnorhiza_HNSY_375	OK634617	OK637517	OK636590	OL334045
Malpighiales	Bruguiera gymnorhiza	Bruguiera_gymnorhiza_HNSY_376	OK634618	OK637518	NA	OL334046
Malpighiales	Bruguiera gymnorhiza	Bruguiera_gymnorhiza_HNSY_419	OK634619	OK637519	NA	OL334047
Malpighiales	Bruguiera gymnorhiza	Bruguiera_gymnorhiza_HNSY_420	OK634620	OK637520	OK636591	OL334048
Malpighiales	Bruguiera gymnorhiza	Bruguiera_gymnorhiza_HNSY_422	OK634621	OK637522	NA	OL334050

Malpighiales	Bruguiera gymnorhiza	Bruguiera_gymnorhiza_HNWC_244	OK634622	OK637523	NA	OL334051
Malpighiales	Bruguiera gymnorhiza	Bruguiera_gymnorhiza_HNWC_245	OK634623	OK637524	NA	OL334052
Malpighiales	Bruguiera gymnorhiza	Bruguiera_gymnorhiza_HNWC_247	OK634624	OK637525	NA	OL334053
Malpighiales	Bruguiera gymnorhiza	Bruguiera_gymnorhiza_HNWC_248	OK634625	OK637526	NA	OL334054
Malpighiales	Bruguiera sexangula	Bruguiera_sexangula_GDSZ_943	OK634627	OK637527	NA	NA
Malpighiales	Bruguiera sexangula	Bruguiera_sexangula_HNHK_065	OK634628	OK637528	OK636592	OL334055
Malpighiales	Bruguiera sexangula	Bruguiera_sexangula_HNHK_067	OK634629	OK637530	OK636594	OL334056
Malpighiales	Bruguiera sexangula	Bruguiera_sexangula_HNHK_068	OK634630	OK637531	OK636595	OL334057
Malpighiales	Bruguiera sexangula	Bruguiera_sexangula_HNHK_069	OK634631	OK637532	OK636596	OL334058
Malpighiales	Bruguiera sexangula	Bruguiera_sexangula_HNHK_086	OK634632	OK637533	NA	OL334059
Malpighiales	Bruguiera sexangula	Bruguiera_sexangula_HNSY_391	OK634633	OK637534	OK636597	OL334061
Malpighiales	Bruguiera sexangula	Bruguiera_sexangula_HNSY_392	OK634634	OK637535	OK636598	OL334062
Malpighiales	Bruguiera sexangula	Bruguiera_sexangula_HNSY_393	OK634635	OK637536	OK636599	OL334063
Malpighiales	Bruguiera sexangula	Bruguiera_sexangula_HNSY_394	OK634636	OK637537	OK636600	OL334064
Malpighiales	Bruguiera sexangula	Bruguiera_sexangula_HNWC_242	OK634637	OK637538	NA	NA
Malpighiales	Bruguiera sexangula	Bruguiera_sexangula_HNWC_249	OK634638	OK637539	NA	OL334065
Malpighiales	Bruguiera sexangula	Bruguiera_sexangula_HNWC_251	OK634639	OK637540	NA	OL334066
Malpighiales	Bruguiera sexangula	Bruguiera_sexangula_HNWC_263	OK634640	OK637541	NA	OL334067
Malpighiales	Bruguiera sexangula	Bruguiera_sexangula_HNHK_066	NA	OK637529	OK636593	NA
Malpighiales	Bruguiera x rhynchopetala	Bruguiera_gymnorhiza_sexangula_HNHK_087	OK635274	OK638128	NA	OL334060
Malpighiales	Bruguiera x rhynchopetala	Bruguiera_gymnorhiza_sexangula_HNSY_409	OK635275	OK638130	NA	OL334510
Malpighiales	Bruguiera x rhynchopetala	Bruguiera_gymnorhiza_sexangula_HNHK_119	OK635276	OK637514	OK637074	NA
Malpighiales	Bruguiera x rhynchopetala	Bruguiera_gymnorhiza_sexangula_HNHK_120	OK635277	OK638129	NA	NA
Malpighiales	Bruguiera x rhynchopetala	Bruguiera_gymnorhiza_sexangula_HNSY_421	OK635278	OK637521	OK637075	OL334049
Malpighiales	Bruguiera x rhynchopetala	Bruguiera_gymnorhiza_sexangula_HNWC_24(	OK635279	OK638131	NA	NA
Malpighiales	Bruguiera x rhynchopetala	Bruguiera_gymnorhiza_sexangula_HNWC_25(	OK635280	OK638132	NA	NA
Fabales	Caesalpinia bonduc	Caesalpinia_bonduc_HNSY_398	OK634641	OK637542	NA	OL334068
Fabales	Caesalpinia bonduc	Caesalpinia_bonduc_HNSY_399	OK634642	OK637543	NA	NA
Fabales	Canavalia maritima	Canavalia_maritima_GDST_523	OK634643	NA	OK636601	OL334069
Fabales	Canavalia maritima	Canavalia_maritima_GDST_524	OK634644	OK637544	OK636602	OL334070
Fabales	Canavalia maritima	Canavalia_maritima_GDST_525	OK634645	OK637545	OK636603	OL334071
Fabales	Canavalia maritima	Canavalia_maritima_GDST_541	OK634646	OK637546	OK636604	OL334072

Fabales	Canavalia maritima	Canavalia_maritima_GDST_542	OK634647	OK637547	OK636605	OL334073
Fabales	Canavalia maritima	Canavalia_maritima_GDST_543	OK634648	OK637548	OK636606	OL334074
Fabales	Canavalia maritima	Canavalia_maritima_GDSZ_941	OK634649	OK637549	OK636607	OL334088
Fabales	Canavalia maritima	Canavalia_maritima_GDSZ_942	OK634650	OK637550	OK636608	OL334075
Fabales	Canavalia maritima	Canavalia_maritima_GDZH_647	OK634651	OK637551	OK636609	OL334076
Fabales	Canavalia maritima	Canavalia_maritima_GDZH_648	OK634652	OK637552	OK636610	OL334082
Fabales	Canavalia maritima	Canavalia_maritima_GDZH_649	OK634653	OK637553	OK636611	OL334077
Fabales	Canavalia maritima	Canavalia_maritima_GDZJ_518	OK634654	OK637554	OK636612	NA
Fabales	Canavalia maritima	Canavalia_maritima_GXLZ_888	OK634655	OK637555	OK636613	OL334078
Fabales	Canavalia maritima	Canavalia_maritima_GXLZ_889	OK634656	OK637556	OK636614	OL334079
Fabales	Canavalia maritima	Canavalia_maritima_GXLZ_919	OK634657	OK637557	OK636615	OL334080
Fabales	Canavalia maritima	Canavalia_maritima_GXMW_768	OK634658	OK637558	OK636616	OL334081
Fabales	Canavalia maritima	Canavalia_maritima_GXMW_769	OK634659	OK637559	OK636617	OL334083
Fabales	Canavalia maritima	Canavalia_maritima_GXMW_770	OK634660	OK637560	NA	OL334086
Fabales	Canavalia maritima	Canavalia_maritima_GXTS_873	OK634661	OK637561	OK636618	OL334084
Fabales	Canavalia maritima	Canavalia_maritima_GXTS_874	OK634662	OK637562	OK636619	OL334085
Fabales	Canavalia maritima	Canavalia_maritima_GXTS_875	OK634663	OK637563	OK636620	OL334087
Fabales	Canavalia maritima	Canavalia_maritima_GXZZ_703	OK634664	OK637564	OK636621	OL334089
Fabales	Canavalia maritima	Canavalia_maritima_GXZZ_704	OK634665	OK637565	OK636622	OL334090
Fabales	Canavalia maritima	Canavalia_maritima_GXZZ_705	OK634666	OK637566	OK636623	OL334091
Sapindales	Cardiospermum halicacabum	Cardiospermum_halicacabum_HNHK_156	OK634667	OK637567	NA	NA
Sapindales	Cardiospermum halicacabum	Cardiospermum_halicacabum_HNHK_157	OK634668	OK637568	NA	NA
Fagales	Casuarina equisetifolia	Casuarina_equisetifolia_GDSZ_975	OK634669	OK637569	NA	OL334092
Fagales	Casuarina equisetifolia	Casuarina_equisetifolia_GDSZ_976	OK634670	OK637570	NA	OL334093
Fagales	Casuarina equisetifolia	Casuarina_equisetifolia_GDSZ_977	OK634671	OK637571	OK636624	OL334094
Fagales	Casuarina equisetifolia	Casuarina_equisetifolia_GXTS_848	OK634672	NA	OK636625	NA
Fagales	Casuarina equisetifolia	Casuarina_equisetifolia_GXTS_849	OK634673	OK637572	OK636626	OL334095
Fagales	Casuarina equisetifolia	Casuarina_equisetifolia_GXTS_850	OK634674	NA	OK636627	OL334096
Gentianales	Cerbera manghas	Cerbera_manghas_GDSZ_944	OK634675	OK637573	OK636628	OL334101
Gentianales	Cerbera manghas	Cerbera_manghas_GDSZ_945	OK634676	OK637574	OK636629	OL334102
Gentianales	Cerbera manghas	Cerbera_manghas_GDSZ_946	OK634677	OK637575	OK636630	OL334103
Gentianales	Cerbera manghas	Cerbera_manghas_GDZH_635	OK634678	OK637576	OK636631	OL334104

Gentianales	Cerbera manghas	Cerbera_manghas_GDZH_636	OK634679	OK637577	OK636632	OL334105
Gentianales	Cerbera manghas	Cerbera_manghas_GDZH_637	OK634680	OK637578	OK636633	OL334106
Gentianales	Cerbera manghas	Cerbera_manghas_GDZJ_472	OK634681	OK637579	OK636634	OL334097
Gentianales	Cerbera manghas	Cerbera_manghas_GDZJ_473	OK634682	OK637580	OK636635	OL334098
Gentianales	Cerbera manghas	Cerbera_manghas_GDZJ_474	OK634683	OK637581	OK636636	OL334099
Gentianales	Cerbera manghas	Cerbera_manghas_GXTS_867	OK634684	OK637582	OK636637	OL334107
Gentianales	Cerbera manghas	Cerbera_manghas_GXTS_868	OK634685	OK637583	OK636638	OL334108
Gentianales	Cerbera manghas	Cerbera_manghas_GXTS_869	OK634686	OK637584	NA	OL334109
Gentianales	Cerbera manghas	Cerbera_manghas_GXZZ_726	OK634687	OK637585	OK636639	OL334110
Gentianales	Cerbera manghas	Cerbera_manghas_GXZZ_727	OK634688	OK637586	OK636640	OL334111
Gentianales	Cerbera manghas	Cerbera_manghas_GXZZ_728	OK634689	OK637587	OK636641	OL334112
Gentianales	Cerbera manghas	Cerbera_manghas_HNHK_070	OK634690	OK637588	OK636642	OL334100
Malpighiales	Ceriops tagal	Ceriops_tagal_HNDZ_177	OK634691	OK637589	OK636644	OL334113
Malpighiales	Ceriops tagal	Ceriops_tagal_HNDZ_178	OK634692	OK637590	OK636645	OL334114
Malpighiales	Ceriops tagal	Ceriops_tagal_HNDZ_179	OK634693	OK637591	OK636646	OL334115
Malpighiales	Ceriops tagal	Ceriops_tagal_HNDZ_180	OK634694	OK637592	OK636647	OL334116
Malpighiales	Ceriops tagal	Ceriops_tagal_HNDZ_181	OK634695	OK637593	OK636648	OL334117
Malpighiales	Ceriops tagal	Ceriops_tagal_HNHK_090	OK634696	NA	OK636649	OL334118
Malpighiales	Ceriops tagal	Ceriops_tagal_HNHK_091	OK634697	OK637594	OK636650	OL334119
Malpighiales	Ceriops tagal	Ceriops_tagal_HNSY_339	OK634698	OK637595	OK636651	OL334120
Malpighiales	Ceriops tagal	Ceriops_tagal_HNSY_340	OK634699	OK637596	OK636652	OL334121
Malpighiales	Ceriops tagal	Ceriops_tagal_HNSY_341	OK634700	OK637597	OK636653	NA
Lamiales	Clerodendrum inerme	Clerodendrum_inerme_GDST_530	OK634701	OK637598	OK636654	NA
Lamiales	Clerodendrum inerme	Clerodendrum_inerme_GDST_531	OK634702	OK637599	OK636655	NA
Lamiales	Clerodendrum inerme	Clerodendrum_inerme_GDST_548	OK634703	OK637600	OK636656	NA
Lamiales	Clerodendrum inerme	Clerodendrum_inerme_GDST_549	OK634704	OK637601	OK636657	NA
Lamiales	Clerodendrum inerme	Clerodendrum_inerme_GDST_550	OK634705	OK637602	OK636658	NA
Lamiales	Clerodendrum inerme	Clerodendrum_inerme_GDST_551	OK634706	OK637603	OK636659	NA
Lamiales	Clerodendrum inerme	Clerodendrum_inerme_GDSZ_985	OK634707	OK637604	OK636660	NA
Lamiales	Clerodendrum inerme	Clerodendrum_inerme_GDSZ_986	OK634708	OK637605	NA	NA
Lamiales	Clerodendrum inerme	Clerodendrum_inerme_GDZH_631	OK634709	OK637607	OK636661	NA
Lamiales	Clerodendrum inerme	Clerodendrum_inerme_GDZJ_463	OK634710	OK637608	OK636662	OL334122

Lamiales	Clerodendrum inerme	Clerodendrum_inerme_GDZJ_464	OK634711	OK637609	OK636663	OL334123
Lamiales	Clerodendrum inerme	Clerodendrum_inerme_GDZJ_465	OK634712	OK637610	OK636664	OL334124
Lamiales	Clerodendrum inerme	Clerodendrum_inerme_GDZJ_480	OK634713	OK637611	OK636665	OL334125
Lamiales	Clerodendrum inerme	Clerodendrum_inerme_GDZJ_481	OK634714	OK637612	OK636666	OL334126
Lamiales	Clerodendrum inerme	Clerodendrum_inerme_GDZJ_482	OK634715	OK637613	OK636667	OL334127
Lamiales	Clerodendrum inerme	Clerodendrum_inerme_GDZJ_483	OK634716	OK637614	OK636668	OL334128
Lamiales	Clerodendrum inerme	Clerodendrum_inerme_GXLZ_885	OK634717	OK637615	OK636669	OL334129
Lamiales	Clerodendrum inerme	Clerodendrum_inerme_GXLZ_886	OK634718	OK637616	OK636670	NA
Lamiales	Clerodendrum inerme	Clerodendrum_inerme_GXLZ_887	OK634719	OK637617	OK636671	NA
Lamiales	Clerodendrum inerme	Clerodendrum_inerme_GXMW_782	OK634720	OK637618	OK636672	NA
Lamiales	Clerodendrum inerme	Clerodendrum_inerme_GXMW_783	OK634721	OK637619	OK636673	OL334130
Lamiales	Clerodendrum inerme	Clerodendrum_inerme_GXMW_784	OK634722	OK637620	OK636674	OL334131
Lamiales	Clerodendrum inerme	Clerodendrum_inerme_GXMW_798	OK634723	OK637621	OK636675	NA
Lamiales	Clerodendrum inerme	Clerodendrum_inerme_GXTS_808	OK634724	OK637622	OK636676	NA
Lamiales	Clerodendrum inerme	Clerodendrum_inerme_GXTS_809	OK634725	OK637623	OK636677	OL334132
Lamiales	Clerodendrum inerme	Clerodendrum_inerme_GXTS_810	OK634726	OK637624	OK636678	OL334133
Lamiales	Clerodendrum inerme	Clerodendrum_inerme_GXZZ_692	OK634727	OK637625	OK636679	OL334134
Lamiales	Clerodendrum inerme	Clerodendrum_inerme_GXZZ_693	OK634728	OK637626	OK636680	NA
Lamiales	Clerodendrum inerme	Clerodendrum_inerme_GXZZ_694	OK634729	OK637627	OK636681	OL334135
Lamiales	Clerodendrum inerme	Clerodendrum_inerme_GXZZ_695	OK634730	OK637628	OK636682	OL334136
Lamiales	Clerodendrum inerme	Clerodendrum_inerme_HNDZ_213	OK634731	OK637629	OK636683	OL334137
Lamiales	Clerodendrum inerme	Clerodendrum_inerme_HNDZ_214	OK634732	OK637630	OK636684	OL334138
Lamiales	Clerodendrum inerme	Clerodendrum_inerme_HNDZ_215	OK634733	OK637631	OK636685	OL334139
Lamiales	Clerodendrum inerme	Clerodendrum_inerme_HNHK_138	OK634734	OK637632	OK636686	OL334140
Lamiales	Clerodendrum inerme	Clerodendrum_inerme_HNHK_139	OK634735	OK637633	OK636687	NA
Lamiales	Clerodendrum inerme	Clerodendrum_inerme_HNSY_017	OK634736	NA	OK636688	NA
Lamiales	Clerodendrum inerme	Clerodendrum_inerme_HNSY_018	OK634737	NA	OK636689	NA
Lamiales	Clerodendrum inerme	Clerodendrum_inerme_HNSY_350	OK634738	OK637634	OK636690	NA
Lamiales	Clerodendrum inerme	Clerodendrum_inerme_HNSY_351	OK634739	NA	OK636691	NA
Lamiales	Clerodendrum inerme	Clerodendrum_inerme_HNSY_382	OK634740	OK637635	OK636692	NA
Lamiales	Clerodendrum inerme	Clerodendrum_inerme_HNSY_432	OK634741	OK637636	OK636693	OL334141
Lamiales	Clerodendrum inerme	Clerodendrum_inerme_HNSY_433	OK634742	OK637637	OK636694	OL334142

Lamiales	Clerodendrum inerme	Clerodendrum_inerme_HNSY_434	OK634743	OK637638	OK636695	OL334143
Lamiales	Clerodendrum inerme	Clerodendrum_inerme_HNWC_015	OK634744	NA	OK636696	NA
Lamiales	Clerodendrum inerme	Clerodendrum_inerme_HNWC_016	OK634745	NA	OK636697	NA
Lamiales	Clerodendrum inerme	Clerodendrum_inerme_GDSZ_987	NA	OK637606	NA	NA
Myrtales	Conocarpus erectus	Conocarpus_erectus_GDZH_672	OK634746	OK637639	OK636698	NA
Myrtales	Conocarpus erectus	Conocarpus_erectus_GDZH_673	OK634747	OK637640	OK636699	OL334144
Myrtales	Conocarpus erectus	Conocarpus_erectus_GDZH_674	OK634748	OK637641	OK636700	NA
Fabales	Crotalaria pallida	Crotalaria_pallida_HNSY_386	OK634749	OK637642	NA	OL334145
Poales	Cyperus malaccensis	Cyperus_malaccensis_GXLZ_903	OK634750	OK637643	NA	OL334146
Poales	Cyperus malaccensis	Cyperus_malaccensis_GXLZ_904	OK634751	OK637644	OK636701	NA
Poales	Cyperus malaccensis	Cyperus_malaccensis_GXLZ_905	OK634752	OK637645	OK636702	NA
Poales	Cyperus malaccensis	Cyperus_malaccensis_GXMW_791	OK634753	NA	OK636703	NA
Poales	Cyperus malaccensis	Cyperus_malaccensis_GXMW_793	OK634754	NA	OK636704	NA
Poales	Cyperus malaccensis	Cyperus_malaccensis_GXMW_794	OK634755	NA	OK636705	NA
Fabales	Dalbergia tonkinensis	Dalbergia_tonkinensis_HNWC_265	OK634756	OK637646	NA	OL334147
Fabales	Derris trifoliata	Derris_trifoliata_GDZH_641	OK634757	OK637647	OK636706	OL334148
Fabales	Derris trifoliata	Derris_trifoliata_GDZH_642	OK634758	OK637648	OK636707	OL334149
Fabales	Derris trifoliata	Derris_trifoliata_GDZH_643	OK634759	OK637649	OK636708	OL334150
Fabales	Derris trifoliata	Derris_trifoliata_GDZJ_466	OK634760	OK637650	OK636709	OL334151
Fabales	Derris trifoliata	Derris_trifoliata_GDZJ_467	OK634761	OK637651	OK636710	OL334152
Fabales	Derris trifoliata	Derris_trifoliata_GDZJ_468	OK634762	OK637652	OK636711	OL334153
Fabales	Derris trifoliata	Derris_trifoliata_GXLZ_911	OK634763	OK637653	OK636712	OL334154
Fabales	Derris trifoliata	Derris_trifoliata_GXLZ_912	OK634764	OK637654	OK636713	OL334155
Fabales	Derris trifoliata	Derris_trifoliata_GXLZ_913	OK634765	OK637655	OK636714	NA
Fabales	Derris trifoliata	Derris_trifoliata_HNHK_027	OK634766	OK637656	NA	OL334156
Fabales	Derris trifoliata	Derris_trifoliata_HNHK_125	OK634767	OK637657	NA	OL334157
Fabales	Derris trifoliata	Derris_trifoliata_HNQH_209	OK634768	OK637658	OK636715	OL334158
Fabales	Derris trifoliata	Derris_trifoliata_HNSY_377	OK634769	OK637659	OK636716	NA
Fabales	Derris trifoliata	Derris_trifoliata_HNSY_378	OK634770	OK637660	OK636717	OL334159
Fabales	Derris trifoliata	Derris_trifoliata_HNSY_428	OK634771	OK637661	OK636718	OL334160
Fabales	Derris trifoliata	Derris_trifoliata_HNSY_429	OK634772	OK637662	OK636719	OL334161
Fabales	Derris trifoliata	Derris_trifoliata_HNSY_430	OK634773	OK637663	OK636720	OL334162

Fabales	Derris trifoliata	Derris_trifoliata_HNWC_222	OK634774	OK637664	OK636721	OL334163
Fabales	Derris trifoliata	Derris_trifoliata_HNWC_223	OK634775	OK637665	OK636722	NA
Fabales	Derris trifoliata	Derris_trifoliata_HNWC_224	OK634776	OK637666	OK636723	OL334164
Lamiales	Dolichandrone spathacea	Dolichandrone_spathacea_GDZH_664	OK634777	NA	OK636724	OL334165
Lamiales	Dolichandrone spathacea	Dolichandrone_spathacea_GDZH_665	OK634778	NA	OK636725	OL334166
Lamiales	Dolichandrone spathacea	Dolichandrone_spathacea_GDZH_666	OK634779	NA	OK636726	OL334167
Lamiales	Dolichandrone spathacea	Dolichandrone_spathacea_HNHK_057	OK634780	NA	OK636727	OL334168
Malpighiales	Excoecaria agallocha	Excoecaria_agallocha_FJXM_311	OK634781	OK637667	NA	OL334169
Malpighiales	Excoecaria agallocha	Excoecaria_agallocha_GDST_553	OK634782	OK637668	NA	NA
Malpighiales	Excoecaria agallocha	Excoecaria_agallocha_GDST_554	OK634783	OK637669	NA	NA
Malpighiales	Excoecaria agallocha	Excoecaria_agallocha_GDST_555	OK634784	OK637670	NA	NA
Malpighiales	Excoecaria agallocha	Excoecaria_agallocha_GDSZ_947	OK634785	OK637671	NA	OL334170
Malpighiales	Excoecaria agallocha	Excoecaria_agallocha_GDSZ_948	OK634786	OK637672	NA	OL334171
Malpighiales	Excoecaria agallocha	Excoecaria_agallocha_GDSZ_949	OK634787	OK637673	NA	NA
Malpighiales	Excoecaria agallocha	Excoecaria_agallocha_GDZH_685	OK634788	OK637674	OK636728	OL334172
Malpighiales	Excoecaria agallocha	Excoecaria_agallocha_GDZH_686	OK634789	OK637675	OK636729	OL334173
Malpighiales	Excoecaria agallocha	Excoecaria_agallocha_GDZH_687	OK634790	OK637676	OK636730	OL334174
Malpighiales	Excoecaria agallocha	Excoecaria_agallocha_GDZJ_457	OK634791	OK637677	NA	OL334175
Malpighiales	Excoecaria agallocha	Excoecaria_agallocha_GDZJ_458	OK634792	OK637678	NA	OL334176
Malpighiales	Excoecaria agallocha	Excoecaria_agallocha_GDZJ_503	OK634793	OK637679	NA	OL334177
Malpighiales	Excoecaria agallocha	Excoecaria_agallocha_GDZJ_504	OK634794	OK637680	NA	OL334178
Malpighiales	Excoecaria agallocha	Excoecaria_agallocha_GDZJ_506	OK634795	OK637681	NA	OL334179
Malpighiales	Excoecaria agallocha	Excoecaria_agallocha_GXLZ_890	OK634796	OK637682	NA	OL334180
Malpighiales	Excoecaria agallocha	Excoecaria_agallocha_GXLZ_891	OK634797	OK637683	NA	OL334181
Malpighiales	Excoecaria agallocha	Excoecaria_agallocha_GXLZ_892	OK634798	OK637684	NA	OL334182
Malpighiales	Excoecaria agallocha	Excoecaria_agallocha_GXMW_785	OK634799	OK637685	OK636731	OL334183
Malpighiales	Excoecaria agallocha	Excoecaria_agallocha_GXMW_786	OK634800	OK637686	OK636732	OL334184
Malpighiales	Excoecaria agallocha	Excoecaria_agallocha_GXMW_787	OK634801	OK637687	OK636733	OL334185
Malpighiales	Excoecaria agallocha	Excoecaria_agallocha_GXTS_839	OK634802	OK637688	OK636738	OL334186
Malpighiales	Excoecaria agallocha	Excoecaria_agallocha_GXTS_840	OK634803	OK637689	OK636734	NA
Malpighiales	Excoecaria agallocha	Excoecaria_agallocha_GXTS_841	OK634804	OK637690	NA	OL334187
Malpighiales	Excoecaria agallocha	Excoecaria_agallocha_GXZZ_729	OK634805	OK637691	OK636735	OL334188

Malpighiales	Excoecaria agallocha	Excoecaria_agallocha_GXZZ_730	OK634806	OK637692	OK636736	OL334189
Malpighiales	Excoecaria agallocha	Excoecaria_agallocha_GXZZ_731	OK634807	OK637693	OK636737	OL334190
Malpighiales	Excoecaria agallocha	Excoecaria_agallocha_GXZZ_732	OK634808	OK637694	OK636739	OL334191
Malpighiales	Excoecaria agallocha	Excoecaria_agallocha_GXZZ_744	OK634809	OK637695	OK636742	OL334192
Malpighiales	Excoecaria agallocha	Excoecaria_agallocha_GXZZ_745	OK634810	OK637696	OK636743	OL334193
Malpighiales	Excoecaria agallocha	Excoecaria_agallocha_HNDZ_161	OK634811	OK637697	OK636744	OL334194
Malpighiales	Excoecaria agallocha	Excoecaria_agallocha_HNHK_073	OK634812	OK637698	OK636745	OL334195
Malpighiales	Excoecaria agallocha	Excoecaria_agallocha_HNHK_074	OK634813	OK637699	OK636746	OL334196
Malpighiales	Excoecaria agallocha	Excoecaria_agallocha_HNHK_075	OK634814	OK637700	OK636747	OL334197
Malpighiales	Excoecaria agallocha	Excoecaria_agallocha_HNQH_160	OK634815	OK637701	OK636748	OL334198
Malpighiales	Excoecaria agallocha	Excoecaria_agallocha_HNQH_162	OK634816	OK637702	OK636740	OL334199
Malpighiales	Excoecaria agallocha	Excoecaria_agallocha_HNQH_163	OK634817	NA	OK636749	OL334200
Malpighiales	Excoecaria agallocha	Excoecaria_agallocha_HNQH_164	OK634818	OK637703	OK636750	OL334201
Malpighiales	Excoecaria agallocha	Excoecaria_agallocha_HNSY_332	OK634819	OK637704	OK636741	OL334202
Malpighiales	Excoecaria agallocha	Excoecaria_agallocha_HNSY_333	OK634820	OK637705	OK636751	OL334203
Malpighiales	Excoecaria agallocha	Excoecaria_agallocha_HNSY_334	OK634821	OK637706	OK636752	OL334204
Malpighiales	Excoecaria agallocha	Excoecaria_agallocha_HNSY_362	OK634822	OK637707	OK636753	OL334205
Malpighiales	Excoecaria agallocha	Excoecaria_agallocha_HNSY_363	OK634823	OK637708	OK636754	OL334206
Malpighiales	Excoecaria agallocha	Excoecaria_agallocha_HNSY_395	OK634824	OK637709	NA	OL334207
Malpighiales	Excoecaria agallocha	Excoecaria_agallocha_HNSY_396	OK634825	OK637710	OK636755	OL334208
Malpighiales	Excoecaria agallocha	Excoecaria_agallocha_HNSY_397	OK634826	OK637711	OK636756	OL334209
Malpighiales	Excoecaria agallocha	Excoecaria_agallocha_HNWC_232	OK634827	OK637712	OK636757	OL334210
Malpighiales	Excoecaria agallocha	Excoecaria_agallocha_HNWC_233	OK634828	OK637713	OK636758	OL334211
Malpighiales	Excoecaria agallocha	Excoecaria_agallocha_HNWC_234	OK634829	OK637714	OK636759	OL334212
Malvales	Heritiera littoralis	Heritiera_littoralis_GDSZ_991	OK634830	OK637715	OK636760	OL334213
Malvales	Heritiera littoralis	Heritiera_littoralis_GDSZ_992	OK634831	OK637716	OK636761	OL334214
Malvales	Heritiera littoralis	Heritiera_littoralis_GDSZ_993	OK634832	OK637717	NA	NA
Malvales	Heritiera littoralis	Heritiera_littoralis_GDZH_628	OK634833	OK637718	OK636762	NA
Malvales	Heritiera littoralis	Heritiera_littoralis_GDZH_629	OK634834	OK637719	OK636763	OL334215
Malvales	Heritiera littoralis	Heritiera_littoralis_GDZH_630	OK634835	OK637720	OK636764	OL334216
Malvales	Heritiera littoralis	Heritiera_littoralis_GXZZ_723	OK634836	OK637721	OK636765	OL334217
Malvales	Heritiera littoralis	Heritiera_littoralis_GXZZ_724	OK634837	OK637722	OK636766	OL334218

Malvales	Heritiera littoralis	Heritiera_littoralis_GXZZ_725	OK634838	OK637723	OK636767	NA
Malvales	Heritiera littoralis	Heritiera_littoralis_GXZZ_757	OK634839	OK637724	OK636768	OL334219
Malvales	Heritiera littoralis	Heritiera_littoralis_HNHK_145	OK634840	NA	OK636769	OL334220
Malvales	Heritiera littoralis	Heritiera_littoralis_HNHK_146	OK634841	OK637725	OK636770	OL334221
Malvales	Heritiera littoralis	Heritiera_littoralis_HNWC_218	OK634842	OK637726	OK636771	NA
Malvales	Heritiera littoralis	Heritiera_littoralis_HNWC_219	OK634843	OK637727	OK636772	NA
Malvales	Heritiera littoralis	Heritiera_littoralis_HNWC_262	OK634844	OK637728	OK636773	OL334222
Laurales	Hernandia sonora	Hernandia_sonora_GDZH_669	OK634845	NA	OK636774	NA
Laurales	Hernandia sonora	Hernandia_sonora_HNHK_100	OK634846	NA	OK636775	OL334223
Laurales	Hernandia sonora	Hernandia_sonora_HNQH_193	OK634847	OK637729	OK636776	OL334224
Laurales	Hernandia sonora	Hernandia_sonora_HNQH_194	OK634848	OK637730	OK636777	OL334225
Malvales	Hibiscus hamabo	Hibiscus_hamabo_GDSZ_954	OK634849	OK637731	OK636778	NA
Malvales	Hibiscus hamabo	Hibiscus_hamabo_GDSZ_955	OK634850	OK637732	OK636780	OL334477
Malvales	Hibiscus hamabo	Hibiscus_hamabo_GDSZ_956	OK634851	OK637733	OK636781	OL334478
Malvales	Hibiscus tiliaceus	Hibiscus_tiliaceus_GXTS_817	OK634852	OK637749	NA	NA
Malvales	Hibiscus tiliaceus	Hibiscus_tiliaceus_HNWC_231	OK634853	OK637764	OK636802	NA
Malvales	Hibiscus tiliaceus	Hibiscus_tiliaceus_GXMW_778	OK634854	OK637747	NA	NA
Malvales	Hibiscus tiliaceus	Hibiscus_tiliaceus_GDSZ_953	OK634855	OK637734	OK636779	NA
Malvales	Hibiscus tiliaceus	Hibiscus_tiliaceus_GDZH_671	OK634856	OK637735	OK636782	OL334226
Malvales	Hibiscus tiliaceus	Hibiscus_tiliaceus_GDZJ_438	OK634857	OK637736	OK636783	OL334227
Malvales	Hibiscus tiliaceus	Hibiscus_tiliaceus_GDZJ_439	OK634858	OK637737	OK636784	OL334228
Malvales	Hibiscus tiliaceus	Hibiscus_tiliaceus_GDZJ_440	OK634859	OK637738	OK636785	OL334229
Malvales	Hibiscus tiliaceus	Hibiscus_tiliaceus_GDZJ_488	OK634860	OK637739	OK636786	NA
Malvales	Hibiscus tiliaceus	Hibiscus_tiliaceus_GDZJ_489	OK634861	OK637740	OK636787	NA
Malvales	Hibiscus tiliaceus	Hibiscus_tiliaceus_GDZJ_491	OK634862	OK637742	NA	NA
Malvales	Hibiscus tiliaceus	Hibiscus_tiliaceus_GXLZ_893	OK634863	OK637743	NA	NA
Malvales	Hibiscus tiliaceus	Hibiscus_tiliaceus_GXLZ_894	OK634864	OK637744	OK636789	NA
Malvales	Hibiscus tiliaceus	Hibiscus_tiliaceus_GXLZ_895	OK634865	OK637745	NA	NA
Malvales	Hibiscus tiliaceus	Hibiscus_tiliaceus_GXMW_777	OK634866	OK637746	OK636790	OL334230
Malvales	Hibiscus tiliaceus	Hibiscus_tiliaceus_GXMW_779	OK634867	OK637748	OK636791	NA
Malvales	Hibiscus tiliaceus	Hibiscus_tiliaceus_GXTS_819	OK634868	OK637751	NA	NA
Malvales	Hibiscus tiliaceus	Hibiscus_tiliaceus_GXZZ_720	OK634869	OK637752	NA	NA

Malvales	Hibiscus tiliaceus	Hibiscus_tiliaceus_GXZZ_721	OK634870	OK637753	OK636792	NA
Malvales	Hibiscus tiliaceus	Hibiscus_tiliaceus_GXZZ_722	OK634871	OK637754	OK636793	OL334231
Malvales	Hibiscus tiliaceus	Hibiscus_tiliaceus_HNDZ_171	OK634872	OK637755	OK636794	NA
Malvales	Hibiscus tiliaceus	Hibiscus_tiliaceus_HNDZ_172	OK634873	OK637756	OK636795	OL334232
Malvales	Hibiscus tiliaceus	Hibiscus_tiliaceus_HNDZ_173	OK634874	OK637757	NA	NA
Malvales	Hibiscus tiliaceus	Hibiscus_tiliaceus_HNSY_337	OK634875	OK637758	OK636796	OL334233
Malvales	Hibiscus tiliaceus	Hibiscus_tiliaceus_HNSY_338	OK634876	OK637759	OK636797	NA
Malvales	Hibiscus tiliaceus	Hibiscus_tiliaceus_HNSY_405	OK634877	OK637760	OK636798	NA
Malvales	Hibiscus tiliaceus	Hibiscus_tiliaceus_HNSY_406	OK634878	OK637761	OK636799	NA
Malvales	Hibiscus tiliaceus	Hibiscus_tiliaceus_HNWC_229	OK634879	OK637762	OK636800	NA
Malvales	Hibiscus tiliaceus	Hibiscus_tiliaceus_HNWC_230	OK634880	OK637763	OK636801	NA
Malvales	Hibiscus tiliaceus	Hibiscus_tiliaceus_GDZJ_490	NA	OK637741	OK636788	NA
Malvales	Hibiscus tiliaceus	Hibiscus_tiliaceus_GXTS_818	NA	OK637750	NA	NA
Gentianales	Hoya carnosa	Hoya_carnosa_HNSY_026	OK634881	OK637765	NA	OL334234
Gentianales	Hoya carnosa	Hoya_carnosa_HNSY_424	OK634882	OK637766	OK636803	OL334235
Gentianales	Hoya carnosa	Hoya_carnosa_HNSY_425	OK634883	NA	OK636804	OL334236
Gentianales	Hoya carnosa	Hoya_carnosa_HNSY_426	OK634884	OK637767	OK636805	OL334237
Gentianales	Hoya carnosa	Hoya_carnosa_HNSY_427	OK634885	NA	OK636806	OL334238
Gentianales	Hoya carnosa	Hoya_carnosa_HNWC_266	OK634886	OK637768	OK636807	OL334239
Solanales	Ipomoea pes-caprae	Ipomoea_pescaprae_GDST_526	OK634887	OK637779	OK636808	OL334240
Solanales	Ipomoea pes-caprae	Ipomoea_pescaprae_GDST_527	OK634888	OK637780	OK636809	OL334241
Solanales	Ipomoea pes-caprae	Ipomoea_pescaprae_GDST_528	OK634889	OK637781	OK636815	OL334242
Solanales	Ipomoea pes-caprae	Ipomoea_pescaprae_GDST_529	OK634890	OK637782	OK636810	OL334243
Solanales	Ipomoea pes-caprae	Ipomoea_pescaprae_GDST_552	OK634891	OK637783	OK636816	NA
Solanales	Ipomoea pes-caprae	Ipomoea_pescaprae_GDZJ_469	OK634892	OK637784	OK636811	OL334244
Solanales	Ipomoea pes-caprae	Ipomoea_pescaprae_GDZJ_470	OK634893	OK637785	OK636817	OL334245
Solanales	Ipomoea pes-caprae	Ipomoea_pescaprae_GDZJ_471	OK634894	OK637786	OK636818	OL334246
Solanales	Ipomoea pes-caprae	Ipomoea_pescaprae_GDZJ_507	OK634895	OK637787	NA	OL334247
Solanales	Ipomoea pes-caprae	Ipomoea_pescaprae_GDZJ_508	OK634896	OK637788	NA	OL334248
Solanales	Ipomoea pes-caprae	Ipomoea_pescaprae_GXLZ_909	OK634897	OK637769	OK636819	OL334249
Solanales	Ipomoea pes-caprae	Ipomoea_pescaprae_GXLZ_910	OK634898	OK637770	OK636812	OL334250
Solanales	Ipomoea pes-caprae	Ipomoea_pescaprae_GXMW_780	OK634899	OK637771	OK636820	OL334251

Solanales	Ipomoea pes-caprae	Ipomoea_pescaprae_GXMW_781	OK634900	OK637772	NA	OL334252
Solanales	Ipomoea pes-caprae	Ipomoea_pescaprae_GXTS_830	OK634901	OK637773	OK636821	OL334253
Solanales	Ipomoea pes-caprae	Ipomoea_pescaprae_GXTS_831	OK634902	OK637774	OK636822	OL334254
Solanales	Ipomoea pes-caprae	Ipomoea_pescaprae_GXTS_879	OK634903	OK637775	OK636813	OL334255
Solanales	Ipomoea pes-caprae	Ipomoea_pescaprae_HNDZ_200	OK634904	OK637776	OK636814	OL334256
Solanales	Ipomoea pes-caprae	Ipomoea_pescaprae_HNSY_369	OK634905	OK637777	NA	OL334257
Solanales	Ipomoea pes-caprae	Ipomoea_pescaprae_HNSY_370	OK634906	OK637778	NA	OL334258
Malpighiales	Kandelia obovata	Kandelia_obovata_FJLH_278	OK634907	OK637789	OK636823	OL334259
Malpighiales	Kandelia obovata	Kandelia_obovata_FJLH_279	OK634908	OK637790	OK636824	OL334260
Malpighiales	Kandelia obovata	Kandelia_obovata_FJLH_280	OK634909	OK637791	OK636825	OL334261
Malpighiales	Kandelia obovata	Kandelia_obovata_FJLH_281	OK634910	OK637792	OK636826	OL334262
Malpighiales	Kandelia obovata	Kandelia_obovata_FJQZ_292	OK634911	OK637793	OK636827	OL334263
Malpighiales	Kandelia obovata	Kandelia_obovata_FJQZ_293	OK634912	OK637794	OK636828	OL334264
Malpighiales	Kandelia obovata	Kandelia_obovata_FJQZ_294	OK634913	OK637795	NA	OL334265
Malpighiales	Kandelia obovata	Kandelia_obovata_FJXM_275	OK634914	OK637796	NA	OL334266
Malpighiales	Kandelia obovata	Kandelia_obovata_FJXM_276	OK634915	OK637797	OK636829	OL334267
Malpighiales	Kandelia obovata	Kandelia_obovata_FJXM_277	OK634916	OK637798	OK636830	OL334268
Malpighiales	Kandelia obovata	Kandelia_obovata_FJXM_305	OK634917	OK637799	OK636831	OL334269
Malpighiales	Kandelia obovata	Kandelia_obovata_FJXM_306	OK634918	OK637800	OK636832	OL334270
Malpighiales	Kandelia obovata	Kandelia_obovata_FJXM_307	OK634919	OK637801	OK636833	OL334271
Malpighiales	Kandelia obovata	Kandelia_obovata_FJXM_317	OK634920	OK637802	OK636834	OL334272
Malpighiales	Kandelia obovata	Kandelia_obovata_FJXM_318	OK634921	OK637803	OK636835	OL334273
Malpighiales	Kandelia obovata	Kandelia_obovata_FJXM_319	OK634922	OK637804	NA	OL334274
Malpighiales	Kandelia obovata	Kandelia_obovata_FJXM_320	OK634923	OK637805	NA	OL334275
Malpighiales	Kandelia obovata	Kandelia_obovata_GDSZ_978	OK634924	OK637806	NA	OL334276
Malpighiales	Kandelia obovata	Kandelia_obovata_GDSZ_979	OK634925	OK637807	NA	NA
Malpighiales	Kandelia obovata	Kandelia_obovata_GDSZ_980	OK634926	OK637808	NA	NA
Malpighiales	Kandelia obovata	Kandelia_obovata_GDZH_625	OK634927	OK637809	OK636836	OL334277
Malpighiales	Kandelia obovata	Kandelia_obovata_GDZH_626	OK634928	OK637810	OK636837	OL334278
Malpighiales	Kandelia obovata	Kandelia_obovata_GDZH_627	OK634929	OK637811	OK636838	OL334279
Malpighiales	Kandelia obovata	Kandelia_obovata_GDZJ_448	OK634930	OK637812	OK636839	OL334280
Malpighiales	Kandelia obovata	Kandelia_obovata_GDZJ_449	OK634931	OK637813	OK636840	OL334281

Malpighiales	Kandelia obovata	Kandelia_obovata_GDZJ_450	OK634932	OK637814	OK636842	NA
Malpighiales	Kandelia obovata	Kandelia_obovata_GDZJ_451	OK634933	OK637815	OK636841	OL334282
Malpighiales	Kandelia obovata	Kandelia_obovata_GDZJ_452	OK634934	OK637816	OK636843	OL334283
Malpighiales	Kandelia obovata	Kandelia_obovata_GDZJ_514	OK634935	OK637817	OK636844	OL334284
Malpighiales	Kandelia obovata	Kandelia_obovata_GDZJ_515	OK634936	OK637832	OK636845	OL334285
Malpighiales	Kandelia obovata	Kandelia_obovata_GDZJ_516	OK634937	OK637818	OK636846	NA
Malpighiales	Kandelia obovata	Kandelia_obovata_GXLZ_900	OK634938	OK637819	NA	OL334286
Malpighiales	Kandelia obovata	Kandelia_obovata_GXLZ_901	OK634939	OK637820	NA	OL334287
Malpighiales	Kandelia obovata	Kandelia_obovata_GXLZ_902	OK634940	NA	NA	NA
Malpighiales	Kandelia obovata	Kandelia_obovata_GXMW_795	OK634941	OK637821	NA	OL334288
Malpighiales	Kandelia obovata	Kandelia_obovata_GXMW_796	OK634942	OK637822	NA	OL334289
Malpighiales	Kandelia obovata	Kandelia_obovata_GXMW_797	OK634943	OK637823	NA	OL334290
Malpighiales	Kandelia obovata	Kandelia_obovata_GXTS_855	OK634944	NA	OK636847	NA
Malpighiales	Kandelia obovata	Kandelia_obovata_GXTS_856	OK634945	OK637824	NA	OL334291
Malpighiales	Kandelia obovata	Kandelia_obovata_GXTS_857	OK634946	OK637825	NA	OL334292
Malpighiales	Kandelia obovata	Kandelia_obovata_GXZZ_699	OK634947	OK637826	OK636848	OL334293
Malpighiales	Kandelia obovata	Kandelia_obovata_GXZZ_700	OK634948	OK637827	OK636849	OL334294
Malpighiales	Kandelia obovata	Kandelia_obovata_GXZZ_701	OK634949	OK637828	NA	OL334295
Malpighiales	Kandelia obovata	Kandelia_obovata_GXZZ_702	OK634950	OK637829	NA	OL334296
Malpighiales	Kandelia obovata	Kandelia_obovata_HNHK_123	OK634951	OK637830	NA	OL334297
Malpighiales	Kandelia obovata	Kandelia_obovata_HNHK_124	OK634952	NA	OK636850	OL334298
Malpighiales	Kandelia obovata	Kandelia_obovata_ZJWZ_600	OK634953	OK637831	OK636851	OL334299
Malpighiales	Kandelia obovata	Kandelia_obovata_ZJWZ_601	OK634954	OK637833	OK636852	OL334300
Malpighiales	Kandelia obovata	Kandelia_obovata_ZJWZ_602	OK634955	OK637834	OK636853	OL334301
Malpighiales	Kandelia obovata	Kandelia_obovata_ZJWZ_603	OK634956	OK637835	OK636854	OL334302
Malpighiales	Kandelia obovata	Kandelia_obovata_ZJWZ_604	OK634957	OK637836	OK636855	OL334303
Malpighiales	Kandelia obovata	Kandelia_obovata_ZJWZ_605	OK634958	OK637837	OK636856	OL334304
Malpighiales	Kandelia obovata	Kandelia_obovata_ZJWZ_606	OK634959	OK637838	OK636857	OL334305
Malpighiales	Kandelia obovata	Kandelia_obovata_ZJWZ_607	OK634960	OK637839	OK636858	OL334306
Myrtales	Laguncularia racemosa	Laguncularia_racemosa_FJXM_301	OK634961	OK637840	OK636859	OL334307
Myrtales	Laguncularia racemosa	Laguncularia_racemosa_FJXM_302	OK634962	OK637841	NA	OL334308
Myrtales	Laguncularia racemosa	Laguncularia_racemosa_FJXM_303	OK634963	OK637842	NA	OL334309

Myrtales	Laguncularia racemosa	Laguncularia_racemosa_FJXM_304	OK634964	OK637843	OK636860	OL334310
Myrtales	Laguncularia racemosa	Laguncularia_racemosa_GXTS_926	OK634965	OK637844	OK636861	OL334311
Myrtales	Laguncularia racemosa	Laguncularia_racemosa_GXTS_927	OK634966	OK637845	OK636862	OL334312
Myrtales	Laguncularia racemosa	Laguncularia_racemosa_GXTS_928	OK634967	OK637846	NA	OL334313
Myrtales	Laguncularia racemosa	Laguncularia_racemosa_HNHK_093	OK634968	OK637847	OK636863	OL334314
Myrtales	Laguncularia racemosa	Laguncularia_racemosa_HNHK_094	OK634969	OK637848	OK636864	OL334315
Myrtales	Laguncularia racemosa	Laguncularia_racemosa_HNHK_095	OK634970	OK637849	NA	NA
Myrtales	Laguncularia racemosa	Laguncularia_racemosa_HNSY_342	OK634971	OK637850	NA	NA
Myrtales	Laguncularia racemosa	Laguncularia_racemosa_HNSY_343	OK634972	OK637851	OK636865	NA
Myrtales	Laguncularia racemosa	Laguncularia_racemosa_HNSY_344	OK634973	OK637852	OK636866	NA
Myrtales	Laguncularia racemosa	Laguncularia_racemosa_HNSY_410	OK634974	OK637853	OK636867	OL334316
Fabales	Leucaena leucocephala	Leucaena_leucocephala_GDZH_684	OK634975	NA	OK636868	NA
Caryophyllales	Limonium sinense	Limonium_sinense_GXTS_835	OK634976	OK637854	OK636869	NA
Caryophyllales	Limonium sinense	Limonium_sinense_GXTS_836	OK634977	OK637855	OK636870	OL334317
Caryophyllales	Limonium sinense	Limonium_sinense_GXTS_837	OK634978	OK637856	OK636871	NA
Caryophyllales	Limonium sinense	Limonium_sinense_GXTS_838	OK634979	OK637857	OK636872	OL334318
Myrtales	Lumnitzera littorea	Lumnitzera_littorea_HNSY_400	OK634980	OK637858	NA	OL334319
Myrtales	Lumnitzera littorea	Lumnitzera_littorea_HNSY_401	OK634981	OK637859	OK636875	OL334320
Myrtales	Lumnitzera littorea	Lumnitzera_littorea_HNSY_402	OK634982	OK637860	OK636873	OL334321
Myrtales	Lumnitzera littorea	Lumnitzera_littorea_HNSY_403	OK634983	OK637861	OK636874	OL334322
Myrtales	Lumnitzera racemosa	Lumnitzera_racemosa_GDZH_658	OK634984	OK637862	OK636876	OL334323
Myrtales	Lumnitzera racemosa	Lumnitzera_racemosa_GDZH_659	OK634985	OK637863	OK636881	OL334324
Myrtales	Lumnitzera racemosa	Lumnitzera_racemosa_GDZH_660	OK634986	OK637864	OK636877	OL334325
Myrtales	Lumnitzera racemosa	Lumnitzera_racemosa_GXTS_811	OK634987	OK637865	OK636880	OL334326
Myrtales	Lumnitzera racemosa	Lumnitzera_racemosa_GXTS_812	OK634988	OK637866	NA	OL334327
Myrtales	Lumnitzera racemosa	Lumnitzera_racemosa_GXTS_813	OK634989	OK637867	NA	OL334328
Myrtales	Lumnitzera racemosa	Lumnitzera_racemosa_GXZZ_709	OK634990	OK637868	OK636878	OL334329
Myrtales	Lumnitzera racemosa	Lumnitzera_racemosa_GXZZ_710	OK634991	OK637869	OK636879	OL334330
Myrtales	Lumnitzera racemosa	Lumnitzera_racemosa_GXZZ_711	OK634992	OK637870	OK636882	OL334331
Myrtales	Lumnitzera racemosa	Lumnitzera_racemosa_GXZZ_712	OK634993	OK637871	OK636883	OL334332
Myrtales	Lumnitzera racemosa	Lumnitzera_racemosa_HNDZ_189	OK634994	OK637872	OK636888	OL334333
Myrtales	Lumnitzera racemosa	Lumnitzera_racemosa_HNDZ_190	OK634995	OK637873	OK636884	OL334334

Myrtales	Lumnitzera racemosa	Lumnitzera_racemosa_HNDZ_191	OK634996	OK637874	OK636889	OL334335
Myrtales	Lumnitzera racemosa	Lumnitzera_racemosa_HNDZ_192	OK634997	OK637875	OK636890	OL334336
Myrtales	Lumnitzera racemosa	Lumnitzera_racemosa_HNHK_096	OK634998	OK637876	OK636885	OL334337
Myrtales	Lumnitzera racemosa	Lumnitzera_racemosa_HNHK_097	OK634999	OK637877	OK636891	OL334338
Myrtales	Lumnitzera racemosa	Lumnitzera_racemosa_HNSY_345	OK635000	OK637878	OK636892	NA
Myrtales	Lumnitzera racemosa	Lumnitzera_racemosa_HNSY_346	OK635001	OK637879	OK636893	NA
Myrtales	Lumnitzera racemosa	Lumnitzera_racemosa_HNSY_347	OK635002	OK637880	OK636894	NA
Myrtales	Lumnitzera racemosa	Lumnitzera_racemosa_HNSY_411	OK635003	NA	NA	OL334339
Myrtales	Lumnitzera racemosa	Lumnitzera_racemosa_HNSY_412	OK635004	NA	NA	OL334340
Myrtales	Lumnitzera racemosa	Lumnitzera_racemosa_HNSY_413	OK635005	NA	NA	OL334341
Myrtales	Lumnitzera racemosa	Lumnitzera_racemosa_HNSY_414	OK635006	NA	NA	OL334342
Myrtales	Lumnitzera racemosa	Lumnitzera_racemosa_HNWC_225	OK635007	OK637881	OK636895	NA
Myrtales	Lumnitzera racemosa	Lumnitzera_racemosa_HNWC_226	OK635008	OK637882	OK636896	NA
Myrtales	Lumnitzera racemosa	Lumnitzera_racemosa_HNWC_227	OK635009	OK637883	OK636887	NA
Myrtales	Lumnitzera racemosa	Lumnitzera_racemosa_HNWC_228	OK635010	OK637884	OK636886	NA
Gentianales	Morinda citrifolia	Morinda_citrifolia_HNHK_064	OK635011	NA	OK636643	OL334343
Lamiales	Myoporum bontioides	Myoporum_bontioides_GDSZ_957	OK635012	OK637885	OK636897	OL334344
Lamiales	Myoporum bontioides	Myoporum_bontioides_GDZJ_509	OK635013	OK637886	NA	OL334345
Lamiales	Myoporum bontioides	Myoporum_bontioides_GXTS_851	OK635014	OK637887	OK636898	OL334346
Lamiales	Myoporum bontioides	Myoporum_bontioides_GXTS_852	OK635015	OK637888	OK636899	OL334347
Lamiales	Myoporum bontioides	Myoporum_bontioides_GXTS_853	OK635016	OK637889	NA	OL334348
Lamiales	Myoporum bontioides	Myoporum_bontioides_GXTS_854	OK635017	OK637890	NA	OL334349
Arecales	Nypa fruticans	Nypa_fruticans_HNHK_129	OK635018	NA	OK636900	OL334350
Arecales	Nypa fruticans	Nypa_fruticans_HNHK_130	OK635019	NA	OK636901	OL334351
Arecales	Nypa fruticans	Nypa_fruticans_HNHK_131	OK635020	NA	NA	OL334352
Pandanales	Pandanus tectorius	Pandanus_tectorius_GDSZ_969	OK635021	NA	NA	OL334353
Pandanales	Pandanus tectorius	Pandanus_tectorius_GDSZ_970	OK635022	NA	NA	OL334354
Pandanales	Pandanus tectorius	Pandanus_tectorius_GDSZ_971	OK635023	NA	NA	NA
Pandanales	Pandanus tectorius	Pandanus_tectorius_GXMW_792	OK635024	NA	NA	OL334355
Pandanales	Pandanus tectorius	Pandanus_tectorius_GXTS_814	OK635025	NA	NA	OL334356
Pandanales	Pandanus tectorius	Pandanus_tectorius_GXTS_815	OK635026	NA	NA	OL334357
Pandanales	Pandanus tectorius	Pandanus_tectorius_GXTS_816	OK635027	NA	NA	OL334358

Pandanales	Pandanus tectorius	Pandanus_tectorius_GXZZ_761	OK635028	NA	NA	OL334359
Pandanales	Pandanus tectorius	Pandanus_tectorius_GXZZ_762	OK635029	NA	NA	OL334360
Pandanales	Pandanus tectorius	Pandanus_tectorius_GXZZ_763	OK635030	NA	NA	OL334361
Pandanales	Pandanus tectorius	Pandanus_tectorius_HNSY_415	OK635031	NA	NA	OL334362
Myrtales	Pemphis acidula	Pemphis_acidula_HNQH_012	OK635032	NA	OK636902	NA
Myrtales	Pemphis acidula	Pemphis_acidula_HNQH_013	OK635033	NA	OK636903	NA
Myrtales	Pemphis acidula	Pemphis_acidula_HNQH_014	OK635034	NA	NA	NA
Myrtales	Pemphis acidula	Pemphis_acidula_HNQH_211	OK635035	OK637891	OK636904	NA
Myrtales	Pemphis acidula	Pemphis_acidula_HNWC_008	OK635036	OK637892	OK636907	OL334363
Myrtales	Pemphis acidula	Pemphis_acidula_HNWC_009	OK635037	NA	OK636905	NA
Myrtales	Pemphis acidula	Pemphis_acidula_HNWC_010	OK635038	OK637893	OK636906	NA
Myrtales	Pemphis acidula	Pemphis_acidula_HNWC_011	OK635039	NA	NA	NA
Lamiales	Phyla nodiflora	Phyla_nodiflora_GXLZ_914	OK635040	OK637894	OK636908	OL334364
Lamiales	Phyla nodiflora	Phyla_nodiflora_GXLZ_915	OK635041	OK637895	OK636909	OL334365
Lamiales	Phyla nodiflora	Phyla_nodiflora_GXLZ_916	OK635042	OK637896	OK636910	OL334366
Lamiales	Phyla nodiflora	Phyla_nodiflora_GXLZ_917	OK635043	OK637897	OK636911	OL334367
Asterales	Pluchea indica	Pluchea_indica_GDSZ_958	OK635044	OK637898	OK636912	NA
Asterales	Pluchea indica	Pluchea_indica_GDSZ_959	OK635045	OK637899	OK636913	OL334369
Asterales	Pluchea indica	Pluchea_indica_GDSZ_960	OK635046	OK637900	OK636914	NA
Asterales	Pluchea indica	Pluchea_indica_GDSZ_961	OK635047	OK637901	OK636915	NA
Asterales	Pluchea indica	Pluchea_indica_GDZH_618	OK635048	OK637902	OK636916	NA
Asterales	Pluchea indica	Pluchea_indica_GDZH_619	OK635049	OK637903	OK636917	NA
Asterales	Pluchea indica	Pluchea_indica_GDZH_620	OK635050	OK637904	NA	NA
Asterales	Pluchea indica	Pluchea_indica_GDZJ_476	OK635051	OK637905	OK636918	OL334370
Asterales	Pluchea indica	Pluchea_indica_GDZJ_510	OK635052	OK637906	OK636919	OL334371
Asterales	Pluchea indica	Pluchea_indica_GDZJ_511	OK635053	OK637907	OK636920	OL334372
Asterales	Pluchea indica	Pluchea_indica_GDZJ_512	OK635054	OK637908	OK636921	OL334373
Asterales	Pluchea indica	Pluchea_indica_GDZJ_513	OK635055	OK637909	OK636922	OL334368
Asterales	Pluchea indica	Pluchea_indica_GXLZ_882	OK635056	OK637910	OK636923	NA
Asterales	Pluchea indica	Pluchea_indica_GXLZ_883	OK635057	OK637911	OK636924	NA
Asterales	Pluchea indica	Pluchea_indica_GXLZ_884	OK635058	OK637912	OK636925	OL334374
Asterales	Pluchea indica	Pluchea_indica_GXMW_799	OK635059	OK637913	OK636926	NA

Asterales	Pluchea indica	Pluchea_indica_GXMW_800	OK635060	OK637914	OK636927	NA
Asterales	Pluchea indica	Pluchea_indica_GXMW_801	OK635061	OK637915	OK636928	NA
Asterales	Pluchea indica	Pluchea_indica_GXTS_805	OK635062	OK637916	OK636929	OL334375
Asterales	Pluchea indica	Pluchea_indica_GXTS_806	OK635063	OK637917	OK636930	NA
Asterales	Pluchea indica	Pluchea_indica_GXTS_807	OK635064	OK637918	OK636931	NA
Asterales	Pluchea indica	Pluchea_indica_GXZZ_749	OK635065	OK637919	NA	NA
Asterales	Pluchea indica	Pluchea_indica_GXZZ_750	OK635066	OK637920	OK636932	OL334376
Asterales	Pluchea indica	Pluchea_indica_GXZZ_751	OK635067	OK637921	OK636933	NA
Asterales	Pluchea indica	Pluchea_indica_HNDZ_184	OK635068	OK637922	OK636934	OL334377
Asterales	Pluchea indica	Pluchea_indica_HNDZ_185	OK635069	OK637923	OK636935	OL334378
Asterales	Pluchea indica	Pluchea_indica_HNDZ_186	OK635070	OK637924	OK636936	OL334379
Asterales	Pluchea indica	Pluchea_indica_HNHK_092	OK635071	OK637925	OK636937	OL334380
Fabales	Pongamia pinnata	Pongamia_pinnata_GDZJ_453	OK635072	OK637926	OK636941	NA
Fabales	Pongamia pinnata	Pongamia_pinnata_GDZJ_454	OK635073	OK637927	OK636938	NA
Fabales	Pongamia pinnata	Pongamia_pinnata_GDZJ_455	OK635074	OK637928	OK636939	NA
Fabales	Pongamia pinnata	Pongamia_pinnata_GDZJ_456	OK635075	OK637929	OK636940	NA
Fabales	Pongamia pinnata	Pongamia_pinnata_GXTS_824	OK635076	OK637930	OK636942	NA
Fabales	Pongamia pinnata	Pongamia_pinnata_GXTS_825	OK635077	OK637931	OK636943	OL334381
Fabales	Pongamia pinnata	Pongamia_pinnata_GXTS_826	OK635078	OK637932	OK636944	OL334382
Fabales	Pongamia pinnata	Pongamia_pinnata_GXZZ_689	OK635079	OK637933	OK636945	OL334383
Fabales	Pongamia pinnata	Pongamia_pinnata_GXZZ_690	OK635080	OK637934	OK636946	OL334384
Fabales	Pongamia pinnata	Pongamia_pinnata_GXZZ_691	OK635081	OK637935	OK636947	OL334385
Fabales	Pongamia pinnata	Pongamia_pinnata_HNHK_058	OK635082	OK637936	NA	NA
Fabales	Pongamia pinnata	Pongamia_pinnata_HNHK_127	OK635083	OK637938	OK636948	NA
Fabales	Pongamia pinnata	Pongamia_pinnata_HNHK_128	OK635084	OK637939	OK636949	NA
Fabales	Pongamia pinnata	Pongamia_pinnata_HNHK_126	NA	OK637937	NA	NA
Caryophyllales	Portulaca pilosa	Portulaca_pilosa_GXLZ_918	OK635085	OK637940	OK636950	OL334386
Lamiales	Premna obtusifolia	Premna_obtusifolia_GXTS_876	OK635086	OK637941	OK636956	OL334387
Lamiales	Premna obtusifolia	Premna_obtusifolia_GXTS_877	OK635087	OK637942	OK636951	OL334388
Lamiales	Premna obtusifolia	Premna_obtusifolia_GXTS_878	OK635088	OK637943	OK636952	OL334389
Lamiales	Premna obtusifolia	Premna_obtusifolia_GXZZ_733	OK635089	OK637944	OK636955	OL334390
Lamiales	Premna obtusifolia	Premna_obtusifolia_GXZZ_767	OK635090	OK637945	OK636954	OL334391

Lamiales	Premna obtusifolia	Premna_obtusifolia_HNHK_062	OK635091	OK637946	OK636953	OL334392
Lamiales	Premna obtusifolia	Premna_obtusifolia_HNHK_063	OK635092	OK637947	OK636957	OL334393
Lamiales	Premna obtusifolia	Premna_obtusifolia_HNWC_019	OK635093	OK637948	OK636958	OL334394
Lamiales	Premna obtusifolia	Premna_obtusifolia_HNWC_020	OK635094	OK637949	OK636959	OL334395
Malpighiales	Rhizophora apiculata	Rhizophora_apiculata_HNWC_257	OK634626	OK637970	NA	NA
Malpighiales	Rhizophora apiculata	Rhizophora_apiculata_HNDZ_216	OK635095	OK637950	NA	NA
Malpighiales	Rhizophora apiculata	Rhizophora_apiculata_HNDZ_217	OK635096	OK637951	NA	NA
Malpighiales	Rhizophora apiculata	Rhizophora_apiculata_HNHK_149	OK635097	OK637952	NA	NA
Malpighiales	Rhizophora apiculata	Rhizophora_apiculata_HNHK_150	OK635098	OK637953	NA	NA
Malpighiales	Rhizophora apiculata	Rhizophora_apiculata_HNSY_352	OK635099	OK637954	NA	NA
Malpighiales	Rhizophora apiculata	Rhizophora_apiculata_HNSY_353	OK635100	OK637955	NA	NA
Malpighiales	Rhizophora apiculata	Rhizophora_apiculata_HNSY_354	OK635101	OK637956	NA	NA
Malpighiales	Rhizophora apiculata	Rhizophora_apiculata_HNSY_355	OK635102	OK637957	NA	NA
Malpighiales	Rhizophora apiculata	Rhizophora_apiculata_HNSY_356	OK635103	OK637958	NA	NA
Malpighiales	Rhizophora apiculata	Rhizophora_apiculata_HNSY_357	OK635104	OK637959	NA	NA
Malpighiales	Rhizophora apiculata	Rhizophora_apiculata_HNSY_358	OK635105	OK637960	NA	NA
Malpighiales	Rhizophora apiculata	Rhizophora_apiculata_HNSY_383	OK635106	OK637961	OK636961	NA
Malpighiales	Rhizophora apiculata	Rhizophora_apiculata_HNSY_384	OK635107	OK637962	OK636962	NA
Malpighiales	Rhizophora apiculata	Rhizophora_apiculata_HNSY_435	OK635108	OK637963	NA	OL334398
Malpighiales	Rhizophora apiculata	Rhizophora_apiculata_HNSY_436	OK635109	OK637964	NA	NA
Malpighiales	Rhizophora apiculata	Rhizophora_apiculata_HNSY_437	OK635110	OK637965	NA	NA
Malpighiales	Rhizophora apiculata	Rhizophora_apiculata_HNWC_253	OK635111	OK637966	NA	OL334399
Malpighiales	Rhizophora apiculata	Rhizophora_apiculata_HNWC_254	OK635112	OK637967	NA	NA
Malpighiales	Rhizophora apiculata	Rhizophora_apiculata_HNWC_255	OK635113	OK637968	NA	NA
Malpighiales	Rhizophora apiculata	Rhizophora_apiculata_HNWC_256	OK635114	OK637969	NA	NA
Malpighiales	Rhizophora apiculata	Rhizophora_apiculata_HNWC_258	OK635115	OK637971	NA	NA
Malpighiales	Rhizophora mangle	Rhizophora_mangle_HNHK_108	OK635116	OK637972	OK636960	OL334400
Malpighiales	Rhizophora mucronata	Rhizophora_mucronata_HNHK_082	OK635117	OK637973	NA	OL334401
Malpighiales	Rhizophora mucronata	Rhizophora_mucronata_HNHK_083	OK635118	OK637974	NA	OL334402
Malpighiales	Rhizophora mucronata	Rhizophora_mucronata_HNHK_084	OK635119	OK637975	NA	OL334403
Malpighiales	Rhizophora stylosa	Rhizophora_stylosa_GDZJ_519	OK635120	NA	NA	NA
Malpighiales	Rhizophora stylosa	Rhizophora_stylosa_GDZJ_520	OK635121	OK637976	NA	NA

Malpighiales	Rhizophora stylosa	Rhizophora_stylosa_GDZJ_521	OK635122		NA	NA
Malpighiales	Rhizophora stylosa	Rhizophora_stylosa_GDZJ_522	OK635123	NA	NA	NA
Malpighiales	Rhizophora stylosa	Rhizophora_stylosa_GXTS_844	OK635124	OK637977	NA	NA
Malpighiales	Rhizophora stylosa	Rhizophora_stylosa_GXTS_845	OK635125	OK637978	NA	NA
Malpighiales	Rhizophora stylosa	Rhizophora_stylosa_GXTS_846	OK635126	OK637979	NA	NA
Malpighiales	Rhizophora stylosa	Rhizophora_stylosa_GXTS_847	OK635127	OK637980	NA	NA
Malpighiales	Rhizophora stylosa	Rhizophora_stylosa_GXZZ_706	OK635128	OK637981	NA	NA
Malpighiales	Rhizophora stylosa	Rhizophora_stylosa_GXZZ_707	OK635129	OK637982	NA	NA
Malpighiales	Rhizophora stylosa	Rhizophora_stylosa_GXZZ_708	OK635130	OK637983	NA	NA
Malpighiales	Rhizophora stylosa	Rhizophora_stylosa_HNDZ_166	OK635131	OK637984	OK636966	NA
Malpighiales	Rhizophora stylosa	Rhizophora_stylosa_HNDZ_167	OK635132	OK637985	OK636967	NA
Malpighiales	Rhizophora stylosa	Rhizophora_stylosa_HNDZ_168	OK635133	OK637986	OK636963	OL334404
Malpighiales	Rhizophora stylosa	Rhizophora_stylosa_HNDZ_169	OK635134	OK637987	OK636964	OL334405
Malpighiales	Rhizophora stylosa	Rhizophora_stylosa_HNDZ_170	OK635135	OK637988	OK636965	NA
Malpighiales	Rhizophora stylosa	Rhizophora_stylosa_HNHK_079	OK635136	OK637989	NA	OL334406
Malpighiales	Rhizophora stylosa	Rhizophora_stylosa_HNHK_080	OK635137	OK637990	NA	OL334407
Malpighiales	Rhizophora stylosa	Rhizophora_stylosa_HNHK_081	OK635138	OK637991	NA	OL334408
Malpighiales	Rhizophora stylosa	Rhizophora_stylosa_HNSY_335	OK635139	OK637992	NA	OL334409
Malpighiales	Rhizophora stylosa	Rhizophora_stylosa_HNSY_336	OK635140	OK637993	NA	OL334410
Malpighiales	Rhizophora x lamarckii	Rhizophora_lamarckii_HNDZ_187	OK635281	OK638121	NA	NA
Malpighiales	Rhizophora x lamarckii	Rhizophora_lamarckii_HNDZ_188	OK635282	OK638122	NA	OL334511
Asterales	Scaevola hainanensis	Scaevola_hainanensis_GXZZ_738	OK635141	OK637995	OK636969	OL334411
Asterales	Scaevola hainanensis	Scaevola_hainanensis_GXZZ_739	OK635142	OK637996	OK636970	OL334412
Asterales	Scaevola hainanensis	Scaevola_hainanensis_GXZZ_740	OK635143	OK637997	OK636971	OL334414
Asterales	Scaevola hainanensis	Scaevola_hainanensis_HNHK_071	OK635144	OK637998	OK636972	OL334413
Asterales	Scaevola hainanensis	Scaevola_hainanensis_GXZZ_734	OK635145	OK637994	OK636968	OL334415
Asterales	Scaevola sericea	Scaevola_sericea_GXZZ_735	OK635146	OK637999	OK636973	OL334416
Asterales	Scaevola sericea	Scaevola_sericea_GXZZ_736	OK635147	OK638000	OK636974	OL334417
Asterales	Scaevola sericea	Scaevola_sericea_GXZZ_737	OK635148	OK638001	OK636975	OL334418
Asterales	Scaevola sericea	Scaevola_sericea_HNHK_059	OK635149	OK638002	OK636976	NA
Asterales	Scaevola sericea	Scaevola_sericea_HNHK_060	OK635150	OK638003	OK636977	OL334419
Asterales	Scaevola sericea	Scaevola_sericea_HNHK_061	OK635151	OK638004	OK636978	OL334420

Asterales	Scaevola sericea	Scaevola_sericea_HNSY_023	OK635152	OK638005	OK636979	NA
Asterales	Scaevola sericea	Scaevola_sericea_HNSY_024	OK635153	OK638006	OK636980	OL334421
Asterales	Scaevola sericea	Scaevola_sericea_HNSY_025	OK635154	OK638007	OK636981	OL334422
Asterales	Scaevola sericea	Scaevola_sericea_HNSY_331	OK635155	OK638008	OK636982	OL334423
Asterales	Scaevola sericea	Scaevola_sericea_HNWC_021	OK635156	OK638009	OK636983	OL334424
Asterales	Scaevola sericea	Scaevola_sericea_HNWC_022	OK635157	OK638010	OK636984	OL334425
Poales	Scirpus mariqueter	Scirpus_mariqueter_GXLZ_923	OK635158	OK638011	OK636985	OL334426
Poales	Scirpus mariqueter	Scirpus_mariqueter_GXLZ_924	OK635159	OK638012	OK636986	NA
Poales	Scirpus mariqueter	Scirpus_mariqueter_GXLZ_925	OK635160	OK638013	OK636987	NA
Gentianales	Scyphiphora hydrophyllacea	Scyphiphora_hydrophyllacea_HNWC_006	OK635161	OK638014	OK636990	OL334427
Saxifragales	Sedum lineare	Sedum_lineare_GDZH_681	OK635162	OK638015	OK636991	NA
Saxifragales	Sedum lineare	Sedum_lineare_GDZH_682	OK635163	OK638016	OK636992	NA
Saxifragales	Sedum lineare	Sedum_lineare_GDZH_683	OK635164	OK638017	OK636993	NA
Fabales	Sesbania cannabina	Sesbania_cannabina_HNDZ_158	OK635165	OK638018	OK636989	NA
Fabales	Sesbania cannabina	Sesbania_cannabina_HNDZ_159	OK635166	OK638019	OK636988	NA
Caryophyllales	Sesuvium portulacastrum	Sesuvium_portulacastrum_FJQZ_286	OK635167	OK638020	NA	OL334428
Caryophyllales	Sesuvium portulacastrum	Sesuvium_portulacastrum_FJQZ_287	OK635168	OK638021	NA	OL334429
Caryophyllales	Sesuvium portulacastrum	Sesuvium_portulacastrum_FJQZ_288	OK635169	OK638022	OK636994	OL334430
Caryophyllales	Sesuvium portulacastrum	Sesuvium_portulacastrum_GXTS_843	OK635170	OK638023	OK636995	OL334431
Malvales	Sida acuta	Sida_acuta_HNSY_367	OK635171	OK638024	NA	NA
Malvales	Sida acuta	Sida_acuta_HNSY_404	OK635172	OK638025	OK636996	NA
Myrtales	Sonneratia alba	Sonneratia_alba_HNHK_144	OK635173	OK638026	NA	OL334432
Myrtales	Sonneratia alba	Sonneratia_alba_HNQH_154	OK635174	OK638027	NA	NA
Myrtales	Sonneratia alba	Sonneratia_alba_HNQH_155	OK635175	OK638028	NA	OL334433
Myrtales	Sonneratia alba	Sonneratia_alba_HNSY_389	OK635176	OK638031	OK637000	OL334437
Myrtales	Sonneratia alba	Sonneratia_alba_HNWC_001	OK635177	NA	NA	NA
Myrtales	Sonneratia alba	Sonneratia_alba_HNHK_056	NA	OK638127	NA	NA
Myrtales	Sonneratia alba	Sonneratia_alba_HNSY_364	NA	OK638029	OK636997	OL334434
Myrtales	Sonneratia alba	Sonneratia_alba_HNSY_365	NA	OK638030	OK636998	OL334435
Myrtales	Sonneratia alba	Sonneratia_alba_HNSY_366	NA	NA	OK636999	OL334436
Myrtales	Sonneratia apetala	Sonneratia_apetala_FJXM_312	OK635178	OK638032	OK637001	OL334438
Myrtales	Sonneratia apetala	Sonneratia_apetala_FJXM_313	OK635179	OK638033	OK637002	OL334439

Myrtales	Sonneratia apetala	Sonneratia_apetala_FJXM_314	OK635180	OK638034	OK637003	OL334440
Myrtales	Sonneratia apetala	Sonneratia_apetala_FJXM_315	OK635181	OK638035	OK637004	OL334441
Myrtales	Sonneratia apetala	Sonneratia_apetala_FJXM_316	OK635182	OK638036	OK637005	OL334442
Myrtales	Sonneratia apetala	Sonneratia_apetala_GDST_532	OK635183	OK638037	OK637006	OL334443
Myrtales	Sonneratia apetala	Sonneratia_apetala_GDST_533	OK635184	OK638038	OK637007	OL334444
Myrtales	Sonneratia apetala	Sonneratia_apetala_GDST_535	OK635185	OK638039	OK637008	OL334445
Myrtales	Sonneratia apetala	Sonneratia_apetala_GDST_544	OK635186	OK638040	OK637009	OL334446
Myrtales	Sonneratia apetala	Sonneratia_apetala_GDST_545	OK635187	OK638041	OK637010	OL334447
Myrtales	Sonneratia apetala	Sonneratia_apetala_GDST_546	OK635188	OK638042	OK637011	OL334448
Myrtales	Sonneratia apetala	Sonneratia_apetala_GDST_547	OK635189	OK638043	OK637012	OL334449
Myrtales	Sonneratia apetala	Sonneratia_apetala_GDSZ_984	OK635190	OK638044	NA	OL334450
Myrtales	Sonneratia apetala	Sonneratia_apetala_GDZH_621	OK635191	OK638045	OK637013	OL334451
Myrtales	Sonneratia apetala	Sonneratia_apetala_GDZH_622	OK635192	OK638046	OK637014	OL334452
Myrtales	Sonneratia apetala	Sonneratia_apetala_GDZH_623	OK635193	OK638047	OK637015	OL334453
Myrtales	Sonneratia apetala	Sonneratia_apetala_GDZH_624	OK635194	OK638048	OK637016	OL334454
Myrtales	Sonneratia apetala	Sonneratia_apetala_GDZJ_475	OK635195	OK638049	OK637017	OL334455
Myrtales	Sonneratia apetala	Sonneratia_apetala_GDZJ_496	OK635196	OK638050	OK637018	OL334456
Myrtales	Sonneratia apetala	Sonneratia_apetala_GDZJ_497	OK635197	OK638051	OK637019	OL334457
Myrtales	Sonneratia apetala	Sonneratia_apetala_GDZJ_502	OK635198	OK638052	NA	NA
Myrtales	Sonneratia apetala	Sonneratia_apetala_GXLZ_920	OK635199	OK638053	OK637020	OL334458
Myrtales	Sonneratia apetala	Sonneratia_apetala_GXLZ_921	OK635200	OK638054	OK637021	OL334459
Myrtales	Sonneratia apetala	Sonneratia_apetala_GXLZ_922	OK635201	OK638055	OK637022	OL334460
Myrtales	Sonneratia apetala	Sonneratia_apetala_GXMW_788	OK635202	OK638056	OK637023	NA
Myrtales	Sonneratia apetala	Sonneratia_apetala_GXMW_789	OK635203	OK638057	OK637024	NA
Myrtales	Sonneratia apetala	Sonneratia_apetala_GXMW_790	OK635204	OK638058	OK637025	NA
Myrtales	Sonneratia apetala	Sonneratia_apetala_GXTS_842	OK635205	OK638059	OK637026	NA
Myrtales	Sonneratia apetala	Sonneratia_apetala_GXTS_933	OK635206	OK638060	OK637027	OL334461
Myrtales	Sonneratia apetala	Sonneratia_apetala_GXTS_934	OK635207	OK638061	OK637028	OL334462
Myrtales	Sonneratia apetala	Sonneratia_apetala_HNHK_044	OK635208	OK638062	OK637029	OL334463
Myrtales	Sonneratia apetala	Sonneratia_apetala_HNHK_135	OK635209	OK638063	OK637030	OL334464
Myrtales	Sonneratia apetala	Sonneratia_apetala_HNSY_005	OK635210	NA	OK637031	NA
Myrtales	Sonneratia apetala	Sonneratia_apetala_HNSY_030	OK635211	NA	OK637032	OL334465

Myrtales	Sonneratia caseolaris	Sonneratia_caseolaris_GDSZ_950	OK635212	OK638064	OK637033	OL334466
Myrtales	Sonneratia caseolaris	Sonneratia_caseolaris_GDSZ_951	OK635213	OK638065	OK637034	OL334467
Myrtales	Sonneratia caseolaris	Sonneratia_caseolaris_GDSZ_952	OK635214	OK638066	OK637035	OL334468
Myrtales	Sonneratia caseolaris	Sonneratia_caseolaris_HNHK_077	OK635215	OK638068	OK637037	OL334470
Myrtales	Sonneratia caseolaris	Sonneratia_caseolaris_HNHK_078	OK635216	OK638069	OK637038	OL334471
Myrtales	Sonneratia caseolaris	Sonneratia_caseolaris_HNQH_165	OK635217	OK638070	OK637039	OL334472
Myrtales	Sonneratia caseolaris	Sonneratia_caseolaris_HNSY_003	OK635218	NA	OK637040	NA
Myrtales	Sonneratia caseolaris	Sonneratia_caseolaris_HNWC_268	OK635219	OK638071	OK637041	OL334473
Myrtales	Sonneratia caseolaris	Sonneratia_caseolaris_HNHK_076	NA	OK638067	OK637036	OL334469
Myrtales	Sonneratia ovata	Sonneratia_ovata_HNHK_104	OK635220	OK638072	OK637042	OL334474
Myrtales	Sonneratia ovata	Sonneratia_ovata_HNHK_105	OK635221	OK638073	OK637043	OL334475
Myrtales	Sonneratia ovata	Sonneratia_ovata_HNHK_106	OK635222	OK638074	OK637044	NA
Myrtales	Sonneratia ovata	Sonneratia_ovata_HNHK_107	OK635223	OK638075	OK637045	OL334476
Myrtales	Sonneratia x gulngai	Sonneratia_gulngai_HNSY_029	OK635286	NA	NA	NA
Myrtales	Sonneratia x gulngai	Sonneratia_gulngai_HNSY_031	OK635287	NA	NA	NA
Myrtales	Sonneratia x gulngai	Sonneratia_gulngai_HNSY_032	OK635288	NA	NA	NA
Myrtales	Sonneratia x gulngai	Sonneratia_gulngai_HNSY_034	OK635289	NA	NA	NA
Myrtales	Sonneratia x gulngai	Sonneratia_gulngai_HNHK_122	OK635285	OK638123	NA	OL334507
Myrtales	Sonneratia x hainanensis	Sonneratia_hainanensis_HNWC_028	OK635284	OK638124	NA	OL334508
Myrtales	Sonneratia x hainanensis	Sonneratia_hainanensis_HNHK_072	OK635290	OK638125	NA	OL334509
Myrtales	Sonneratia x hainanensis	Sonneratia_hainanensis_HNWC_004	OK635283	OK638126	NA	NA
Myrtales	Sonneratia x zhongcairongii	Sonneratia_zhongcairongii_HNHK_055	OK635291	OK638120	NA	OL334506
Lamiales	Stachytarpheta jamaicensis	Stachytarpheta_jamaicensis_HNSY_407	OK635224	NA	OK637046	OL334479
Lamiales	Stachytarpheta jamaicensis	Stachytarpheta_jamaicensis_HNSY_408	OK635225	NA	OK637047	OL334480
Caryophyllales	Suaeda australis	Suaeda_australis_GXTS_832	OK635226	OK638076	OK637049	OL334481
Caryophyllales	Suaeda australis	Suaeda_australis_GXTS_833	OK635227	OK638077	OK637050	OL334482
Caryophyllales	Suaeda australis	Suaeda_australis_GXTS_834	OK635228	OK638078	OK637051	OL334483
Caryophyllales	Suaeda australis	Suaeda_australis_GXZZ_741	OK635229	OK638079	OK637052	OL334484
Caryophyllales	Suaeda australis	Suaeda_australis_GXZZ_742	OK635230	OK638080	OK637053	OL334485
Caryophyllales	Suaeda australis	Suaeda_australis_GXZZ_743	OK635231	OK638081	OK637054	OL334486
Caryophyllales	Suaeda glauca	Suaeda_glauca_ZJWZ_608	OK635232	NA	OK637055	OL334487
Caryophyllales	Suaeda glauca	Suaeda_glauca_ZJWZ_609	OK635233	OK638082	OK637056	OL334488

Caryophyllales	Suaeda glauca	Suaeda_glauca_ZJWZ_610	OK635234	OK638083	OK637057	OL334489
Caryophyllales	Suaeda glauca	Suaeda_glauca_ZJWZ_611	OK635235	OK638084	OK637058	OL334490
Fabales	Tephrosia purpurea	Tephrosia_purpurea_HNDZ_174	OK635236	NA	OK637059	NA
Fabales	Tephrosia purpurea	Tephrosia_purpurea_HNDZ_175	OK635237	NA	OK637060	NA
Fabales	Tephrosia purpurea	Tephrosia_purpurea_HNDZ_176	OK635238	NA	OK637061	NA
Malvales	Thespesia populnea	Thespesia_populnea_GDSZ_988	OK635239	OK638085	OK637062	NA
Malvales	Thespesia populnea	Thespesia_populnea_GDSZ_989	OK635240	OK638086	NA	NA
Malvales	Thespesia populnea	Thespesia_populnea_GDSZ_990	OK635241	OK638087	NA	NA
Malvales	Thespesia populnea	Thespesia_populnea_GDZH_632	OK635242	OK638088	OK637063	NA
Malvales	Thespesia populnea	Thespesia_populnea_GDZH_633	OK635243	OK638089	OK637064	NA
Malvales	Thespesia populnea	Thespesia_populnea_GDZH_634	OK635244	OK638090	OK637065	NA
Malvales	Thespesia populnea	Thespesia_populnea_GXTS_870	OK635245	OK638091	OK637066	NA
Malvales	Thespesia populnea	Thespesia_populnea_GXTS_871	OK635246	OK638092	OK637067	NA
Malvales	Thespesia populnea	Thespesia_populnea_GXTS_872	OK635247	OK638093	OK637068	NA
Malvales	Thespesia populnea	Thespesia_populnea_GXZZ_754	OK635248	OK638094	OK637069	NA
Malvales	Thespesia populnea	Thespesia_populnea_GXZZ_756	OK635249	OK638095	OK637070	NA
Malvales	Thespesia populnea	Thespesia_populnea_HNHK_140	OK635250	OK638096	NA	OL334491
Malvales	Thespesia populnea	Thespesia_populnea_HNHK_142	OK635251	OK638097	NA	OL334492
Malvales	Thespesia populnea	Thespesia_populnea_HNHK_143	OK635252	OK638098	NA	NA
Malvales	Thespesia populnea	Thespesia_populnea_HNWC_220	OK635253	OK638099	OK637071	NA
Rosales	Trema tomentosa	Trema_tomentosa_HNDZ_210	OK635254	OK638100	OK637048	NA
Malvales	Waltheria indica	Waltheria_indica_HNDZ_182	OK635255	OK638101	OK637072	OL334493
Malvales	Waltheria indica	Waltheria_indica_HNDZ_183	OK635256	OK638102	OK637073	OL334494
Sapindales	Xylocarpus granatum	Xylocarpus_granatum_HNHK_114	OK635257	OK638103	NA	OL334495
Sapindales	Xylocarpus granatum	Xylocarpus_granatum_HNHK_115	OK635258	OK638104	NA	OL334496
Sapindales	Xylocarpus granatum	Xylocarpus_granatum_HNHK_116	OK635259	OK638105	NA	OL334497
Sapindales	Xylocarpus granatum	Xylocarpus_granatum_HNHK_117	OK635260	OK638106	NA	OL334498
Sapindales	Xylocarpus granatum	Xylocarpus_granatum_HNSY_371	OK635261	OK638107	NA	NA
Sapindales	Xylocarpus granatum	Xylocarpus_granatum_HNSY_372	OK635262	OK638108	NA	NA
Sapindales	Xylocarpus granatum	Xylocarpus_granatum_HNSY_373	OK635263	OK638109	NA	NA
Sapindales	Xylocarpus granatum	Xylocarpus_granatum_HNSY_416	OK635264	OK638110	NA	NA
Sapindales	Xylocarpus granatum	Xylocarpus_granatum_HNSY_417	OK635265	OK638111	NA	OL334499

Sapindales	Xylocarpus granatum	Xylocarpus_granatum_HNSY_418	OK635266	OK638112	NA	OL334500
Sapindales	Xylocarpus granatum	Xylocarpus_granatum_HNWC_235	OK635267	OK638113	NA	OL334501
Sapindales	Xylocarpus granatum	Xylocarpus_granatum_HNWC_238	OK635268	OK638114	NA	NA
Sapindales	Xylocarpus granatum	Xylocarpus_granatum_HNWC_239	OK635269	OK638115	NA	OL334502
Sapindales	Xylocarpus granatum	Xylocarpus_granatum_HNWC_264	OK635270	OK638116	NA	OL334503

NA: not applicable

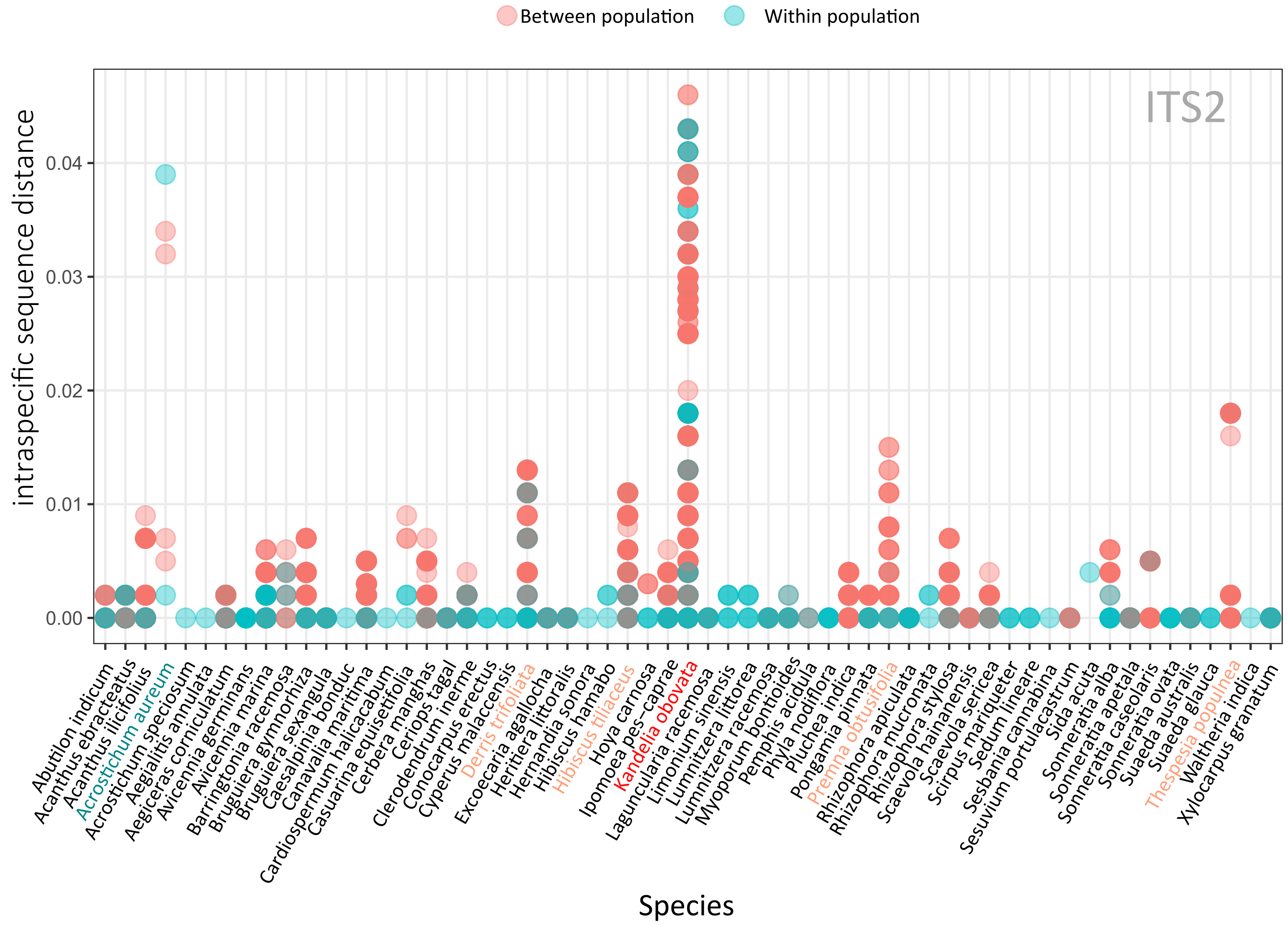

Figure S1. Scatter plots of intraspecific genetic distance within and between populations of the ITS2 marker. Red dots indicate values of between populations and green dots indicate those of within populations.

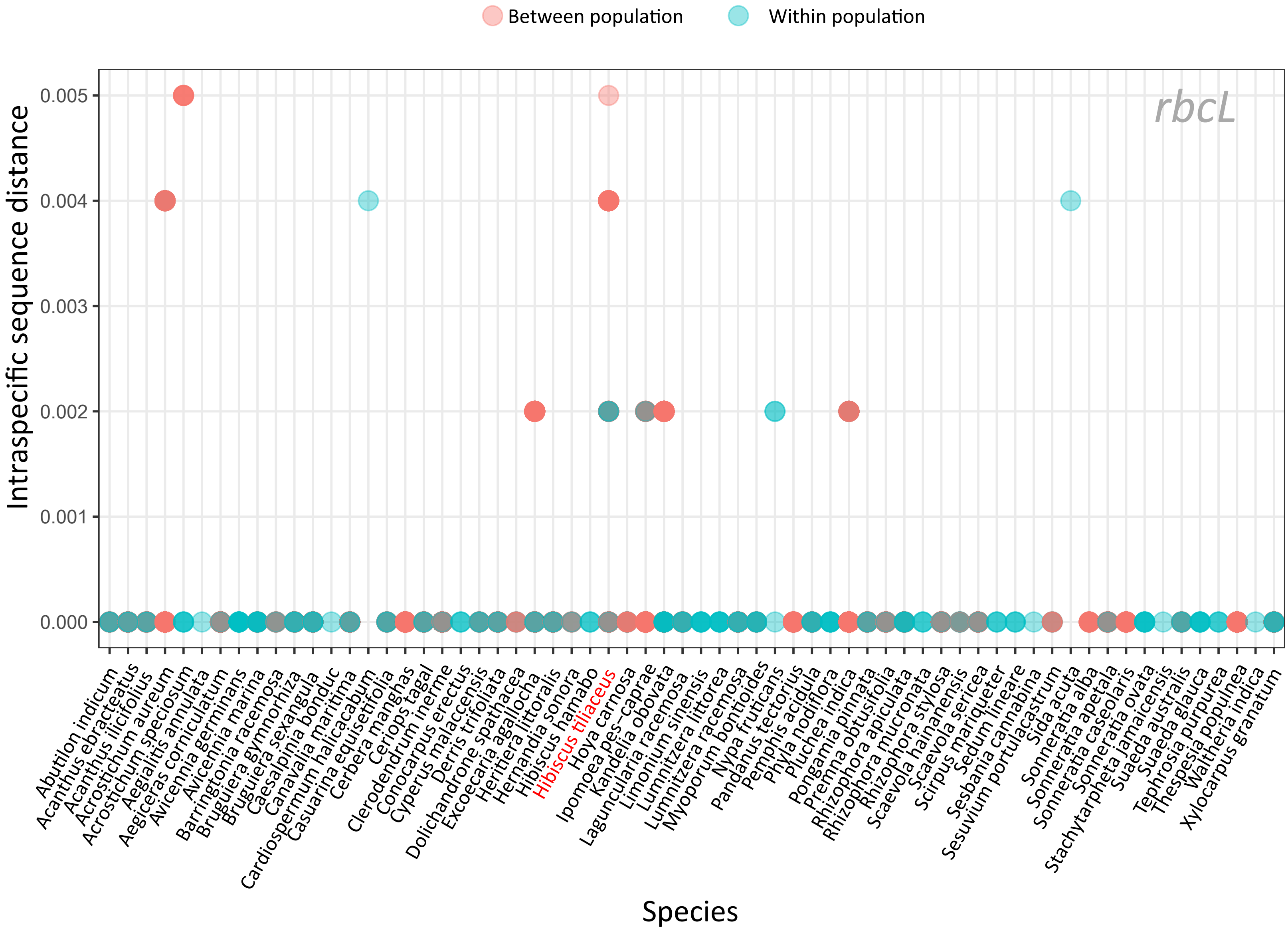

Figure S2. Scatter plots of intraspecific genetic distance within and between populations of the four genes. Red dots indicate values of between populations and green dots indicate those of within populations.

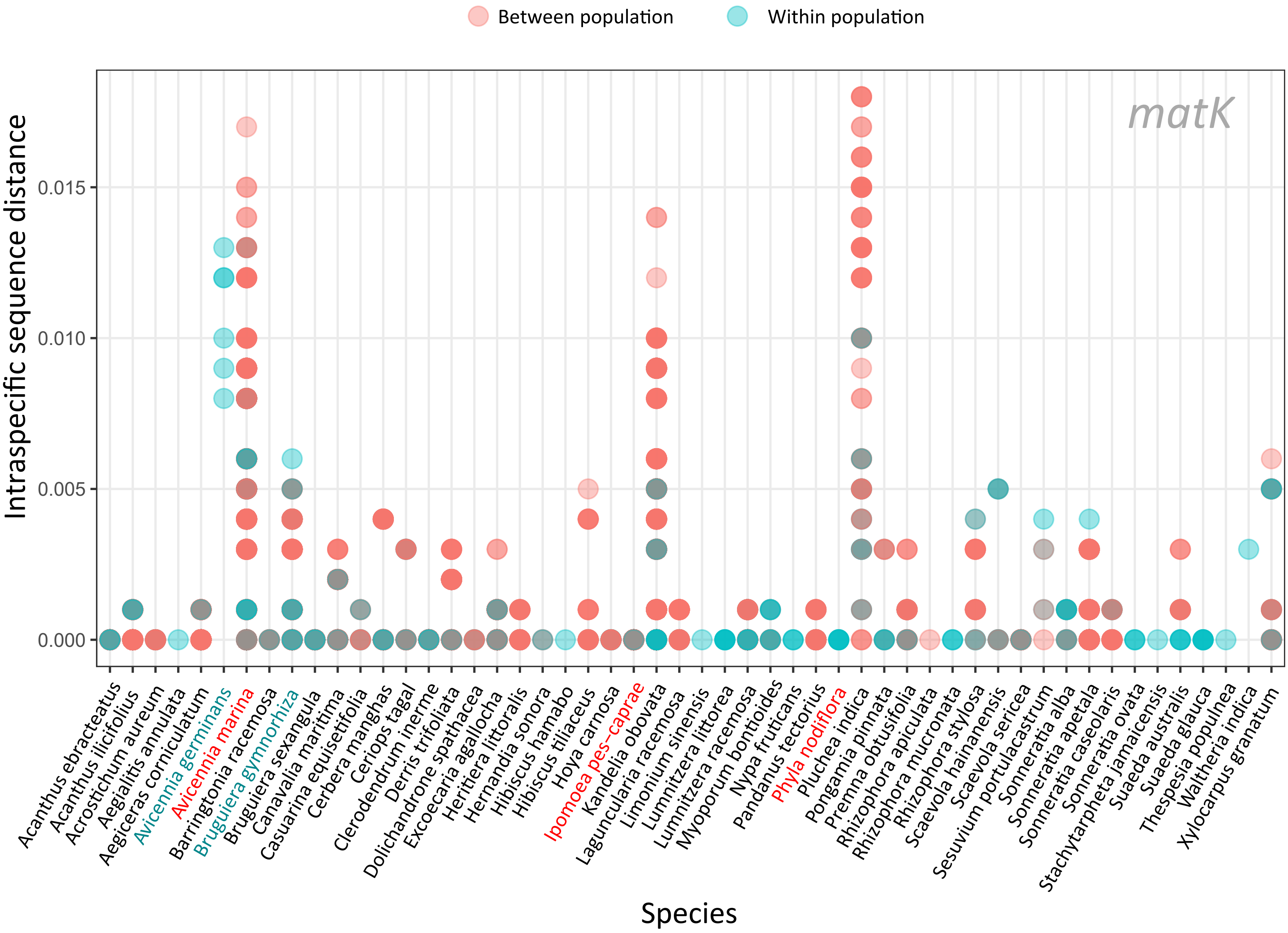

Figure S3. Scatter plots of intraspecific genetic distance within and between populations of the four genes. Red dots indicate values of between populations and green dots indicate those of within populations.

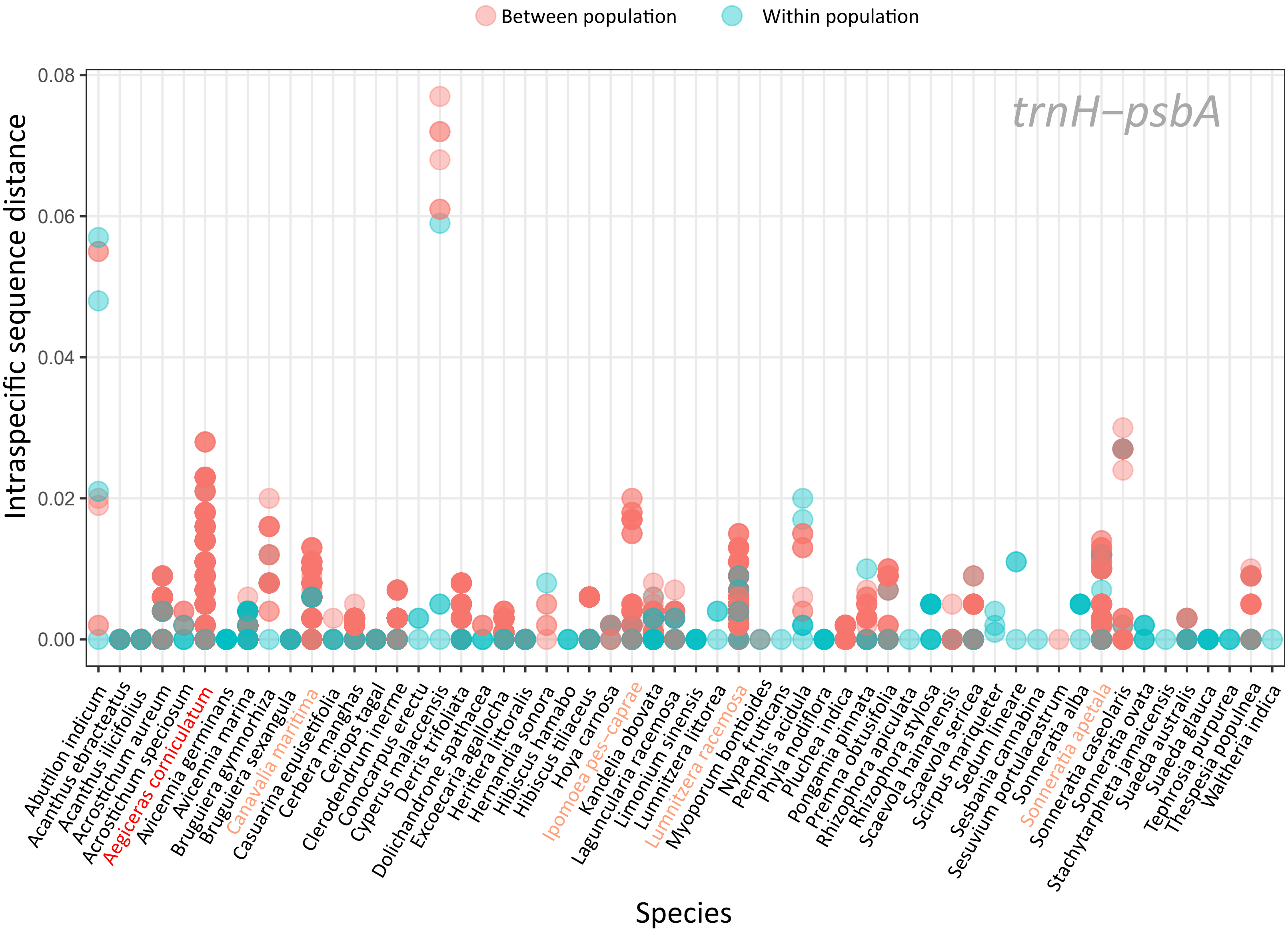

Figure S4. Scatter plots of intraspecific genetic distance within and between populations of the trnH-psbA marker. Red dots indicate values of between populations and green dots indicate those of within populations.

References

Chen, S., Yao, H., Han, J., Liu, C., Song, J., Shi, L., … Leon, C. (2010). Validation of the ITS2 region as a novel DNA barcode for identifying medicinal plant species. PLoS ONE, 5(1). doi:10.1371/journal.pone.0008613

Kress, W. J., & Erickson, D. L. (2007). A Two-Locus Global DNA Barcode for Land Plants: The Coding rbcL Gene Complements the Non-Coding trnH-psbA Spacer Region. PLoS ONE, 2(6), e508. doi: 10.1371/journal.pone.0000508
